# SETD7-mediated H3K4me1 activates ALDH1A3 to drive ferroptosis resistance in esophageal squamous cell carcinoma

**DOI:** 10.1038/s41419-025-08133-7

**Published:** 2025-11-07

**Authors:** Yang Feng, Xingyue Liu, Yangxia Wang, Mingyuan Zhang, Yangyang Ji, Longfeng Zhang, Yilu Tong, Fuyou Zhou, Hongyang Liu, Liang Ming, Junhu Wan

**Affiliations:** 1 Department of Clinical Laboratory, The First Affiliated Hospital of Zhengzhou University, Zhengzhou, China; 2 Key Clinical Laboratory of Henan province, Zhengzhou, China; 3 Anhui Province Key Laboratory of Immunology in Chronic Diseases, Bengbu Medical University, Bengbu, China; 4 Thoracic Department, Anyang Tumor Hospital, Henan Key Medical Laboratory of Precise Prevention and Treatment of Esophageal Cancer, Anyang, China; 5 Department of Obstetrics and Gynecology, The Third Affiliated Hospital of Zhengzhou University, Zhengzhou, China

**Keywords:** Cancer epigenetics, Cancer

## Abstract

SET domain-containing 7 (SETD7, also known as KMT7 or SET7/9), a histone lysine methyltransferase (HKMT) responsible for catalyzing histone H3 lysine 4 monomethylation (H3K4me1), has emerged as a key regulator in multiple cancers. However, the biological functions and epigenetic regulatory mechanisms of SETD7 in esophageal squamous cell carcinoma (ESCC) remain unclear. Our study found that SETD7 expression is significantly upregulated in ESCC tissues and positively correlates with clinical staging. Functional analyses revealed that SETD7 promotes ESCC cell proliferation and migration in vitro, while accelerating tumor growth in vivo. Additionally, SETD7 knockdown increased ESCC cell sensitivity to ferroptosis induction, indicating its dual functionality in tumorigenesis and ferroptosis resistance. Cleavage Under Targets and Tagmentation (CUT&Tag) sequencing analysis systematically mapped H3K4me1 modifications in ESCC cells, identifying ALDH1A3 (aldehyde dehydrogenase 1 family member A3) as a key downstream target. Mechanistically, SETD7-mediated H3K4me1 deposition at the ALDH1A3 promoter drives transcriptional activation, increasing the level of reduced coenzyme Q10 (CoQ10H₂) and inhibiting lipid peroxidation. This study reveals a novel epigenetic-metabolic axis (SETD7-H3K4me1-ALDH1A3/NADH/CoQ10H₂) that regulates ESCC progression and ferroptosis sensitivity, which highlights the clinical translational value of SETD7 in ESCC prognosis assessment and therapeutic development.

**Figure:**
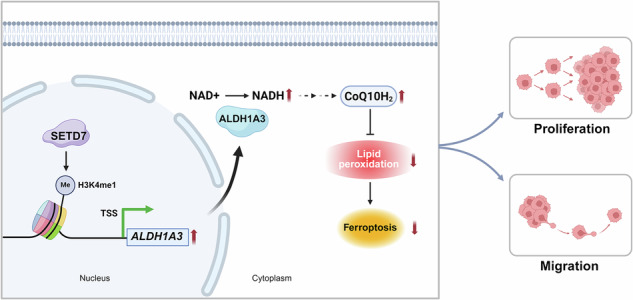
Schematic diagram illustrating the mechanism by which SETD7 accelerates ESCC progression through enhancing ferroptosis resistance. Created with BioRender.

Schematic diagram illustrating the mechanism by which SETD7 accelerates ESCC progression through enhancing ferroptosis resistance. Created with BioRender.

## Introduction

Esophageal cancer is a significant global health concern, ranking as the seventh leading cause of cancer-related deaths worldwide [1, 2]. Among the various subtypes of esophageal cancer, ESCC is the most prevalent, accounting for over 85% of all cases. This high prevalence is particularly notable in regions such as Eastern Asia and Eastern Africa [3]. The risk factors for ESCC are diverse and include smoking, alcohol consumption, dietary habits, and genetic predispositions, which vary significantly across different populations [4]. Despite advancements in treatment modalities, the prognosis for ESCC remains poor, with a five-year survival rate of less than 20% [5]. To address these critical challenges, the urgent development of novel therapies requires a deeper investigation into ESCC-associated genes to characterize their functional and mechanistic roles.

Epigenetic dysregulation, particularly aberrant histone modifications, has been identified as a hallmark of cancer progression [6, 7]. Consequently, epigenetically targeted therapies hold considerable promise as a novel therapeutic strategy. Histone lysine methylation dynamically modulates chromatin states, and disruption of the methylation landscape leads to oncogene activation or tumor suppressor silencing [8]. A previous study has reported that KMT2D promotes the progression of triple-negative breast cancer by catalyzing H3K4me1 deposition to enhance c-MYC transcriptional activity [9]. SETD7 is a histone lysine methyltransferase (HKMT) that catalyzes H3K4me1 to promote transcriptional activation [10, 11]. Increasing studies have confirmed that SETD7 plays a crucial role in regulating cancers [12–15]. SETD7 promotes bladder cancer progression and immune escape through the STAT3/PD-L1 signaling cascade [14]. The expression level of SETD7 in liver cancer tissue is significantly increased and is closely related to the pathological stage and size of the tumor [16]. By regulating the ERK/MAPK signaling pathway, SETD7 promotes epithelial-mesenchymal transition and tumor cell migration in triple-negative breast cancer [17]. However, the functional significance and mechanistic basis of SETD7 in ESCC progression remain unclear. Moreover, extant studies have predominantly focused on SETD7-mediated methylation of non-histone substrates (e.g., P53, FOXA1, KRAS), whereas its histone-modifying functions in cancer remain largely unexplored.

Ferroptosis is an iron-dependent cell death driven by lethal lipid peroxidation resulting from disrupted redox homeostasis [18]. Its core mechanism involves inhibiting antioxidant enzyme activity and abnormal accumulation of lipid peroxidation products. Tumor cells acquire ferroptosis resistance by activating antioxidant systems (e.g., SLC7A11-GPX4 axis, FSP1-CoQ10 axis) or epigenetic reprogramming, which has emerged as a critical mechanism underlying therapeutic failure [19–21]. In non-small cell lung cancer, H3K4me3 and H3K27ac regulate the expression of GPX4, which confers resistance to ferroptosis and leads to cisplatin resistance [22]. To overcome this challenge, novel strategies for drug sensitization are being explored. For instance, HMGA1 depletion was shown to sensitize ESCC to chemotherapeutic agents by promoting ferroptosis [23]. Another research confirmed that SETD7-mediated OTUB1 methylation promotes ferroptosis by alleviating OTUB1-dependent ferroptosis suppression [24]. However, whether SETD7 modulates ferroptosis through histone methylation remains unknown, especially in ESCC.

In this study, we aim to investigate the role of SETD7 as a histone lysine methyltransferase in ESCC progression. Our findings indicate that SETD7 is significantly overexpressed in ESCC and contributes to the development of malignant phenotypes. Furthermore, SETD7 functions as a ferroptosis suppressor, enhancing resistance to ferroptosis induction in ESCC. Our study reveals the molecular mechanism of SETD7-mediated epigenetic regulation in ESCC, highlighting its potential as a therapeutic target.

## Results

### SETD7 is highly expressed and correlates with clinicopathological parameters in ESCC patients

**Fig. 1:**
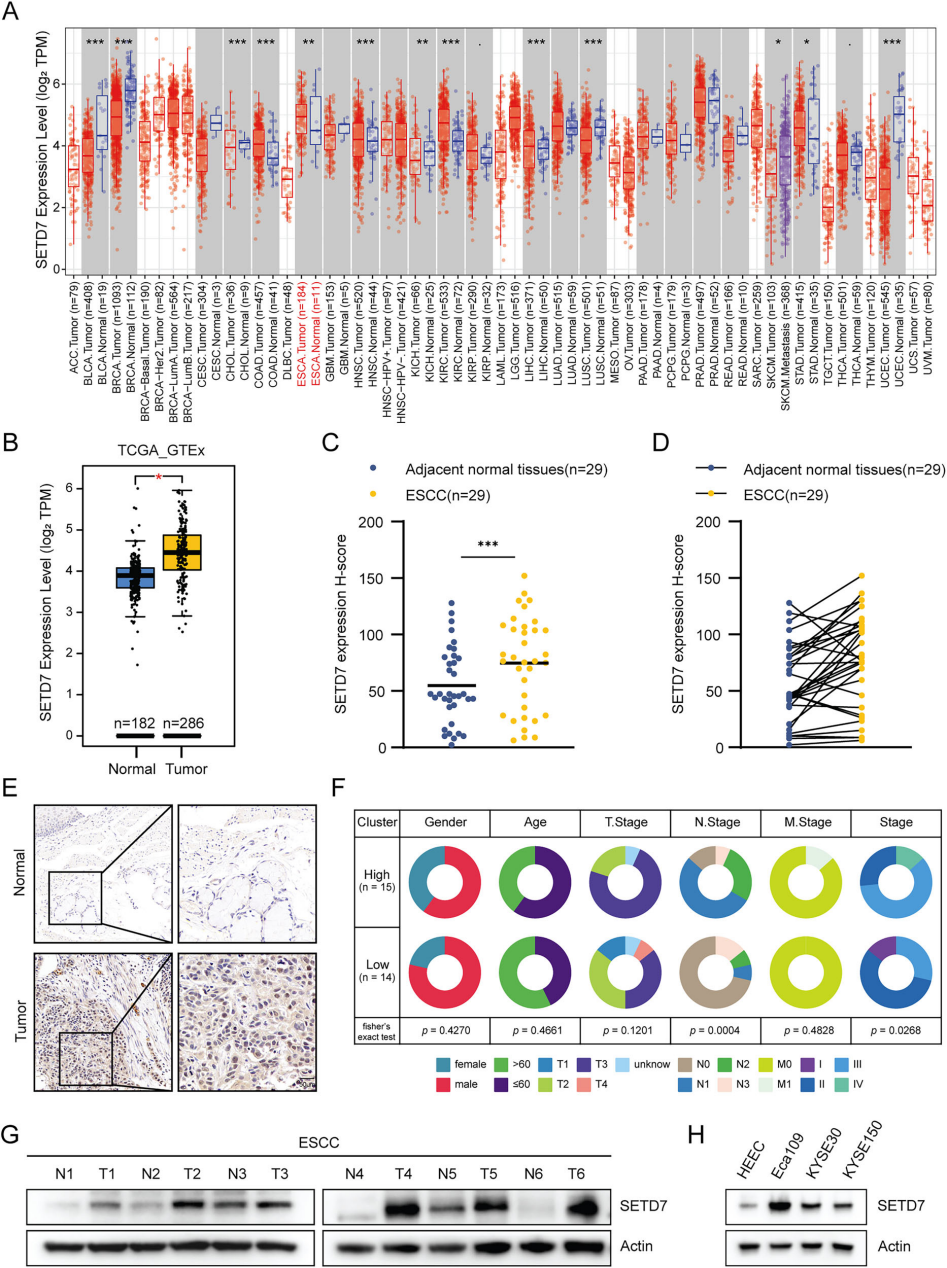
SETD7 is highly expressed in ESCC. **A** Pan-cancer analysis of SETD7 expression using the TIMER database. **B** Differential analysis of SETD7 expression between ESCC and normal tissues. **C**, **D** Quantification of SETD7 expression in ESCC and normal tissues was performed using H-score. **E** Representative IHC images show SETD7 staining in ESCC and normal tissues. Scale bar: 50 μm. **F** Clinicopathological characteristics and correlation with SETD7 expression in ESCC tissues. **G** Western blotting analysis of SETD7 expression in both ESCC and normal tissues. **H** Western blotting to assess SETD7 expression in normal esophageal epithelial cells (HEEC) and ESCC cell lines. Data are presented as means ± SD. *p < 0.05, **p < 0.01, ***p < 0.001, ****p < 0.0001.

To investigate the role of SETD7 in ESCC, we first assessed its expression levels using the TIMER database. Pan-cancer analysis revealed that SETD7 expression was significantly upregulated in ESCC tissues compared to normal tissues (Fig. 1A). A consistent result was obtained through comparative analysis of SETD7 expression profiles derived from TCGA and GTEx databases (Fig. 1B). Western blot analysis of ESCC tissues validated the bioinformatics findings (Fig. 1G). To evaluate clinical relevance, immunohistochemical (IHC) staining of SETD7 was performed on 29 pairs of adjacent and ESCC tissues, and the staining intensity was analyzed. The IHC results demonstrated significantly stronger SETD7 expression in ESCC specimens than in adjacent tissues (Fig. 1C, D), with representative IHC images shown in Fig. 1E. Subsequently, tissue samples were divided into two groups (SETD7 high/low) based on quantitative staining intensity and analyzed. As shown in Fig. 1F, the staining intensity of SETD7 was significantly correlated with clinical staging, while age and sex were not. Next, Western blotting was performed to compare SETD7 expression levels between Human Esophageal Epithelial Cells (HEEC) and ESCC cell lines (Eca109, KYSE30, KYSE150). Consistent with previous results, SETD7 exhibited significantly upregulated expression in ESCC cell lines (Fig. 1H). These results indicate that SETD7 expression is significantly upregulated in ESCC tissues compared with normal tissues and correlates with clinicopathological parameters, suggesting that SETD7 is associated with ESCC progression.

### SETD7 promotes ESCC cell migration and proliferation in vitro

**Fig. 2:**
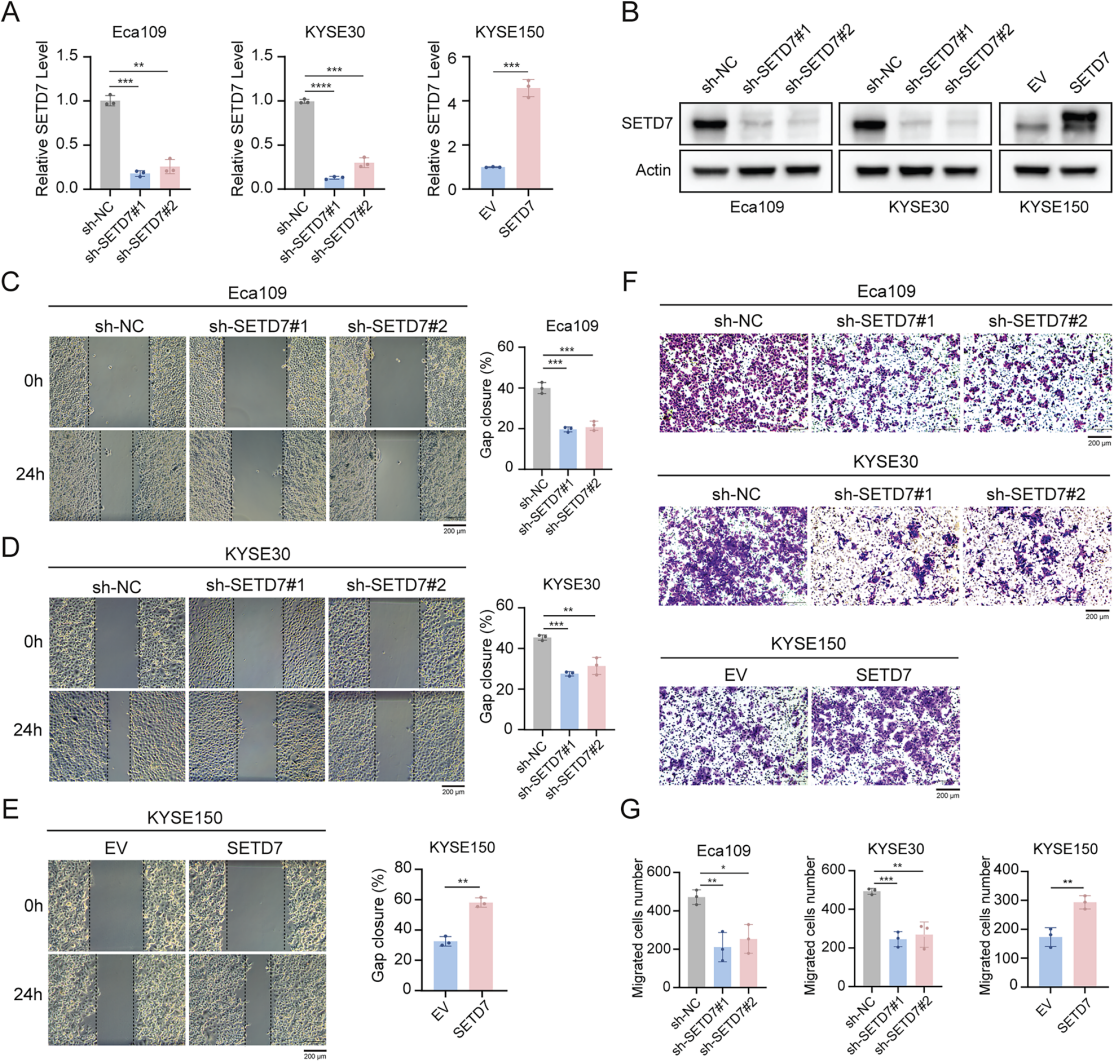
SETD7 promotes ESCC cell migration in vitro. **A**, **B** The knockdown and overexpression efficiency of SETD7 were verified using RT-qPCR and Western blotting. **C**–**E** Wound healing assays were used to assess the migration ability of ESCC cell lines with SETD7 knockdown. Scale bar: 200 μm. **F**, **G** Transwell migration assays were used to assess the migration ability of ESCC cell lines with SETD7 knockdown. Scale bar: 200 μm. Data are presented as means ± SD. *p < 0.05, **p < 0.01, ***p < 0.001, ****p < 0.0001.

**Fig. 3:**
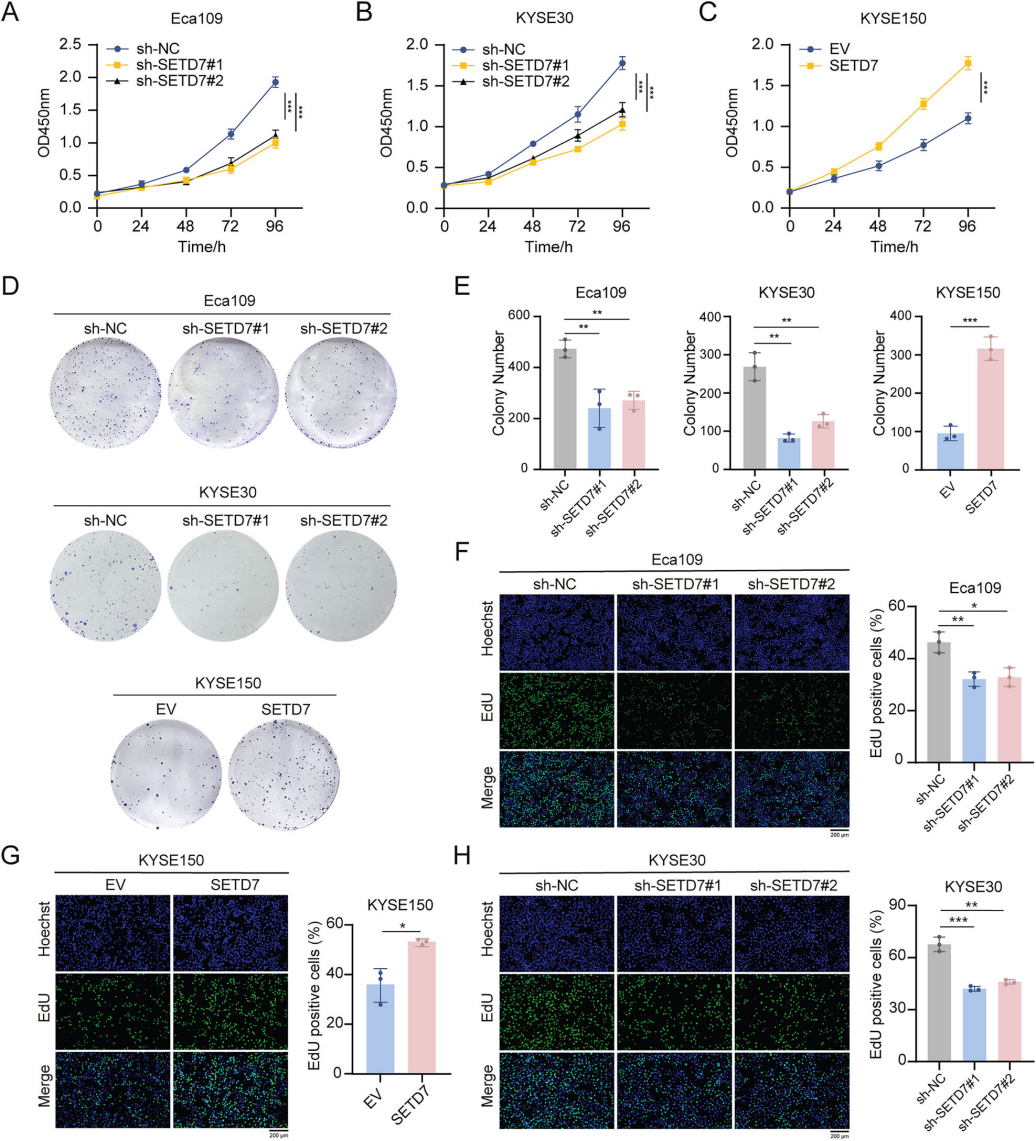
SETD7 promotes ESCC cell proliferation in vitro. **A**–**C** CCK-8 assays were used to evaluate proliferation in ESCC cell lines with SETD7 knockdown or overexpression. **D**, **E** Colony formation assays were performed to evaluate for proliferation in ESCC cell lines with SETD7 knockdown or overexpression. **F**–**H** The effect of SETD7 on the proliferation of ESCC cell lines was assessed via EdU assays. Scale bar: 200 μm. Data are presented as means ± SD. *p < 0.05, **p < 0.01, ***p < 0.001, ****p < 0.0001.

To examine the functional role of SETD7 in ESCC progression, we established stable cell lines with SETD7 knockdown or overexpression via lentiviral transduction. The knockdown and overexpression efficiencies were validated through RT-qPCR and Western blotting. Compared with negative controls, SETD7 expression was significantly reduced in Eca109 and KYSE30 knockdown models, while markedly increased in KYSE150 overexpression models (Fig. 2A, B). Wound healing and Transwell assays (Fig. 2C–G) were conducted to investigate SETD7’s regulatory role in ESCC cell migration. SETD7-silenced cells exhibited significantly impaired migratory potential in both assay systems compared to control groups. Conversely, SETD7-overexpressing cells exhibited increased migration capabilities. CCK-8 assays demonstrated that SETD7 knockdown significantly suppressed ESCC cell proliferation, whereas SETD7 overexpression markedly enhanced proliferative capacity (Fig. 3A–C). These findings were consistently corroborated by colony formation and EdU assays (Fig. 3D–H). The above results demonstrate that SETD7 functionally promotes the proliferation and migration of ESCC cells in vitro.

### SETD7 catalyzes H3K4me1 in ESCC cells

**Fig. 4:**
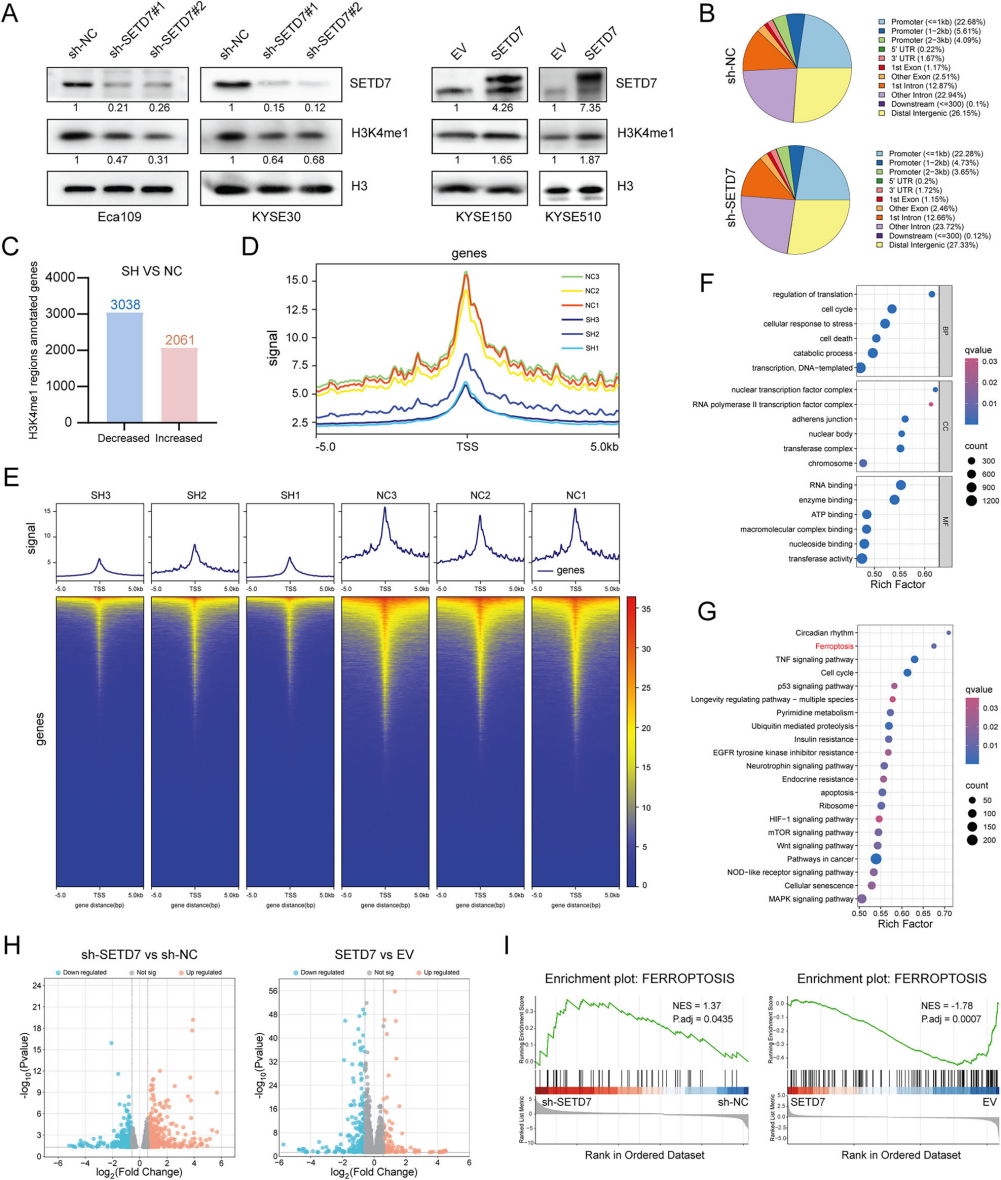
Integrated multi-omics analysis of SETD7-mediated H3K4me1 modification and transcriptional profiles in ESCC cells. **A** Western blotting was used to detect the H3K4me1 level in ESCC cell lines following SETD7 knockdown or overexpression. **B** The pie chart shows the distribution patterns of H3K4me1 peaks across genomic functional regions. **C** The number of genes associated with differentially enriched H3K4me1 peaks (|FC| > 1.5, *p* < 0.05). **D**, **E** The line plot and heatmap showing H3K4me1 signal enrichment around genomic TSS. **F**, **G** GO analysis and KEGG analysis for differential genes associated with differential H3K4me1 enrichment identified by CUT&Tag-seq. **H** Volcano plot showing RNA-seq-derived DEGs between SETD7-knockdown/overexpression and control groups (|FC| > 1.5, *p* < 0.05). **I** The GSEA analysis was used for RNA-seq data. The gene set was obtained from FerrDb.

SETD7, a member of the histone methyltransferase family characterized by its conserved SET domain, was initially identified as a histone H3 lysine 4 monomethyltransferase [10, 11]. To validate the hypothesis that SETD7 plays a regulatory role in histone modification within ESCC cells, we conducted Western blotting focusing on its primary methylation target, H3K4me1. The results indicated that H3K4me1 levels in ESCC cells were dynamically regulated by SETD7 knockdown or overexpression (Fig. 4A). Furthermore, we performed CUT&Tag-seq using an anti-H3K4me1 antibody to profile the genome-wide distribution patterns of H3K4me1 modifications and to assess changes in the H3K4me1 epigenetic landscape following SETD7 knockdown in ESCC cells. Compared to conventional chromatin immunoprecipitation sequencing, CUT&Tag-seq demonstrated superior signal-to-noise resolution as an advanced epigenomic profiling technology. The pie chart illustrated the distribution patterns of H3K4me1 peaks across genomic functional regions (Fig. 4B), revealing that approximately 32% of H3K4me1 peaks were enriched in promoter regions (≤3 kb). However, SETD7 knockdown did not alter the overall distribution proportions of these peaks across genomic functional regions. To assess alterations in the abundance of H3K4me1 modifications across the ESCC cell genome after SETD7 knockdown, we conducted a differential analysis of read counts between the two groups of H3K4me1 modification peaks. This analysis identified 6152 differentially enriched H3K4me1 peaks in the SETD7-silenced group compared to the control group. These peaks were annotated to 2061 genes with increased density of H3K4me1 modification and 3038 genes with decreased density of H3K4me1 modification, respectively (Fig. 4C). Notably, transcription start site (TSS) enrichment analysis revealed that SETD7 knockdown resulted in a significant reduction of H3K4me1 signals enriched at genomic TSS (Fig. 4D, E).

H3K4me1 is predominantly enriched in the promoter and enhancer regions of the genome and has been identified as a regulator of transcriptional activity. Then, RNA sequencing was utilized to evaluate the impact of SETD7 on the transcriptomic profiles of ESCC cells. Compared to their respective controls, SETD7-silenced Eca109 cells exhibited 1280 differentially expressed genes (DEGs), while SETD7-overexpressing KYSE150 cells displayed 3186 DEGs. Volcano plots illustrated significant differential expression patterns following SETD7 knockdown or overexpression (Fig. 4H). Based on these findings, we proposed that SETD7 regulates gene transcription by catalyzing H3K4me1, thereby influencing the progression of ESCC cells.

### SETD7 enhances the resistance of ESCC cells to ferroptosis

To elucidate the molecular mechanisms underlying SETD7-mediated H3K4me1 regulation in ESCC progression, Gene Ontology (GO) and Kyoto Encyclopedia of Genes and Genomes (KEGG) pathway analyses were performed on genes associated with differential H3K4me1 enrichment identified by CUT&Tag-seq. Functional analysis revealed significant enrichment in tumor-related pathways, including cellular response to stress, cell cycle, ferroptosis, and P53 pathway (Fig. 4F, G). Furthermore, we performed parallel functional enrichment analysis on DEGs of RNA-seq data (Fig. S1A–D), and the SETD7-overexpression group was also significantly enriched in the ferroptosis pathway. To further assess the association between SETD7 and ferroptosis pathways, Gene Set Enrichment Analysis (GSEA) was performed on RNA-seq data. Interestingly, GSEA revealed significant dysregulation of ferroptosis-related gene expression following SETD7 knockdown or overexpression (Fig. 4I).

**Fig. 5:**
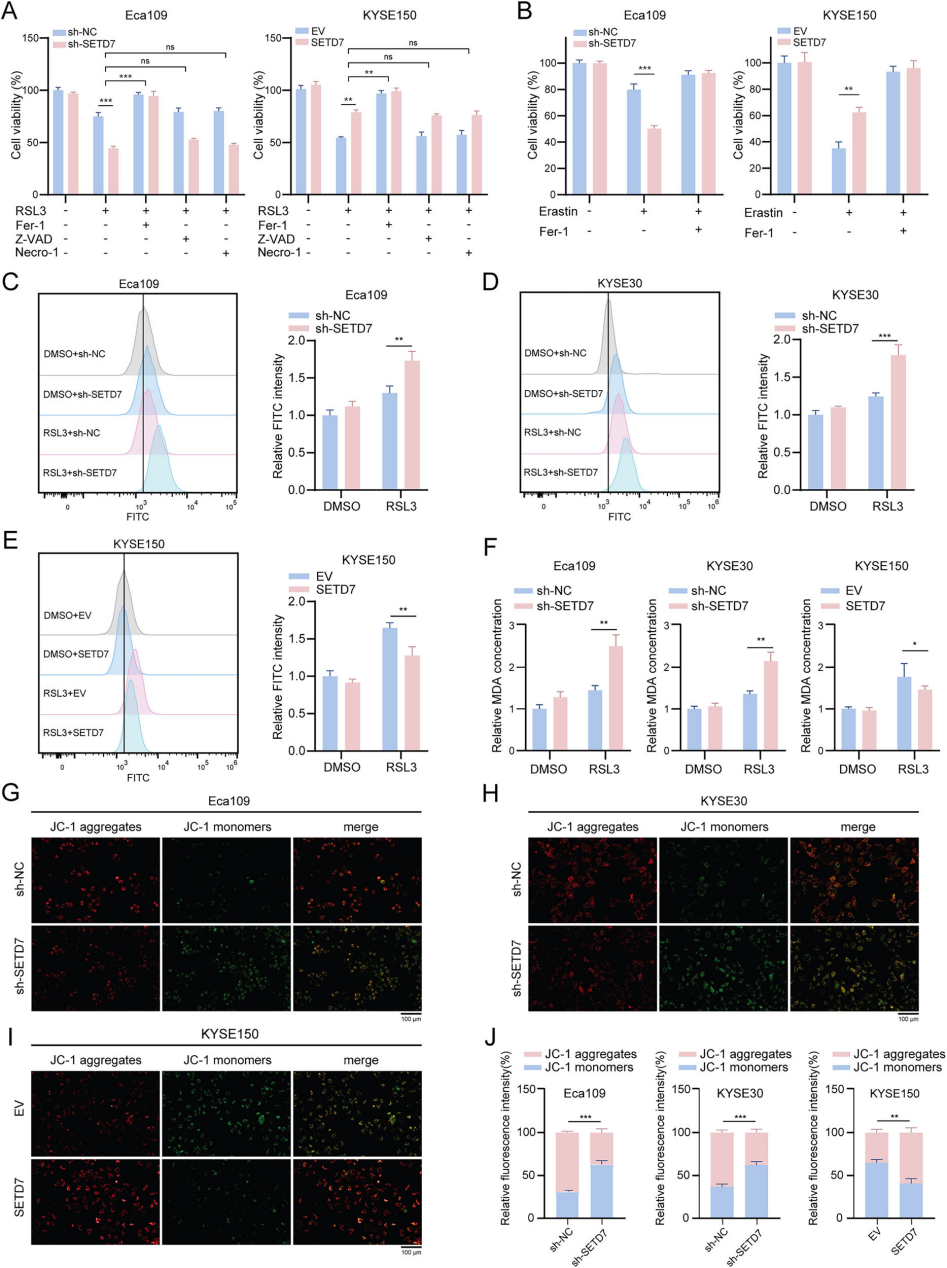
SETD7 confers ferroptosis resistance in ESCC cells. **A** Cell viability of Eca109 and KYSE150 cells treated with RSL3 (5 µM) alone or combined with Fer-1 (1 µM), Z-VAD (10 µM), or Necro-1 (1 µM) for 12 h. **B** Cell viability of Eca109 and KYSE150 cells treated with Erastin (10 µM) alone or combined with Fer-1 (1 µM) for 12 h. **C**–**E** Flow cytometry was performed to measure lipid ROS accumulation in ESCC cell lines with SETD7 knockdown or overexpression. **F** MDA assays revealed lipid peroxidation levels in ESCC cell lines with SETD7 knockdown or overexpression. **G**–**J** Representative fluorescent images (G–I) of JC-1-stained ESCC cells and statistical analysis (J) of fluorescence intensity. Scale bar: 100 μm. Data are presented as means ± SD. *p < 0.05, **p < 0.01, ***p < 0.001, ****p < 0.0001, ns not significant.

This consistent and prominent enrichment signal implicates ferroptosis as a key downstream pathway regulated by SETD7. Given the role of ferroptosis in regulating tumor progression, we prioritized investigating SETD7’s regulatory role in ferroptosis. To directly examine whether SETD7 modulates ferroptosis, ESCC cells with either SETD7 overexpression or knockdown were treated with RSL3, and cell viability was assessed. Notably, knockdown of SETD7 significantly sensitized ESCC cells to RSL3-induced ferroptosis, as evidenced by enhanced suppression of cell viability (Fig. 5A, left panel). Overexpression of SETD7 conferred resistance to RSL3-induced ferroptosis in ESCC cells (Fig. 5A, right panel). Importantly, this sensitization was specifically reversed by the ferroptosis inhibitor Ferrostatin-1 (Fer-1), but not by the apoptosis inhibitor Z-VAD-FMK (Z-VAD) or the necroptosis inhibitor Necrostatin-1 (Necro-1). Consistently, this SETD7-dependent ferroptosis resistance was also observed in ESCC cells treated with another ferroptosis inducer, Erastin (Fig. 5B and Fig. S2). Detection of ferroptosis-specific markers, such as malondialdehyde (MDA) and lipid reactive oxygen species (ROS), further validated the role of SETD7 in ferroptosis. Our results showed that SETD7 knockdown increased MDA and lipid ROS accumulation, whereas overexpression yielded opposing effects (Fig. 5C–F). In addition, JC-1 staining revealed a decline in the mitochondrial membrane potential of cells following SETD7 knockdown (Fig. 5G–J). These findings suggest that SETD7 may exert an anti-ferroptosis function.

### SETD7 promotes ALDH1A3 transcription by catalyzing H3K4me1

**Fig. 6:**
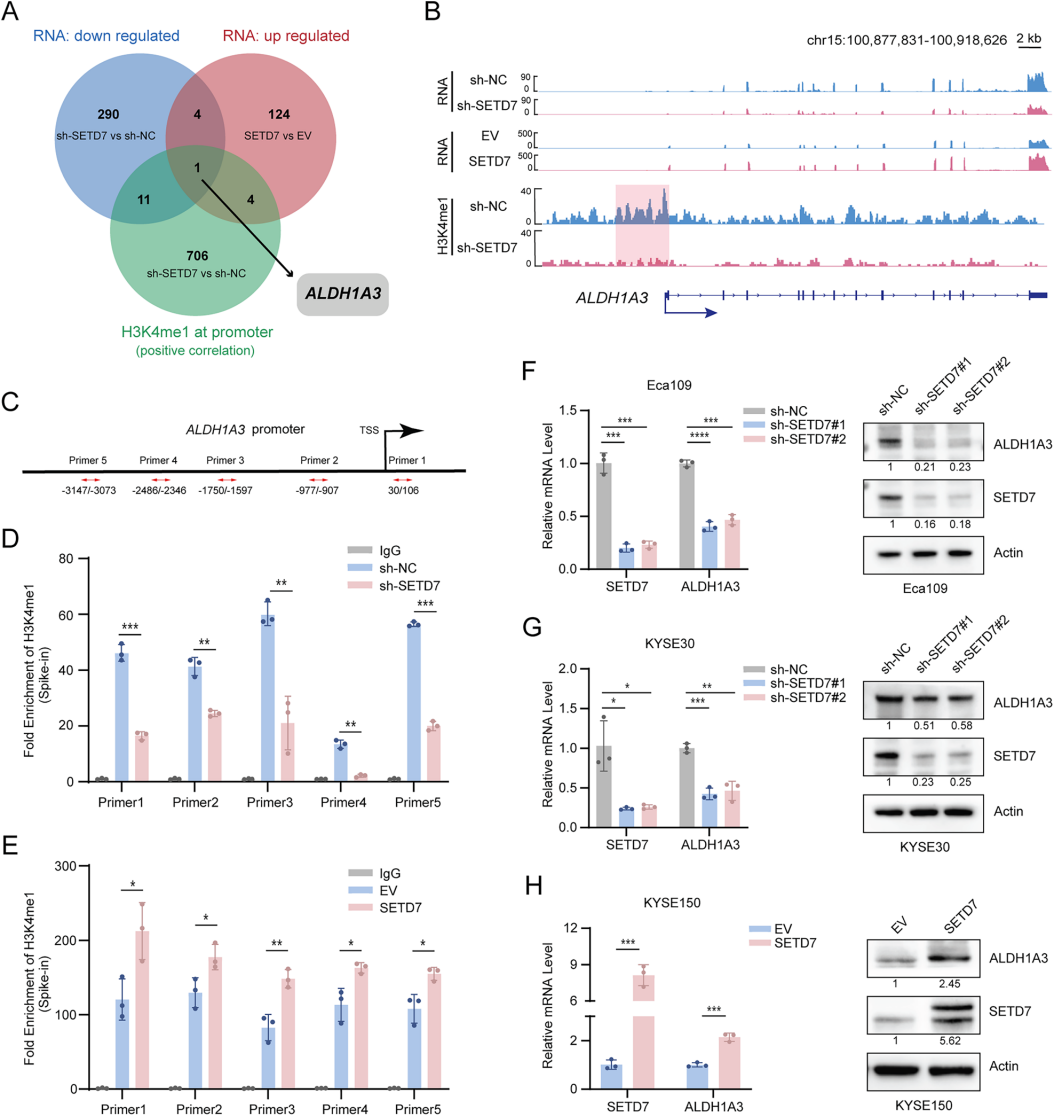
SETD7 promotes ALDH1A3 transcription by catalyzing H3K4me1. **A** Venn diagram showing the intersection between genes with differential H3K4me1 modification (CUT&Tag-seq) and differentially expressed genes (RNA-seq). |FC| > 1.5, *p* < 0.05. **B** Representative IGV browser tracks of RNA-seq and CUT&Tag-seq data. mRNA reads of ALDH1A3 in ESCC cell lines with SETD7 knockdown or overexpression(top), H3K4me1 signals at the ALDH1A3 promoter region in the SETD7-knockdown ESCC cell line (bottom). **C** Schematic diagram of primer locations in the ALDH1A3 gene promoter region for CUT&Tag-qPCR. **D**, **E** CUT&Tag-qPCR analysis showing H3K4me1 enrichment at the ALDH1A3 promoter in ESCC cell lines with SETD7 knockdown or overexpression. **F**–**H** RT-qPCR and Western blotting analyses were conducted to detect changes in ALDH1A3 mRNA and protein levels in ESCC cell lines with SETD7 knockdown or overexpression. Data are presented as means ± SD. *p < 0.05, **p < 0.01, ***p < 0.001, ****p < 0.0001.

To identify the key target genes regulated by SETD7 in ESCC cells, we conducted a comprehensive analysis by integrating CUT&Tag-seq and RNA-seq datasets. Considering the transcriptional activation role of H3K4me1, we identified genes exhibiting a positive correlation between promoter H3K4me1 signal intensity and SETD7 expression in the CUT&Tag-seq data. These genes overlapped with the DEGs exhibiting a positive correlation with SETD7 levels in the RNA-seq data. As illustrated in Fig. 6A, we identified ALDH1A3 as a key candidate gene, characterized by mRNA expression levels and promoter region H3K4me1 signal enrichment positively correlated with SETD7 expression. Subsequently, we employed the Integrative Genomics Viewer (IGV) to visualize and analyze the CUT&Tag-seq and RNA-seq data. As depicted in Fig. 6B, the knockdown of SETD7 resulted in a significant reduction of H3K4me1 signal enrichment at the ALDH1A3 promoter region, while ALDH1A3 mRNA reads remained positively correlated with SETD7 expression. Subsequently, primers were designed for these regions exhibiting reduced H3K4me1 signals and detected using CUT&Tag-qPCR assay (Fig. 6C). Consistent with the sequencing data, this analysis demonstrated a significant decrease in H3K4me1 enrichment at the ALDH1A3 promoter upon SETD7 knockdown (Fig. 6D). Conversely, SETD7 overexpression increased H3K4me1 enrichment at the same genomic locus (Fig. 6E). Moreover, we confirmed the regulatory function of SETD7-mediated H3K4me1 in the expression of ALDH1A3 (Fig. 6F–H and S3A–B). RT-qPCR analysis indicated that knockdown of SETD7 led to a reduction in ALDH1A3 mRNA levels, whereas overexpression of SETD7 increased its transcription. These findings were corroborated at the protein level through Western blot analysis. Collectively, these results demonstrate that SETD7 facilitates H3K4me1 at the ALDH1A3 promoter region, thereby enhancing its transcription and positively regulating ALDH1A3 expression.

### SETD7 confers ferroptosis resistance in ESCC cells via upregulated CoQ10H_2_

**Fig. 7:**
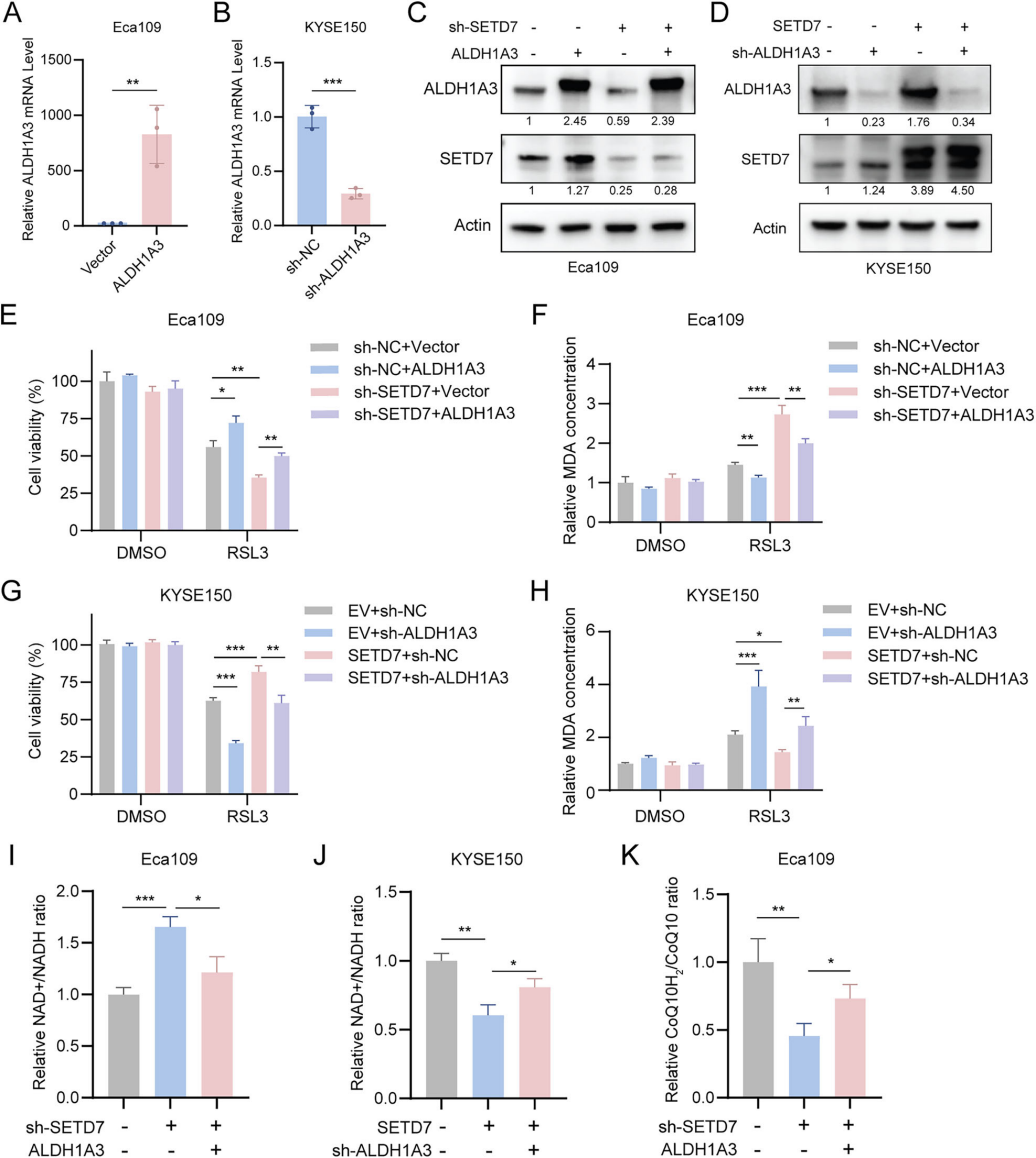
SETD7 regulates the sensitivity of ESCC cells to ferroptosis through the ALDH1A3/NADH/CoQ10H_2_ axis. **A**–**D** The knockdown and overexpression efficiency were validated by RT-qPCR and Western blotting. **E**, **F** ALDH1A3 overexpression rescued the impaired cell viability (E) and reduced MDA levels (F) in SETD7-knockdown Eca109 cells. **G**, **H** ALDH1A3 knockdown reversed the enhanced cell viability (G) and suppressed MDA levels (H) in SETD7-overexpressing KYSE150 cells. **I**, **J** The relative NAD + /NADH ratio in ESCC cell lines was quantified by enzymatic assay. **K** The relative ratio of CoQ10H_2_ to CoQ10 in ESCC cell lines was measured. Data are presented as means ± SD. *p < 0.05, **p < 0.01, ***p < 0.001, ****p < 0.0001.

To further validate whether the ferroptosis resistance regulated by SETD7 relies on ALDH1A3, we conducted rescue experiments by overexpressing ALDH1A3 in SETD7-knockdown ESCC cell lines and knocking down ALDH1A3 in SETD7-overexpressing cells. The efficiency of these genetic manipulations was confirmed through RT-qPCR and Western blotting (Fig. 7A–D). Under conditions that induce ferroptosis, ALDH1A3 overexpression significantly restored cell viability and reduced MDA levels in SETD7-knockdown cells (Fig. 7E, F). Conversely, ALDH1A3 knockdown diminished the survival advantage and increased MDA accumulation in SETD7-overexpressing cells (Fig. 7G, H). These results demonstrated that the ferroptosis resistance conferred by SETD7 is mechanistically dependent on ALDH1A3 expression.

ALDH1A3 catalyzes the oxidation of retinaldehyde to retinoic acid, accompanied by the production of NADH [25]. NADH serves as a crucial cofactor for the FSP1-mediated reduction of CoQ10 (oxidized coenzyme Q10) to CoQ10H_2_ (reduced coenzyme Q10) [26]. Notably, a recent study has demonstrated that ALDH1A3 modulates ferroptosis susceptibility by regulating the intracellular NADH pool, which indirectly influences CoQ10H_2_ levels [27]. Building upon this evidence, we propose that SETD7 modulates ferroptosis sensitivity through the ALDH1A3/NADH/CoQ10H_2_ axis. In SETD7-knockdown ESCC cells, we observed a marked increase in the NAD + /NADH ratio, which was effectively rescued by ALDH1A3 overexpression (Fig. 7I). Similarly, SETD7 overexpression led to elevated NADH levels, whereas ALDH1A3 knockdown reversed this effect (Fig. 7J). Further analysis of CoQ10 levels demonstrated that SETD7 knockdown decreased CoQ10H_2_ levels, which were partially restored by ALDH1A3 overexpression (Fig. 7K). Collectively, these data establish the ALDH1A3/NADH/CoQ10H_2_ axis as a critical metabolic hub through which SETD7 regulates ferroptosis sensitivity in ESCC.

### SETD7 drives ESCC cell proliferation and migration through ALDH1A3-mediated ferroptosis resistance

**Fig. 8:**
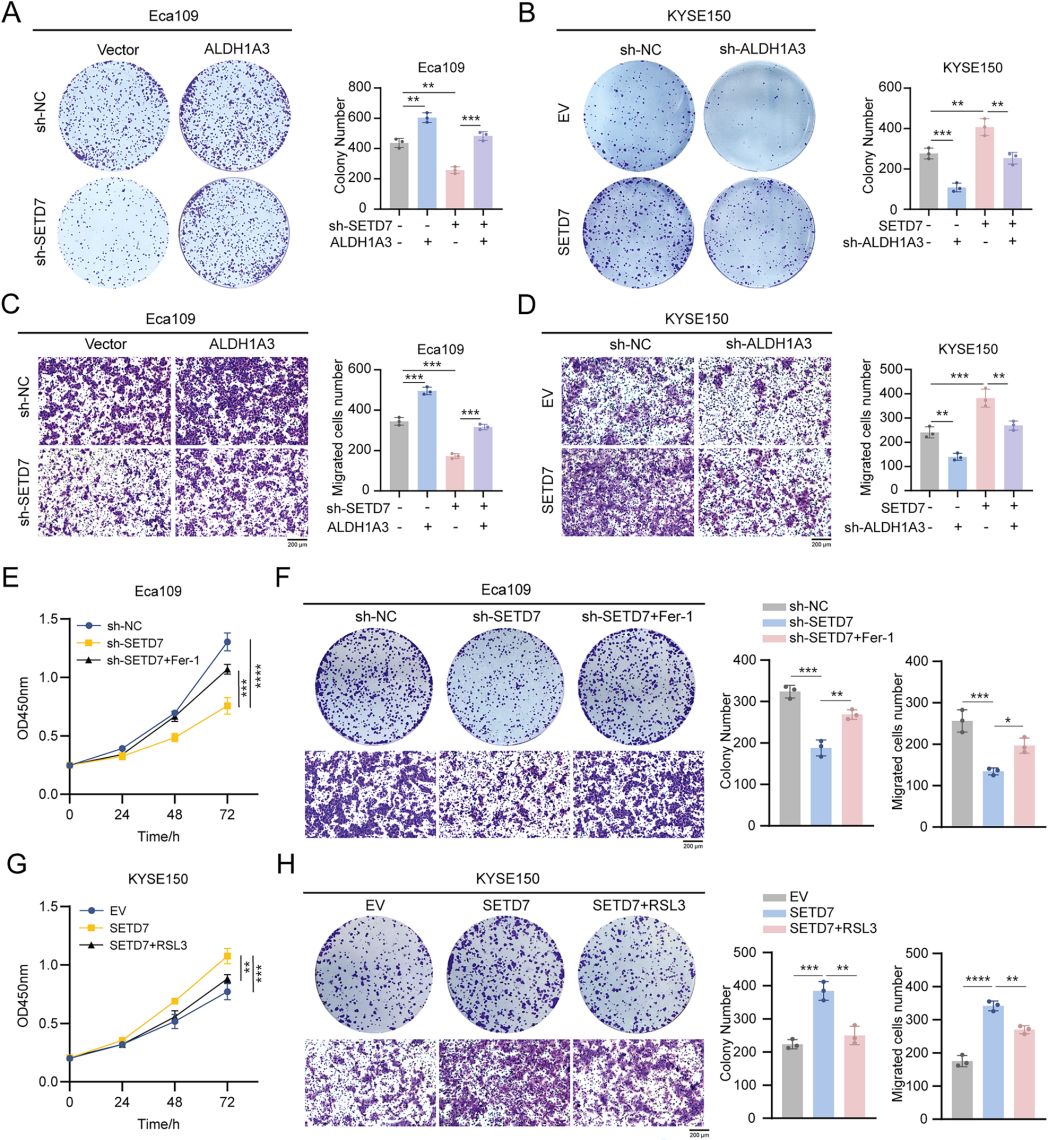
SETD7 promotes ESCC progression via ALDH1A3-mediated ferroptosis resistance. **A** Colony formation assay showing that ALDH1A3 overexpression restored proliferation suppressed by SETD7 knockdown. **B** Colony formation assay revealing that ALDH1A3 knockdown reversed the enhanced proliferation of SETD7-overexpressing ESCC cells. **C** Transwell assay assessing the effect of ALDH1A3 overexpression on migration in SETD7-knockdown ESCC cells. Scale bar: 200 μm. **D** Transwell assay evaluating the effect of ALDH1A3 knockdown on migration in SETD7-overexpressing ESCC cells. Scale bar: 200 μm. **E** Proliferation of SETD7-knockdown ESCC cells treated with Fer-1 analyzed by CCK-8 assay. **F** Colony formation and Transwell migration assays examining Fer-1 effects on proliferation and migration of SETD7-knockdown ESCC cells. Scale bar: 200 μm. **G** Proliferation of SETD7-overexpressing ESCC cells treated with RSL3 analyzed by CCK-8 assay. **H** Colony formation and Transwell migration assays examining RSL3 effects on proliferation and migration of SETD7-overexpressing ESCC cells. Scale bar: 200 μm. Data are presented as means ± SD. *p < 0.05, **p < 0.01, ***p < 0.001, ****p < 0.0001.

To validate the relationship between SETD7-regulated malignant phenotypes and ALDH1A3 expression in ESCC, we conducted functional assays on ESCC cells co-transfected with SETD7 and ALDH1A3 constructs. Colony formation assays showed that ALDH1A3 overexpression in SETD7-knockdown cells restored impaired clonogenic capacity (Fig. 8A), while its knockdown diminished enhanced clonogenicity in SETD7-overexpressing cells (Fig. 8B). Transwell (Fig. 8C) and wound healing (Fig. S4A) assays further confirmed that ALDH1A3 overexpression reversed migration suppression in SETD7-knockdown cells, whereas ALDH1A3 knockdown attenuated migration potentiation in SETD7-overexpressing cells (Fig. 8D, S4B). Consistently, ALDH1A3 overexpression in SETD7-knockdown KYSE30 cells reversed the inhibition of both clonogenicity and migratory ability (Fig. S5). These results show that ALDH1A3 is the primary effector molecule driving SETD7-mediated malignant phenotypes in ESCC.

Given that SETD7 enhances ferroptosis resistance via the ALDH1A3/CoQ10H₂ axis, we examined whether ferroptosis modulation affects SETD7-driven phenotypes. Notably, treatment with the ferroptosis inhibitor Fer-1 (1 μM) in SETD7-knockdown cells restored the suppressed proliferative (colony formation) and migratory (Transwell) capacities (Fig. 8E, F). Conversely, the inducer RSL3 (3 μM) inhibited the enhanced proliferation and migration conferred by SETD7 overexpression (Fig. 8G, H). These results collectively demonstrate that ferroptosis resistance is critical for SETD7-mediated ESCC progression.

### SETD7 promotes ESCC progression in vivo

**Fig. 9:**
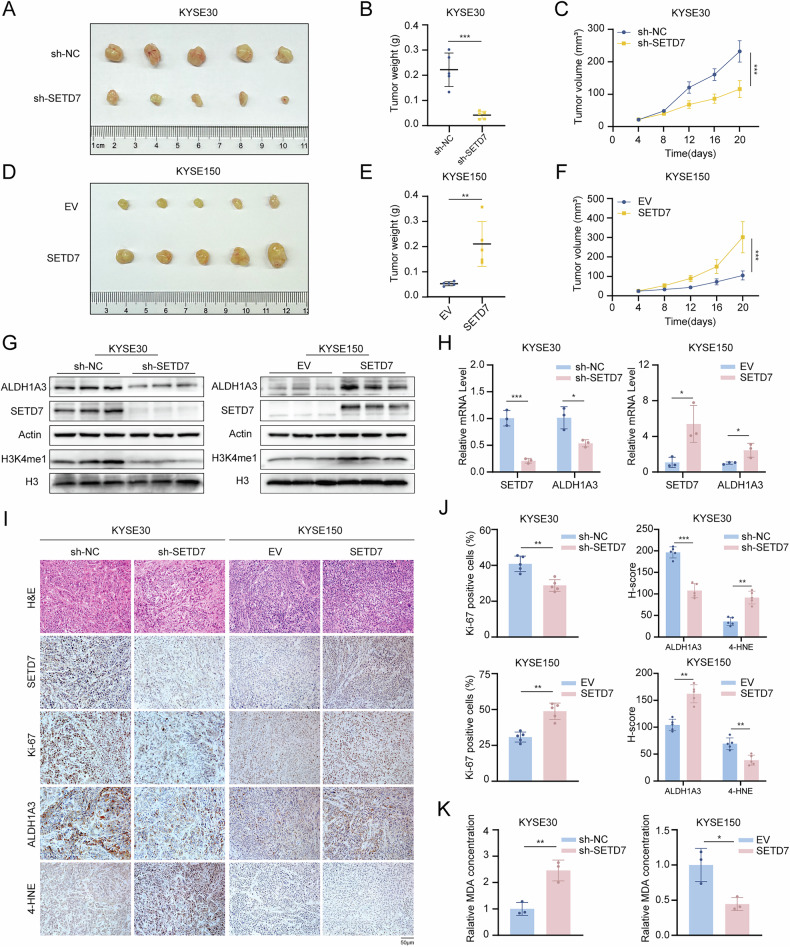
SETD7 promotes ESCC progression in vivo. **A**, **D** Images of subcutaneous xenograft tumors excised from nude mice (*n* = 5). **B**, **E** Tumor weight across experimental groups. **C**, **F** Growth curves of subcutaneous xenograft tumors. **G** Western blotting analysis of SETD7, ALDH1A3, and H3K4me1 protein levels in subcutaneous xenograft tumor tissues. **H** RT-qPCR analysis of SETD7 and ALDH1A3 mRNA levels in subcutaneous xenograft tumors. **I** Representative images of H&E staining and IHC staining for SETD7, Ki67, ALDH1A3, and 4-HNE in subcutaneous xenograft tumors. Scale bar: 50 μm. **J** Quantification of IHC staining for Ki67, ALDH1A3, and 4-HNE in subcutaneous xenograft tumors. **K** MDA levels analysis in subcutaneous xenograft tumor tissues. Data are presented as means ± SD. *p < 0.05, **p < 0.01, ***p < 0.001, ****p < 0.0001.

To validate the in vitro findings, xenograft tumor models were established by subcutaneously injecting nude mice with ESCC cells that stably expressed SETD7-knockdown or -overexpression constructs. The results demonstrated that SETD7 knockdown significantly inhibited tumor growth compared to controls, evidenced by reduced tumor volume and weight (Fig. 9A–C). Conversely, SETD7 overexpression markedly increased tumor volume and weight (Fig. 9D–F). Furthermore, IHC analysis revealed a decreased proportion of Ki67-positive cells in SETD7-knockdown tumors and an elevated Ki67 index in SETD7-overexpressing tumors (Fig. 9I, J), confirming that SETD7 promotes tumor cell proliferation in vivo. To determine whether SETD7 regulates ALDH1A3 expression in vivo, we analyzed xenograft tumor tissues using RT-qPCR, Western blotting, and IHC staining. These results indicated that SETD7 knockdown significantly decreased ALDH1A3 mRNA and protein levels, whereas SETD7 overexpression increased them (Fig. 9G–J). Additionally, MDA assays and IHC staining revealed that SETD7 knockdown significantly increased levels of MDA and 4-hydroxynonenal (4-HNE), while SETD7 overexpression reduced these lipid peroxidation products (Fig. 9I–K). The above results confirm that SETD7 accelerates ESCC tumor growth and suppresses lipid peroxidation in vivo, while also establishing its role as a positive regulator of ALDH1A3 expression.

## Discussion

ESCC is a life-threatening disease with a poor prognosis, primarily due to late-stage diagnosis and the limited effective therapies available for advanced cases [2, 28]. Epigenetic alterations, such as DNA hypermethylation and dysregulation of histone modification, have been detected in over 60% of ESCC patients [29]. The reversible nature of these epigenetic alterations makes them promising therapeutic targets. Histone lysine methylation catalyzed by HKMTs represents a key epigenetic regulatory mechanism. Multiple HKMTs, including EZH2 and KMT2D, have been reported to play critical roles in cancer progression [30, 31]. Notably, inhibitors targeting the histone methyltransferase EZH2 have entered clinical trials, demonstrating the feasibility of modulating HKMTs for cancer therapy [32]. As a member of the HKMT family, SETD7 plays critical roles in tumorigenesis across multiple cancer types [12–17]. This study found that SETD7 was significantly overexpressed in ESCC tissues and cell lines, and its expression correlated with clinical staging. In vitro functional assays demonstrated that SETD7 knockdown inhibits ESCC cell proliferation and migration, while SETD7 overexpression promotes these processes. Furthermore, xenograft models showed that SETD7 promotes ESCC tumor growth in vivo. These findings indicate that SETD7 exerts pro-tumorigenic functions in ESCC by regulating malignant phenotypes.

The SET domain serves as the core functional module of HKMTs. As an HKMT, SETD7 specifically catalyzes the monomethylation of lysine 4 on histone H3 [10]. SETD7-mediated H3K4me1 modifications are involved in the progression of various diseases. In diabetic retinopathy research, SETD7 promotes KEAP1 expression by enhancing H3K4me1 at the KEAP1 promoter region, accelerating retinal endothelial cell damage [33]. Another study demonstrated that SETD7-mediated H3K4me1 activates pro-fibrotic-related gene transcription, and SETD7 knockdown alleviates fibrosis [34]. However, studies on SETD7 in cancer research have long focused on its non-histone substrates. In clear cell renal cell carcinoma, SETD7 catalyzes the methylation of lysine 5 and 300 on the TAF7 protein, leading to TAF7 deubiquitination and stabilization [12]. Another study revealed that SETD7-mediated methylation at the FOXA1-K270 site suppresses its transcriptional activity [13]. In contrast to previous studies focusing on SETD7-mediated methylation of non-histone substrates, this study systematically investigated its histone methyltransferase function in ESCC using CUT&Tag-seq. We demonstrated that SETD7 knockdown significantly reduced H3K4me1 signals at TSS regions and identified ALDH1A3 as its critical downstream target gene. While previous studies reported KDM4C or *trans*-3-indoleacrylic acid regulates ALDH1A3 expression through different mechanisms [27, 35], we revealed a novel epigenetic regulatory mode where SETD7-mediated H3K4me1 directly promotes ALDH1A3 transcription.

Increasing evidence indicates that ferroptosis plays important roles in tumor initiation, progression, metastasis, and chemoresistance [36–39]. Tumor cells develop adaptive resistance to ferroptosis, leading to a survival advantage and malignant progression [40, 41]. Studies suggest that epigenetic modifications regulate cancer cell responsiveness to ferroptosis through transcriptional or translational regulation [42]. SETD2 modulates FECH expression via H3K36me3-mediated transcriptional regulation, influencing ferroptosis sensitivity in renal cancer cells. KDM3B upregulates SLC7A11 expression through H3K9me1 modification, countering Erastin-induced ferroptosis [43]. Our study found that SETD7 knockdown enhanced ESCC cell sensitivity to ferroptosis induced, which is critical for SETD7-mediated ESCC progression. Mechanistically, SETD7 enhances resistance to ferroptosis in ESCC cells through H3K4me1-mediated upregulation of ALDH1A3. As a member of the Aldehyde dehydrogenase family, ALDH1A3 plays a crucial role in cellular detoxification and oxidative stress responses [25]. Accumulating evidence highlights the critical and diverse roles of various ALDH isoforms in modulating cellular sensitivity to ferroptosis [44–46]. These enzymes directly regulate redox homeostasis and lipid peroxidation by detoxifying reactive aldehydes (e.g., 4-HNE, MDA) and generating NADH/NADPH. Studies have shown that ALDH1A3 promotes the synthesis of CoQ10H₂ via its metabolic product NADH, thereby counteracting ferroptosis [27]. The FSP1-CoQ10H₂ axis functions as a parallel anti-ferroptosis system alongside the SLC7A11-GSH-GPX4 axis, where CoQ10H₂ suppresses lipid peroxidation and ferroptosis by scavenging lipid radicals through its reducing capacity [47]. This study revealed that SETD7 upregulates ALDH1A3 expression, which generates NADH and elevates CoQ10H₂ levels. By enhancing CoQ10H₂-dependent antioxidant activity, SETD7 restrains lipid peroxidation and thereby confers ferroptosis resistance in ESCC cells.

In conclusion, we discovered the critical role that SETD7 plays in promoting ESCC progression. This finding demonstrates the theoretical feasibility of targeting SETD7 to suppress malignant phenotypes in cancer cells. Additionally, our study provides mechanistic insights into SETD7-dependent epigenetic regulation in ESCC, confirming the regulatory role of the SETD7-H3K4me1-ALDH1A3 axis in modulating ferroptosis sensitivity, which provides a molecular basis for developing targeted therapies against ESCC.

Meanwhile, we recognize some limitations of the present study. Firstly, the therapeutic efficacy of SETD7 inhibitors in ESCC models remains unassessed, which will be a critical focus for future translational research. Secondly, whether SETD7-regulated ferroptosis could enhance the efficacy of chemotherapy and immunotherapy in ESCC warrants further exploration. Future studies require a comprehensive investigation to elucidate how SETD7 orchestrates ferroptosis regulation in cancers.

## Materials and methods

### Patients and clinical samples

Surgical specimens were taken from esophageal carcinoma patients admitted to Anyang Cancer Hospital. All subjects were diagnosed by the Pathology Department of Anyang Cancer Hospital and did not undergo surgery, radiation therapy, or chemotherapy before surgery. Some tissues were immediately frozen and stored at −80 °C for subsequent Western blot analysis. The remaining tissues were fixed with 4% paraformaldehyde for IHC analysis.

### IHC analysis

Paraffin-embedded tissues were baked in a 60 °C oven for 40 min, dewaxed in xylene. Antigen retention was performed in a sodium citrate buffer (pH 6.0) for 15 min. Then, the tissues were incubated at room temperature with 3% hydrogen peroxide for 10 min and blocked with 3% bovine serum albumin for 30 min. Then, the tissue slices were incubated overnight with the primary antibodies at 4 °C. The tissues were incubated with horseradish peroxidase–conjugated secondary antibodies at room temperature for 30 min. Then, hematoxylin was used for counterstaining, and 70% alcohol was used for dehydration. Finally, images were captured under a microscope.

### Cell culture and transfection

The human ESCC cell lines (Eca109, KYSE30, KYSE150, and KYSE510) and the human normal esophageal epithelial cell line (HEEC) were purchased from the Institute of Cells, Shanghai Academy of Life Sciences. All cell lines were cultured at 37 °C with 5% CO_2_. HEEC was cultured in DMEM. Eca109, KYSE30, KYSE150 and KYSE510 were cultured in RPMI-1640. DMEM and RPMI-1640 were supplemented with 10% fetal bovine serum and 1% penicillin/streptomycin. For ferroptosis-related experiments, cells were treated with RSL3 (#HY-100218A, MedChemExpress), Ferrostatin-1 (#HY-100579, MedChemExpress), Z-VAD-FMK (#HY-16658B, MedChemExpress), or Necrostatin-1 (#HY-15760, MedChemExpress).

Cell lines with stable knockdown of SETD7 and ALDH1A3, as well as SETD7 overexpression, were established using lentivirus vectors (Genechem, China). Puromycin was used to select the transfected cells for 2 weeks. The overexpression plasmid of ALDH1A3 was purchased from Genechem (Shanghai, China). Lipofectamine 2000 (Invitrogen, USA) was used to terminate all transfections. All the target sequences were shown in Supplementary Table S2.

### RNA extraction and RT-qPCR assays

Total RNA of cells was extracted using TRIzol reagent (Takara, Japan). RNA was reverse transcribed into cDNA using the PrimeScript RT reagent Kit (Takara, Japan), and RT-qPCR assays were performed on the LightCycler 480 system (Roche, Swiss) using TB-Green PCR Master Mix Kit (Takara, Japan). All operations were conducted following the manufacturer’s instructions. All the primers used are shown in Supplementary Table S1.

### Western blotting and antibodies

Cells and tissues were lysed using RIPA Lysis Buffer (Beyotime, China) and Protease inhibitor (Beyotime, China). The BCA assay kit was used to measure the protein concentration. An equal amount of protein was separated on SDS-PAGE gels and then transferred to PVDF membranes. The membranes were then incubated with 5% nonfat milk for 1 h, followed by incubation with primary antibodies overnight at 4 °C. HRP-conjugated secondary antibodies were then washed three times, and the membranes were added for incubation for 1 h at room temperature. The blots were visualized with the ECL kit (Epizyme, China). The antibodies used in this study were listed as follows: anti-SETD7 (#SC-390823, Santa Cruz), anti-H3K4me1 (#PTM-5158, PTM Biolabs), anti-ALDH1A3 (#25167-1-AP, Proteintech), anti-Ki-67 (#28074-1-AP, Proteintech), anti-4-HNE (#HY-P81208, MedChemExpress), anti-Histone H3 (#YM3038, Immunoway), anti-β-actin (#66009-1-Ig, Proteintech).

### Cell Counting Kit-8 (CCK-8), colony formation, and EdU assays

For CCK-8 assays, 2,000 cells were seeded per well in 96-well plates. 10 μL of the CCK-8 reagent (Dojindo Laboratories, Japan) mixed with 90 μL of complete medium was added to each well and incubated at 37 °C for 2 h, then the absorbance was measured at 450 nm. For colony formation assays, cells were cultured in six-well plates at a concentration of 1,000 cells per well at 37 °C with 5% CO_2_ for 10–14 days. Cells were fixed with paraformaldehyde and stained with crystal violet, and then the number of colonies was counted. EdU assays were performed using the EdU assay kit (Beyotime, China). Cells were incubated with 10 μM EdU for 2 h, then fixed with paraformaldehyde and permeabilized with Triton X-100. Incubating cells with Click Reaction Solution for half an hour and using Hoechst for nuclear staining. Finally, observed and photographed with a fluorescence microscope.

### Transwell and wound healing assays

Transwell chambers (Corning, USA) were utilized for Transwell assays. A total of 200 μL of serum-free medium containing 5 × 10^4^ suspended cells was added to the upper chamber, while 500 μL of medium supplemented with 20% FBS was placed in the lower chamber. Following a 24-hour incubation, the cells were fixed with 4% paraformaldehyde and subsequently stained with crystal violet. For wound healing assays, when the cells were plated in six-well plates and reached approximately 90% confluence, a sterile pipette tip was employed to create artificial gaps. Images at the time point zero hours were captured. After an additional incubation in serum-free medium for another 24 hours, images of the same location were recorded again to assess gap closure.

### Cell viability and MDA assays

ESCC cells were plated in 96-well plates at a density of 5,000 cells per well and subsequently treated with RSL3, Fer-1, Z-VAD, or Necro-1 for cell viability assessments. Following the treatment, cell viability was quantified using the CCK-8 assay, with absorbance readings taken at 450 nm.

The levels of lipid peroxidation were quantified using the Lipid Peroxidation MDA Assay Kit (Beyotime, China). Cell samples were lysed with RIPA lysis buffer, and the supernatant was collected. The protein concentration was determined using the BCA Protein Assay Kit. Subsequently, the MDA detection working solution was mixed with each protein sample and incubated at 100 °C for 15 min. Following this, the samples were centrifuged, and the supernatant was transferred to a 96-well plate for absorbance measurement at 532 nm.

### Lipid ROS and Mitochondrial membrane potential assays

ESCC cells were treated with RSL3 or DMSO for 12 h. The cells were collected and suspended, then treated with 10 µM C-11 BODIPY (Invitrogen, USA) in the dark for 30 min at 37 °C. Fluorescence intensity was immediately analyzed by flow cytometry. Mitochondrial membrane potential was assessed using a JC-1-based assay kit (Abcam, UK) according to the manufacturer’s protocol. Changes were quantified by the fluorescence shift from JC-1 aggregates (red fluorescence) to monomers (green fluorescence) using fluorescence microscopy.

### CUT&Tag-seq and CUT&Tag-qPCR assays

Sequencing libraries were constructed using the Hyperactive Universal CUT&Tag Assay kit (Vazyme, TD904) following the manufacturer’s standard protocol. Amplified libraries were determined and sequenced by Novogene Co., Ltd (Beijing, China). For data processing, FASTQC was used to measure the data quality distribution, and the bwa program was employed to align the clean reads to reference genome sequences. The MACS2 software was used with a *q* < 0.05 for calling peaks and screening. Peaks with fold change >1.5 and *p* < 0.05 were defined as statistically significant. IGV was used to visualize genome-wide normalized signal coverage tracks. CUT&Tag-qPCR experiments were conducted using the Hyperactive Universal CUT&Tag Assay Kit (Vazyme, TD904), and experiments were conducted according to the instructions. The qPCR primers were shown in Supplementary Table S1, and the DNA spike-in was used as a reference.

### NADH level measurement

For ESCC cells, 1 × 10^6^ cells per sample were collected, and intracellular NADH levels were determined using an NAD + /NADH assay kit (MedChemExpress, USA) according to the manufacturer’s instructions. Briefly, cells were collected and lysed with 200 μL of NADH extraction buffer for 10 min, followed by centrifugation to collect the supernatant. Standard solutions were prepared according to the manufacturer’s instructions to generate standard curves, and working solutions were prepared for detection. Samples or standards were mixed with the working solution and incubated at 37 °C in the dark for 10 min. Subsequently, chromogenic reagent was added, and the mixture was incubated at 37 °C in the dark for 20 min. Absorbance was measured at 450 nm.

### CoQ10 and CoQ10H_2_ analysis

The cell pellet was resuspended in 0.3 mL of acetone (containing 1% butylated hydroxytoluene, BHT), vortexed for 1 min, and subjected to ultrasonic treatment at 4 °C for 5 min, followed by low-temperature extraction at 4 °C for 30 min. After centrifugation at 12,000 rpm for 15 min at 4 °C, the entire supernatant was collected and dried under a nitrogen stream. The residue was reconstituted with 0.1 mL ethyl acetate and 0.1 mL methanol, vortexed for 1 min, and filtered through a 0.22 μm membrane. Analyses were performed using a Waters UPLC system coupled with an AB SCIEX 4000 MS/MS. Chromatographic separation was achieved on a Waters HSS T3 column (2.1 × 100 mm, 1.7 μm) with mobile phase A (0.1% formic acid in water) and B (methanol) under isocratic elution (100% B, 0.8 mL/min, 5 min). The column temperature was maintained at 40 °C, and the sample tray was set to 10 °C. Mass spectrometric detection was conducted in APCI positive ion mode with the following parameters: ion spray voltage 5500 V, source temperature 450 °C, curtain gas 35 psi, collision gas 7 psi, and ion source gases 1/2 at 40/50 psi. Target analytes were monitored in multiple reaction monitoring mode. Standard solutions were analyzed under identical conditions to determine retention times and construct calibration curves. The concentration of target analytes in samples was quantified by fitting peak areas to the standard curve, corrected for dilution factors. Quality control samples were used to validate system stability and reproducibility.

### Xenograft model

All animal experiments were approved by the Animal Experimentation Ethics Committee of Zhengzhou University. Female BALB/c nude mice aged 4 weeks were purchased from GemPharmatech (Jiangsu, China) and housed in a pathogen-free environment. 3 × 10^6^ SETD7 knockdown cells or overexpressed cells were injected subcutaneously into mice, respectively, and control cells. The volume of the tumor was recorded every 4 days. After 20 days, mice were euthanized, and the weight and volume of the tumor were measured. All procedures were approved by the Biomedical Ethics Committee of the First Affiliated Hospital of Zhengzhou University.

### Statistical analysis

All experiments were repeated at least three times. Statistical analysis was performed using GraphPad Prism Software. Data are presented as mean ± SD. Independent t-tests, one-way analysis, and two-way analysis of variance were used to compare quantitative data between groups, and the Chi-square test was used to analyze categorical data. Statistical significance was considered to exist when *p* < 0.05.

## Supplementary information

Original data file of western blot

Supplementary figures

Supplementary tables

## Data Availability

Data will be made available on request.
