# Supplementary material for: SETD7-mediated H3K4me1 activates ALDH1A3 to drive ferroptosis resistance in esophageal squamous cell carcinoma
Source: Cell Death Dis. 2025 Nov 7;16(1):810. doi: 10.1038/s41419-025-08133-7 (PMC12595051; doi:10.1038/s41419-025-08133-7)
Supplement: Supplementary file 1 — Original data file of western blot [file 41419_2025_8133_MOESM1_ESM.docx]

**Fig. 1 F: SETD7 Left Fig. 1 F: SETD7 Right**

**
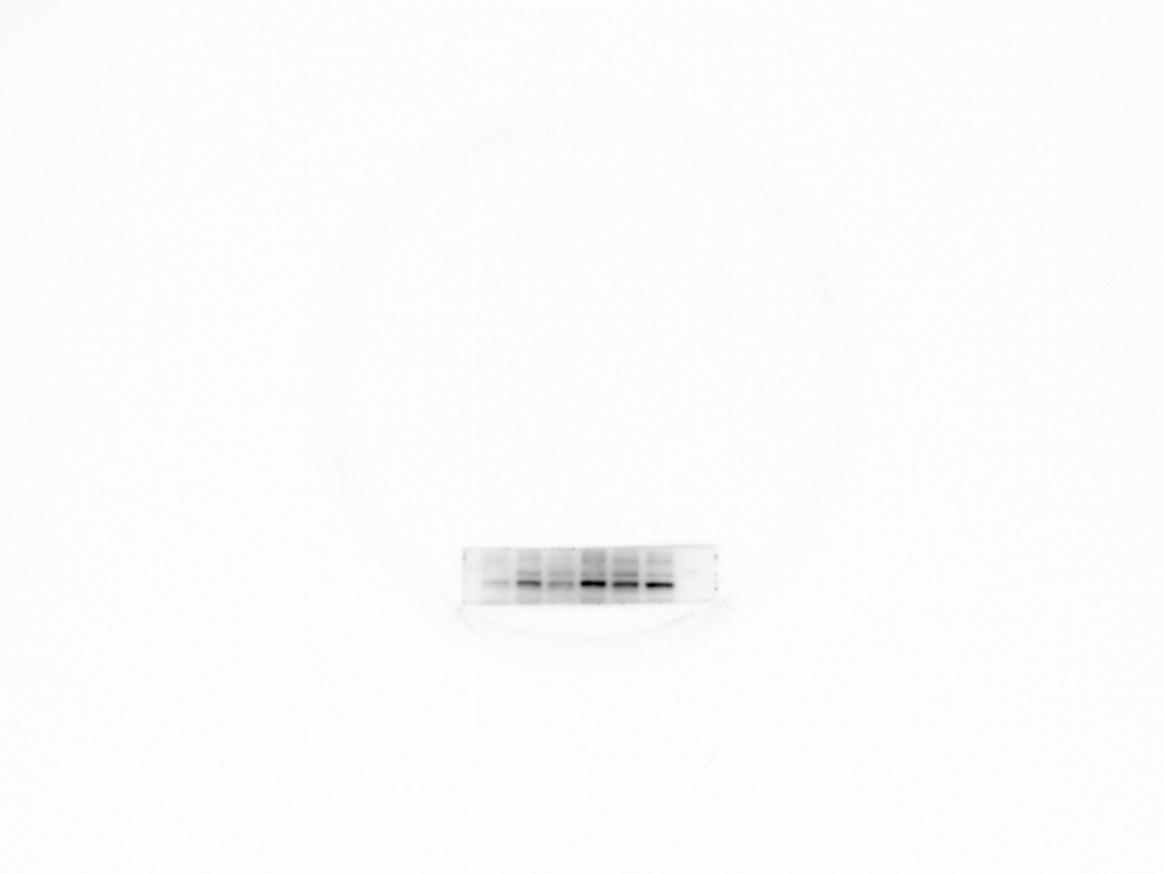
**
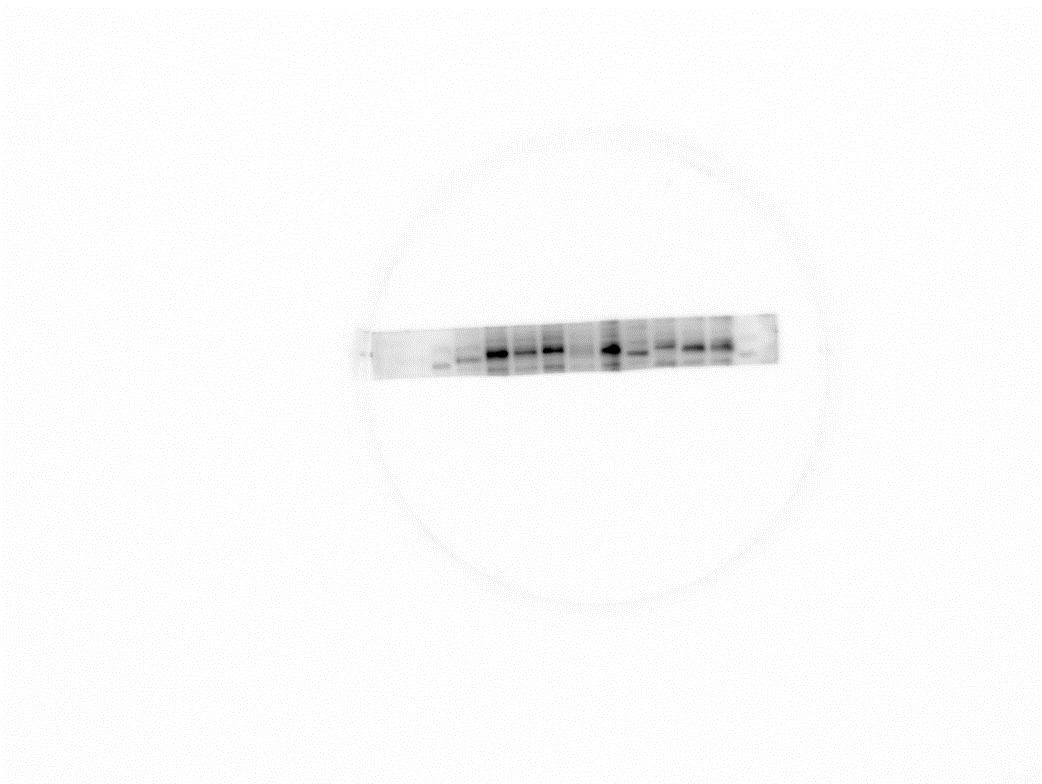


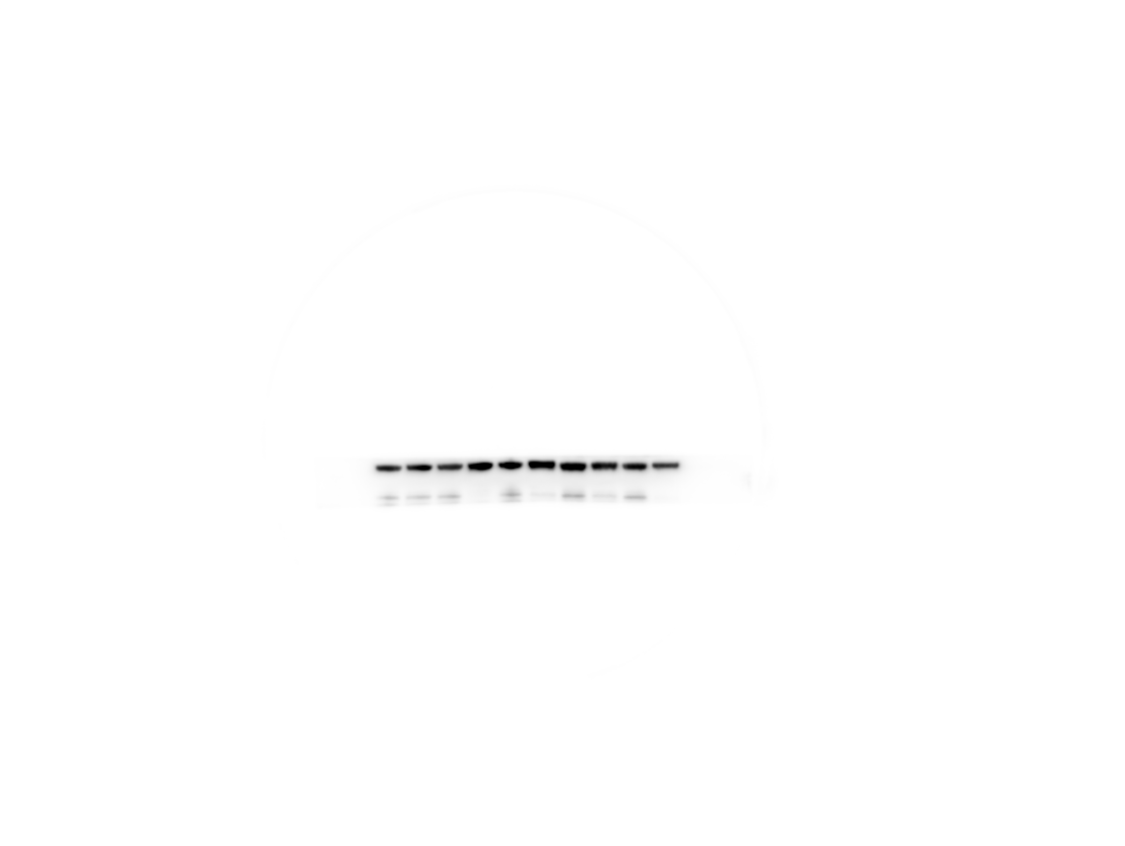
**
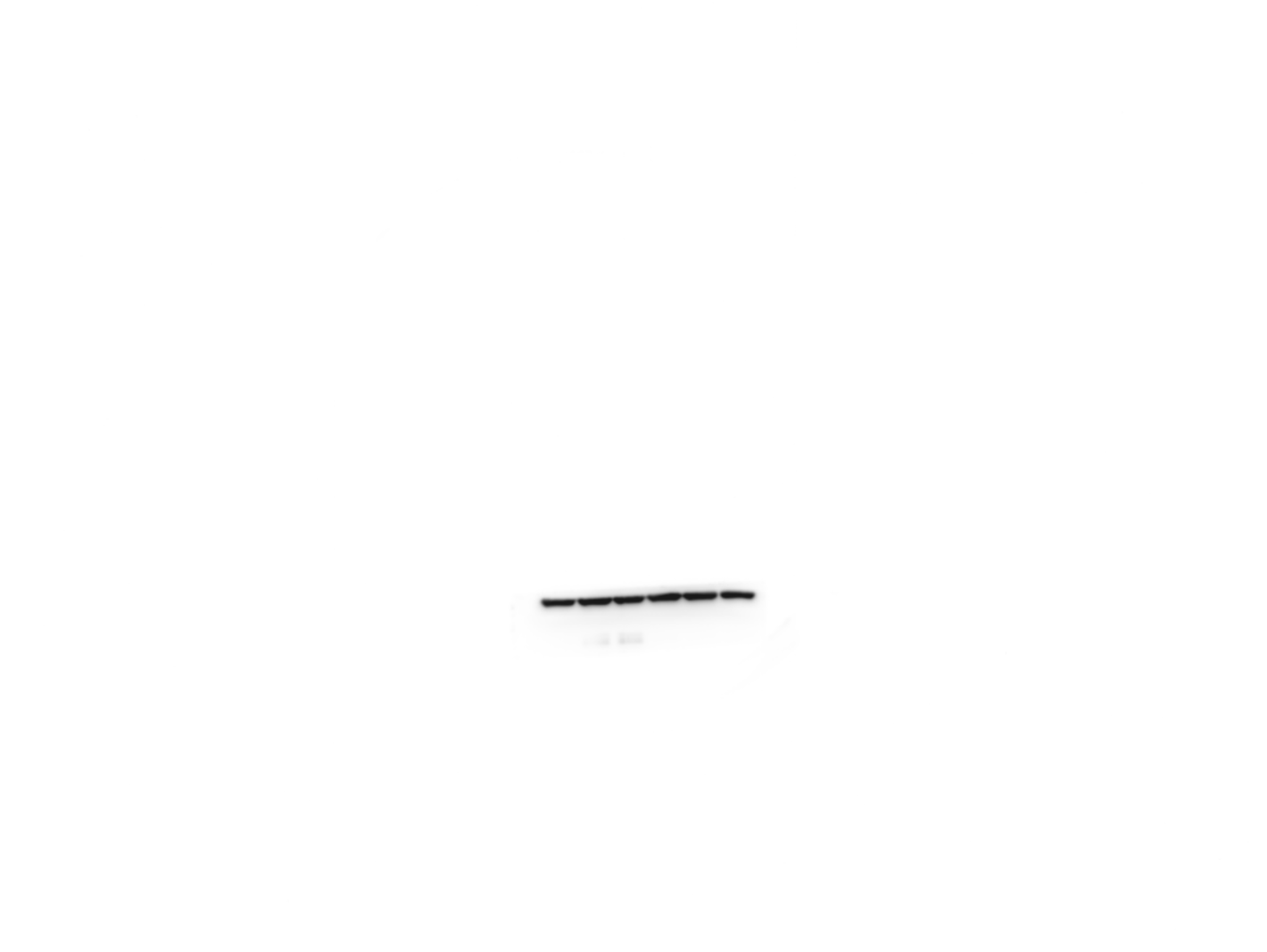
Fig. 1 F: ACTIN Left Fig. 1 F: ACTIN Right**

**Fig. 1 G: SETD7 Fig. 1 G: ACTIN**


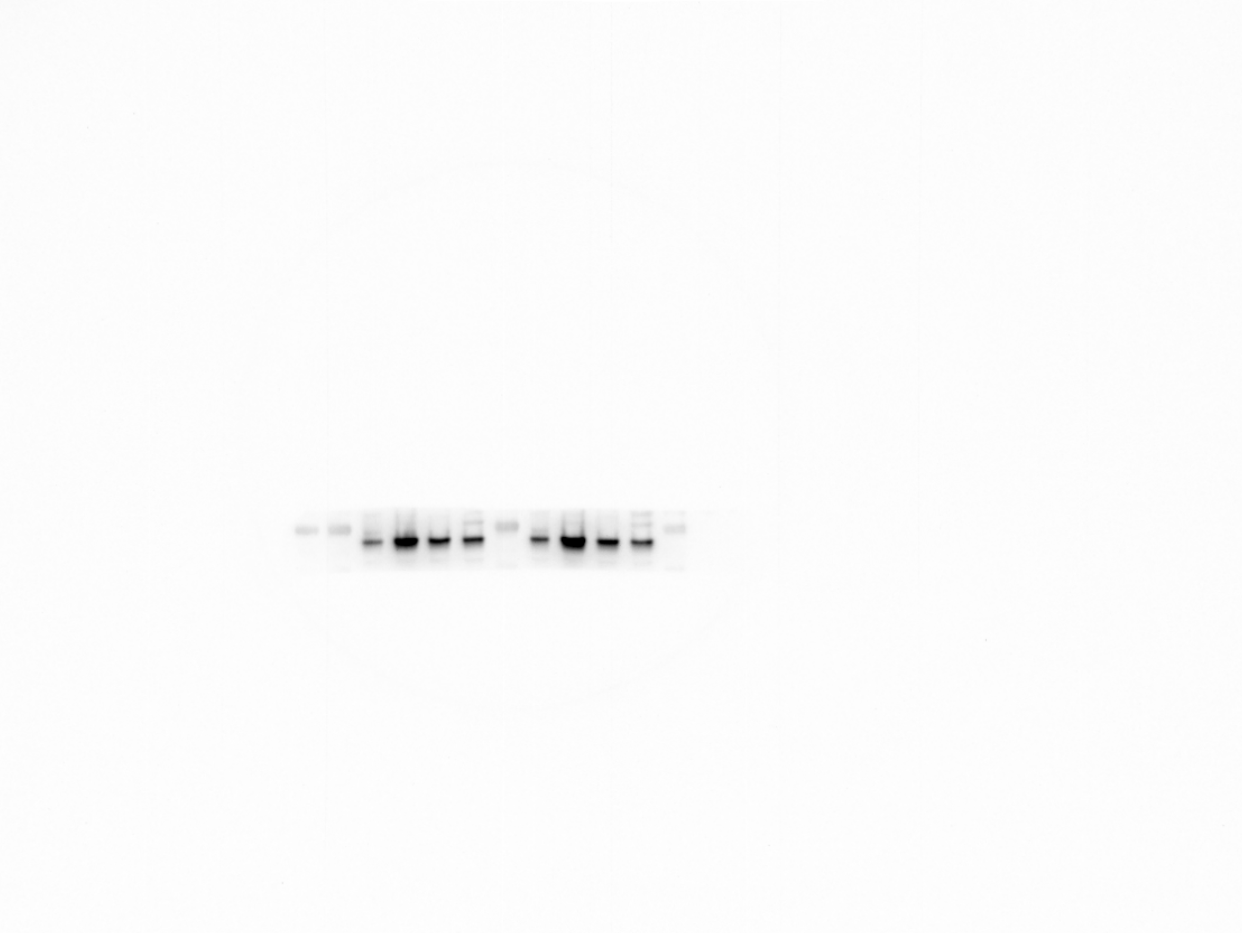

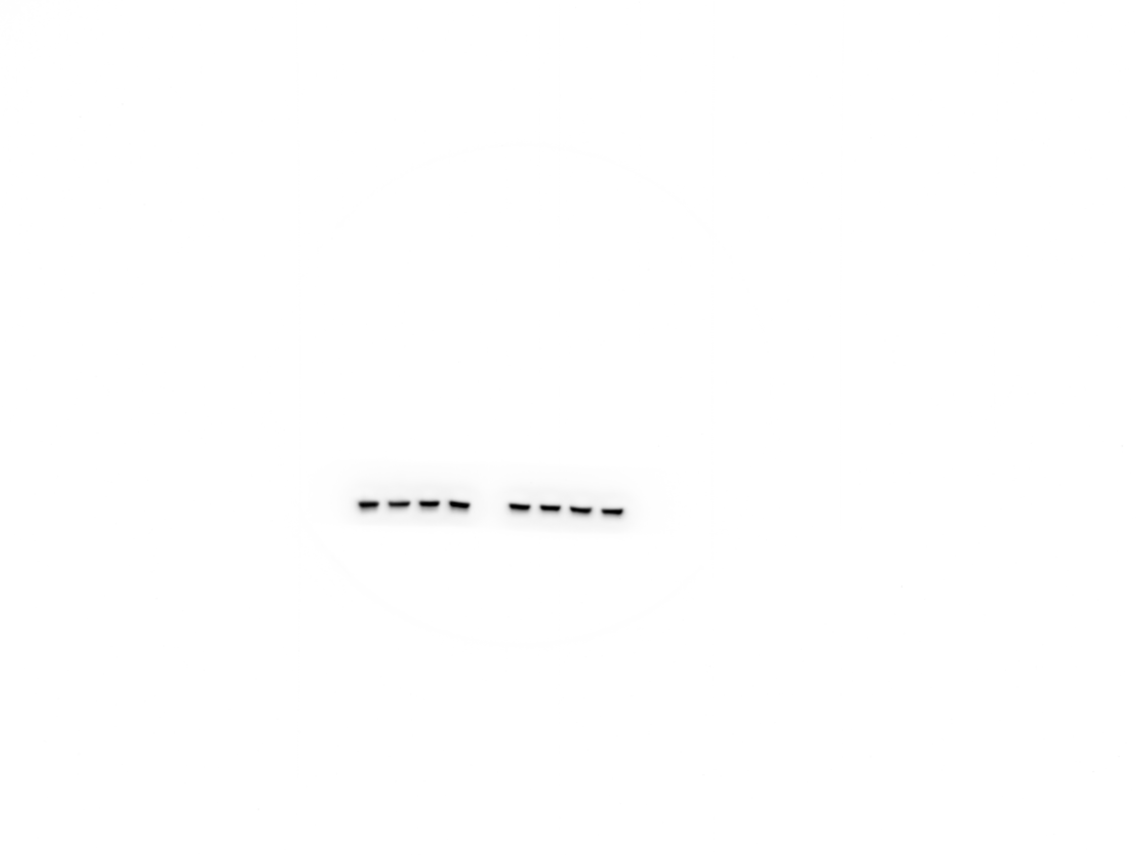


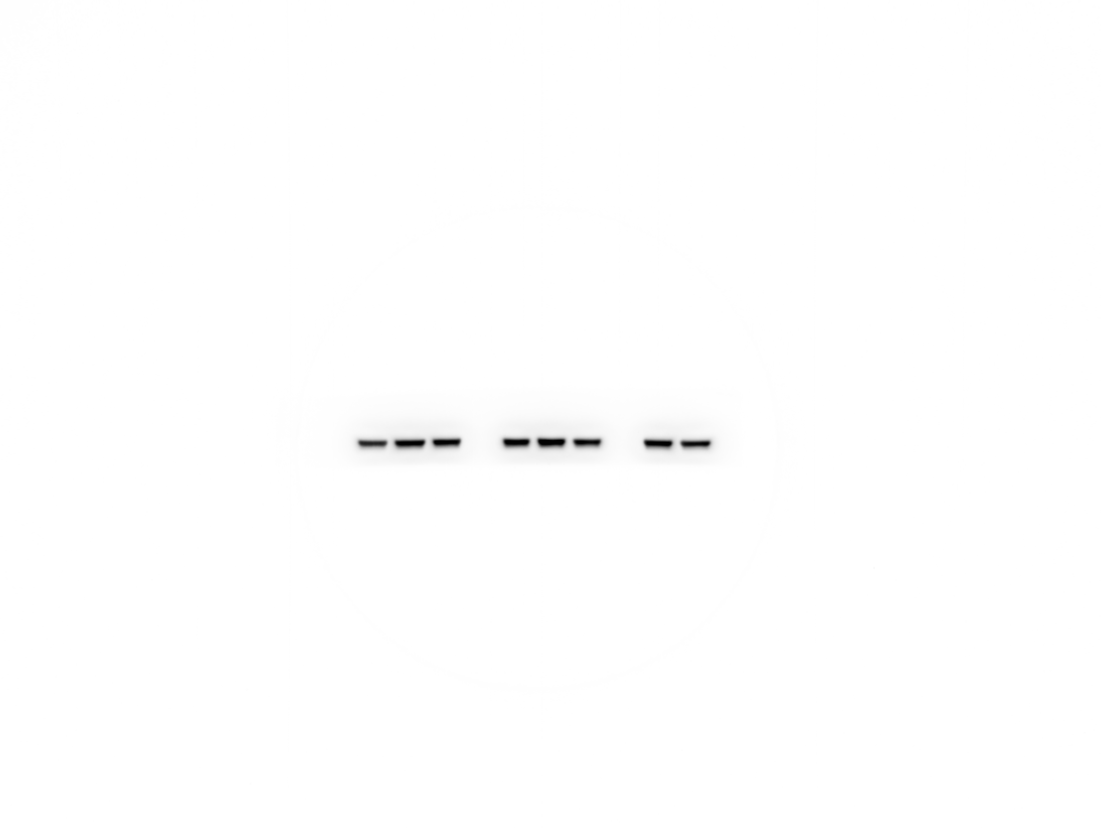

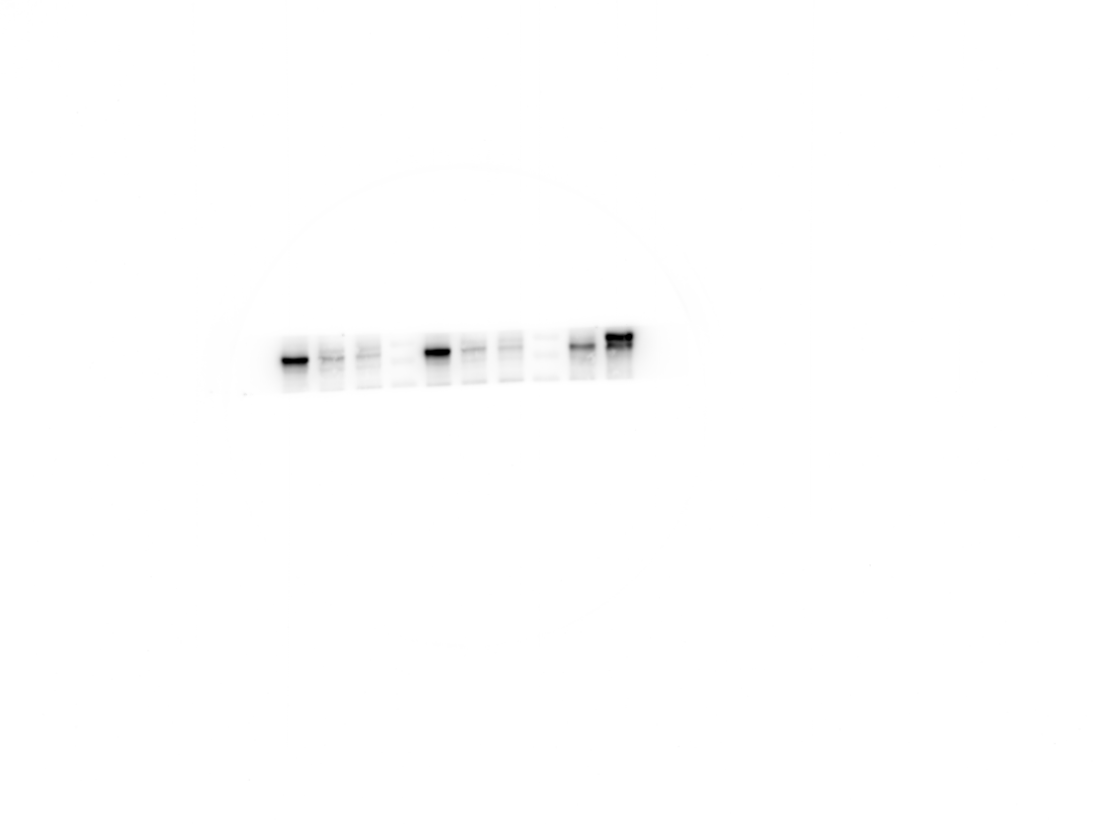
**Fig. 2 B: SETD7 Fig. 2 B: ACTIN**

Eca109

KYSE30

KYSE150

KYSE150

KYSE30

Eca109

**Fig. 4 A: SETD7(Eca109) Fig. 4 A: H3K4me1(Eca109) Fig. 4 A: H3(Eca109)**

**
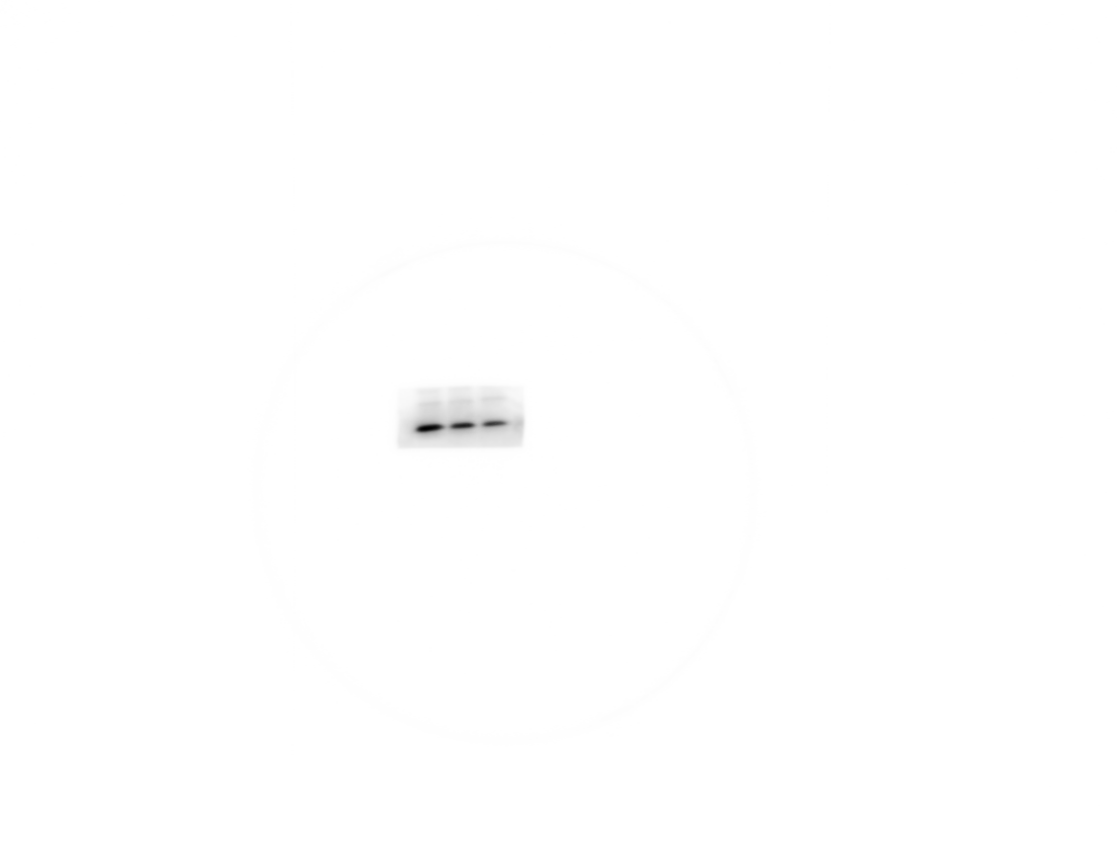

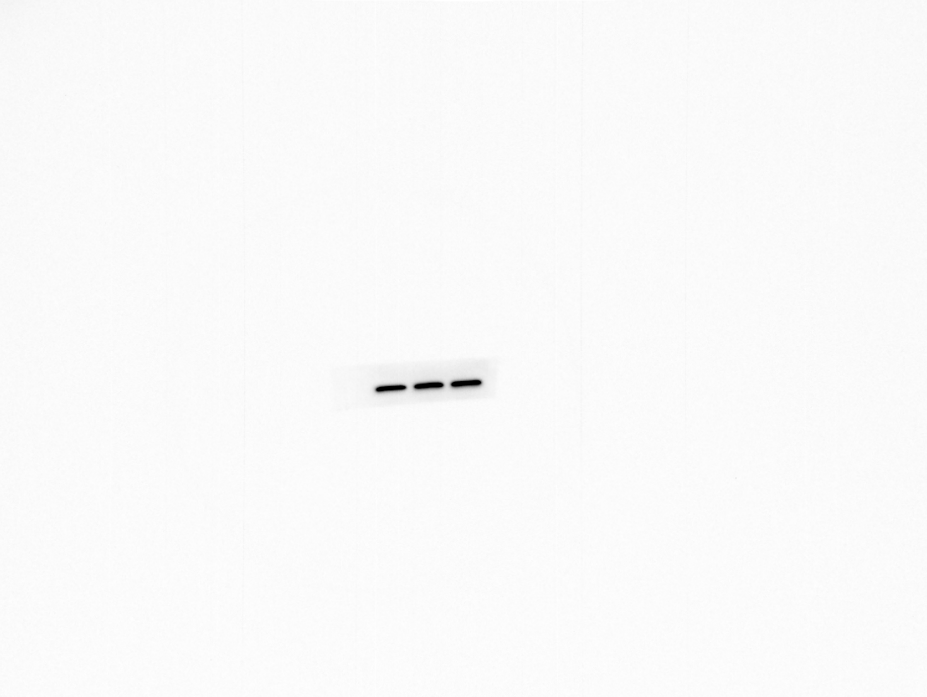

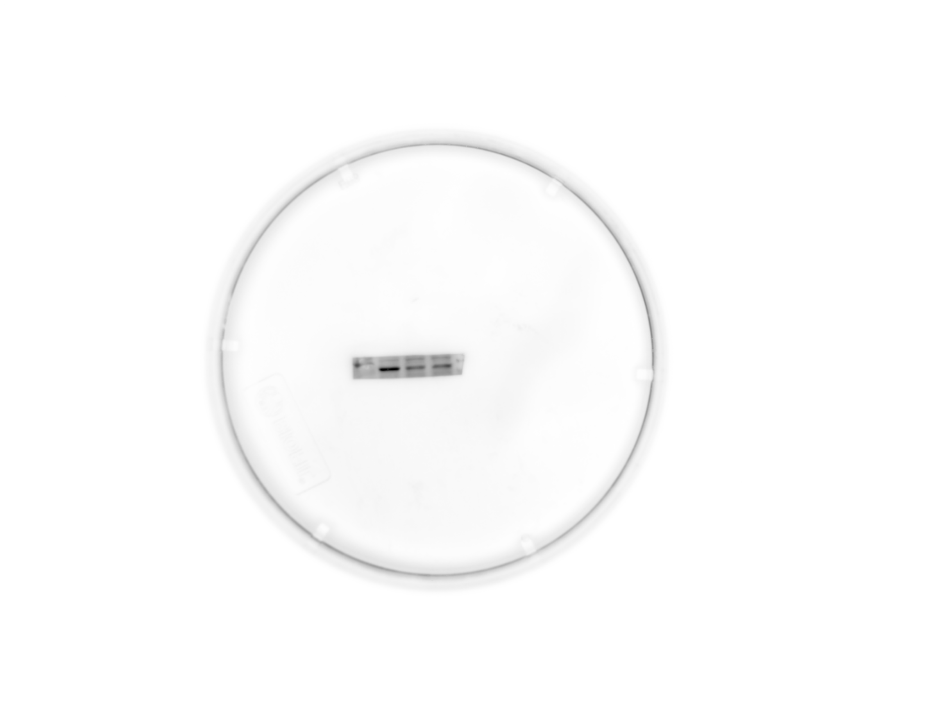
**

**Fig. 4 A: SETD7(KYSE30) Fig. 4 A: H3K4me1(KYSE30) Fig. 4 A: H3(KYSE30)**


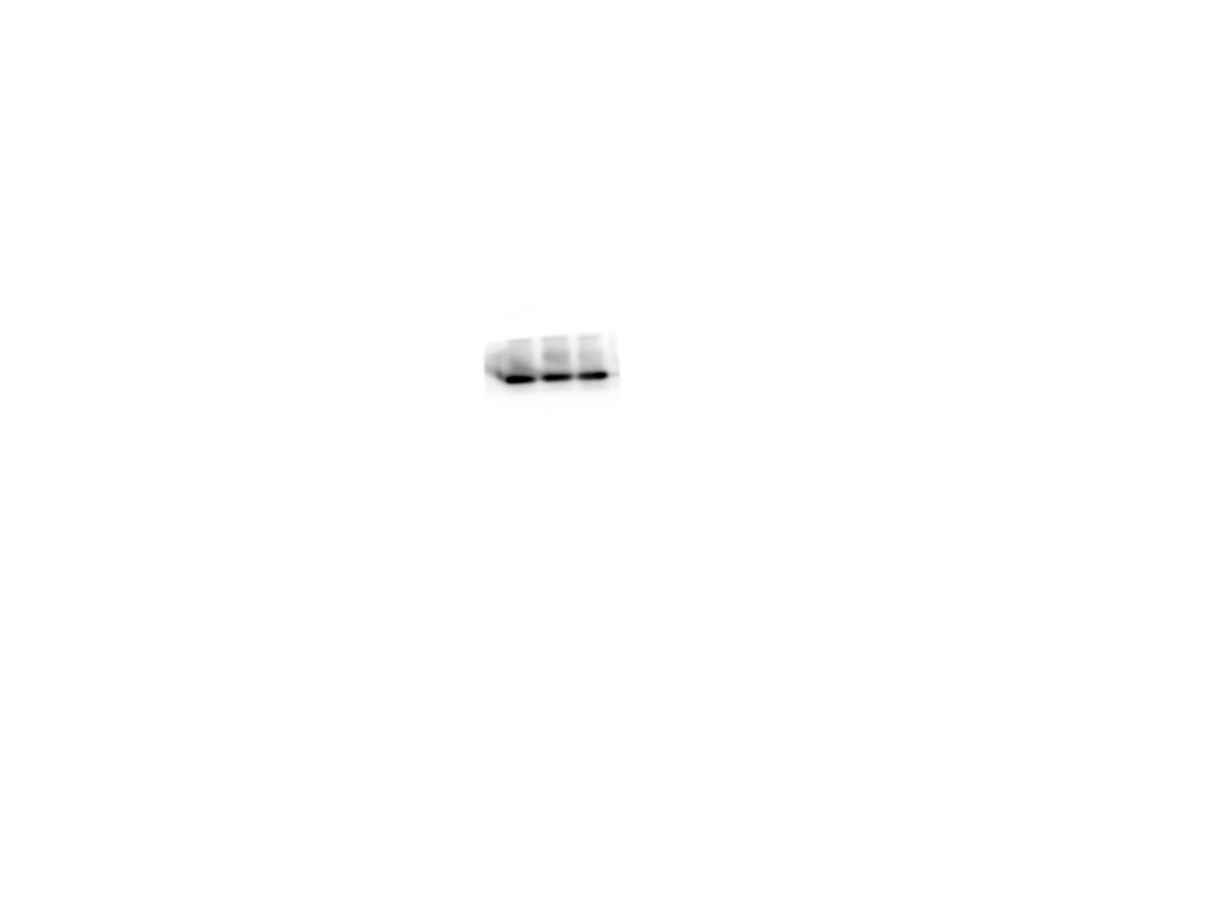

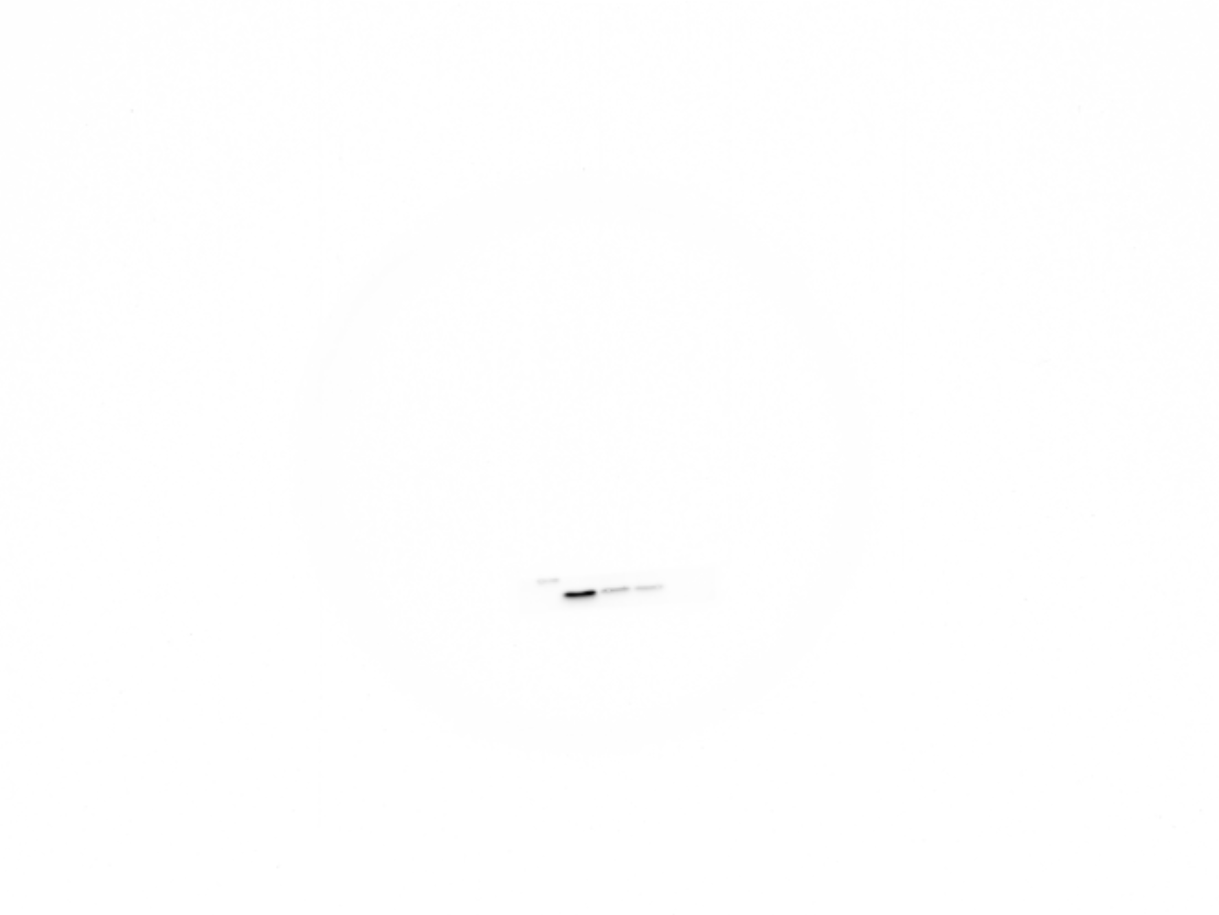


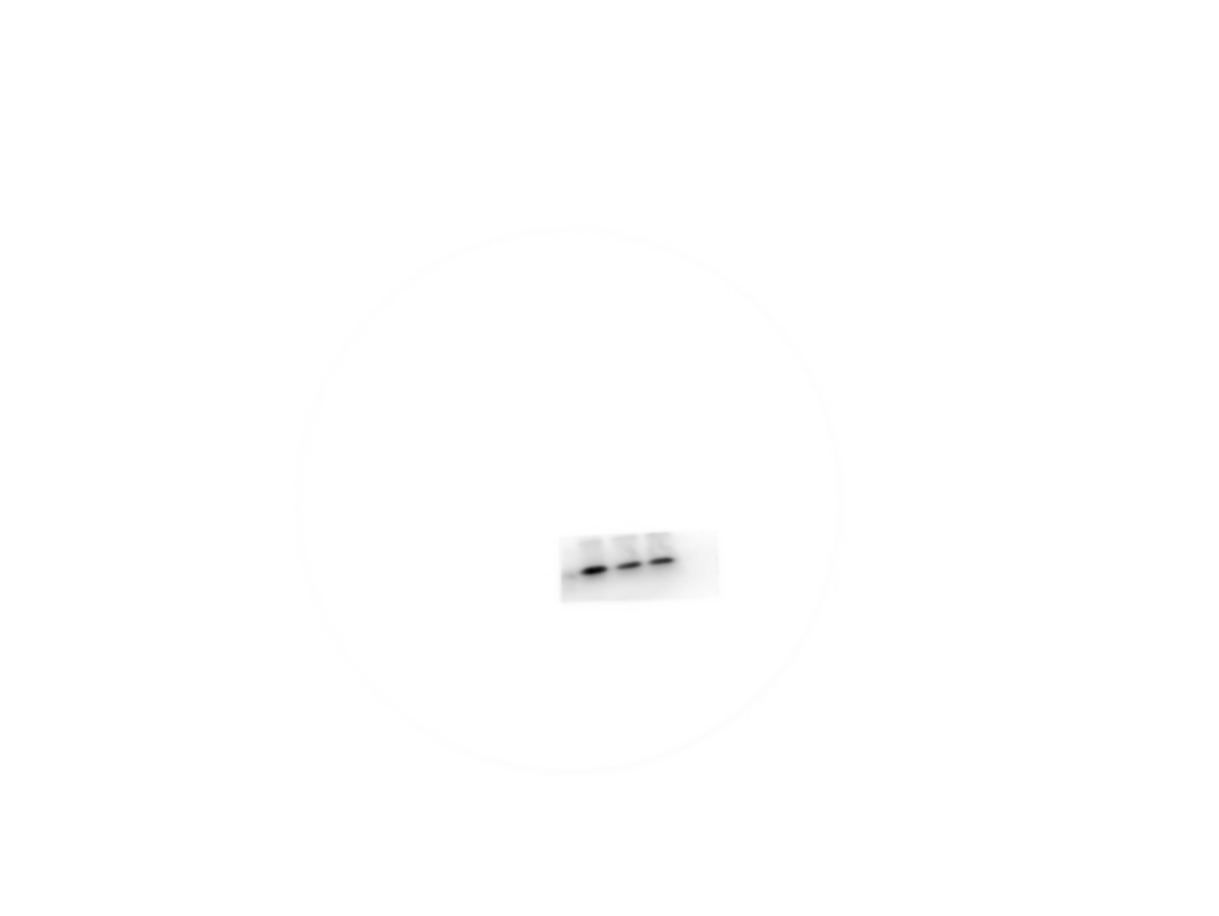


**Fig. 4 A: SETD7(KYSE150) Fig. 4 A: H3K4me1(KYSE150) Fig. 4 A: H3(KYSE150)**


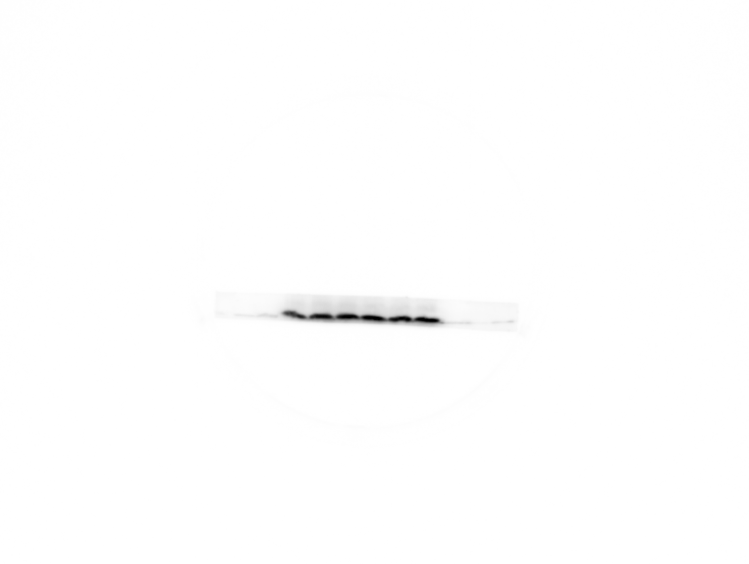

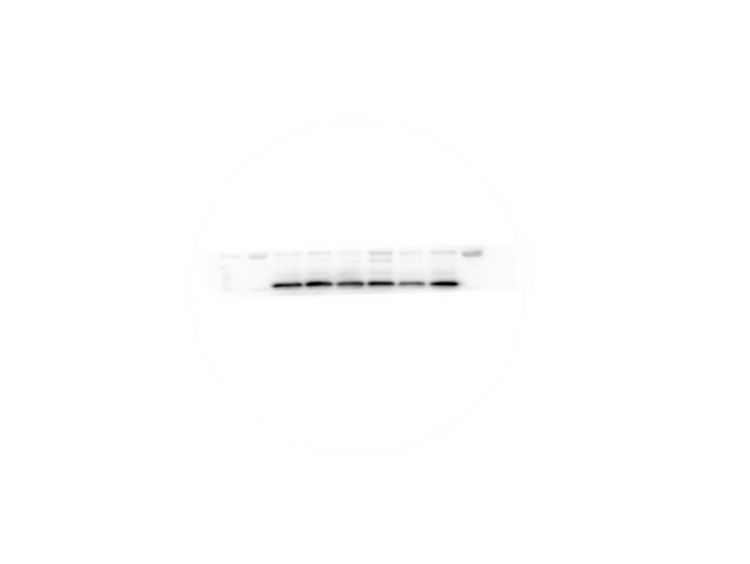
**
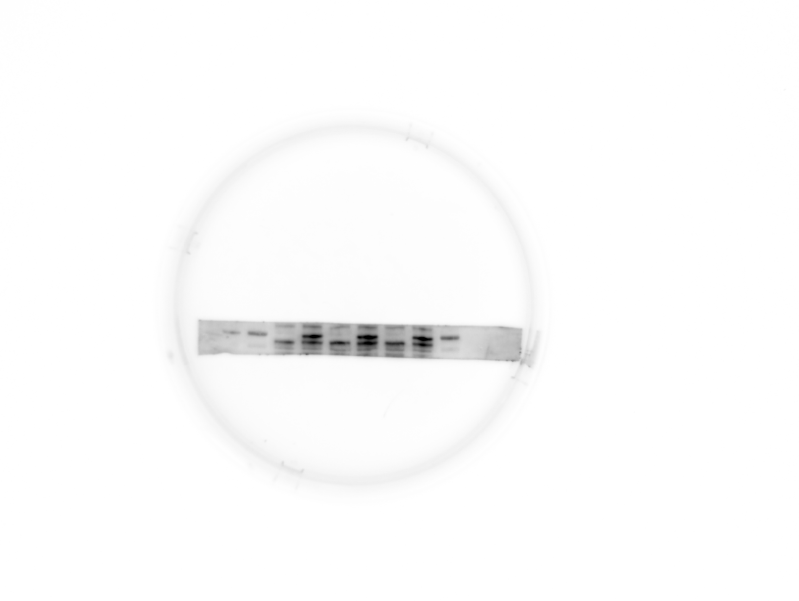
**

**Fig. 4 A: SETD7(KYSE510) Fig. 4 A: H3K4me1(KYSE510) Fig. 4 A: H3(KYSE510)**


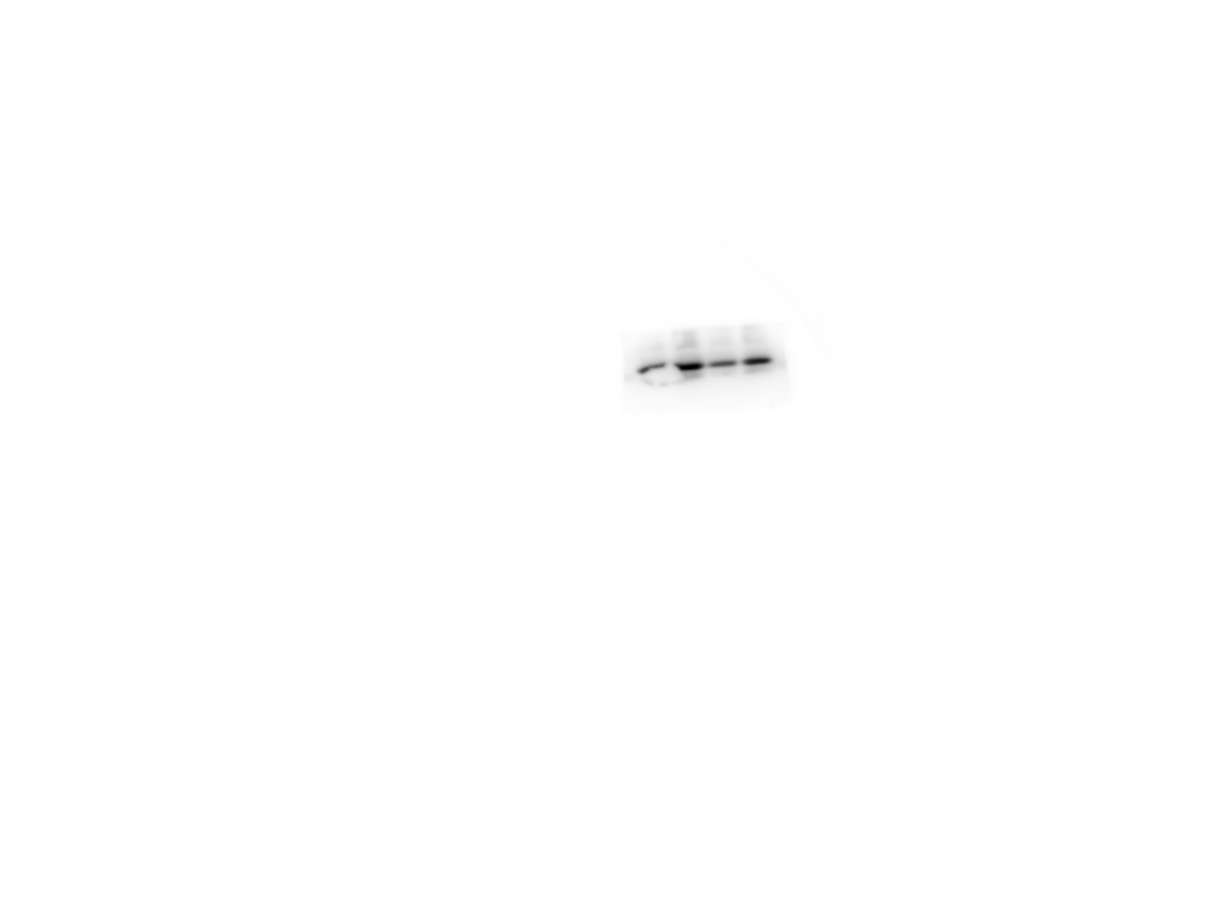

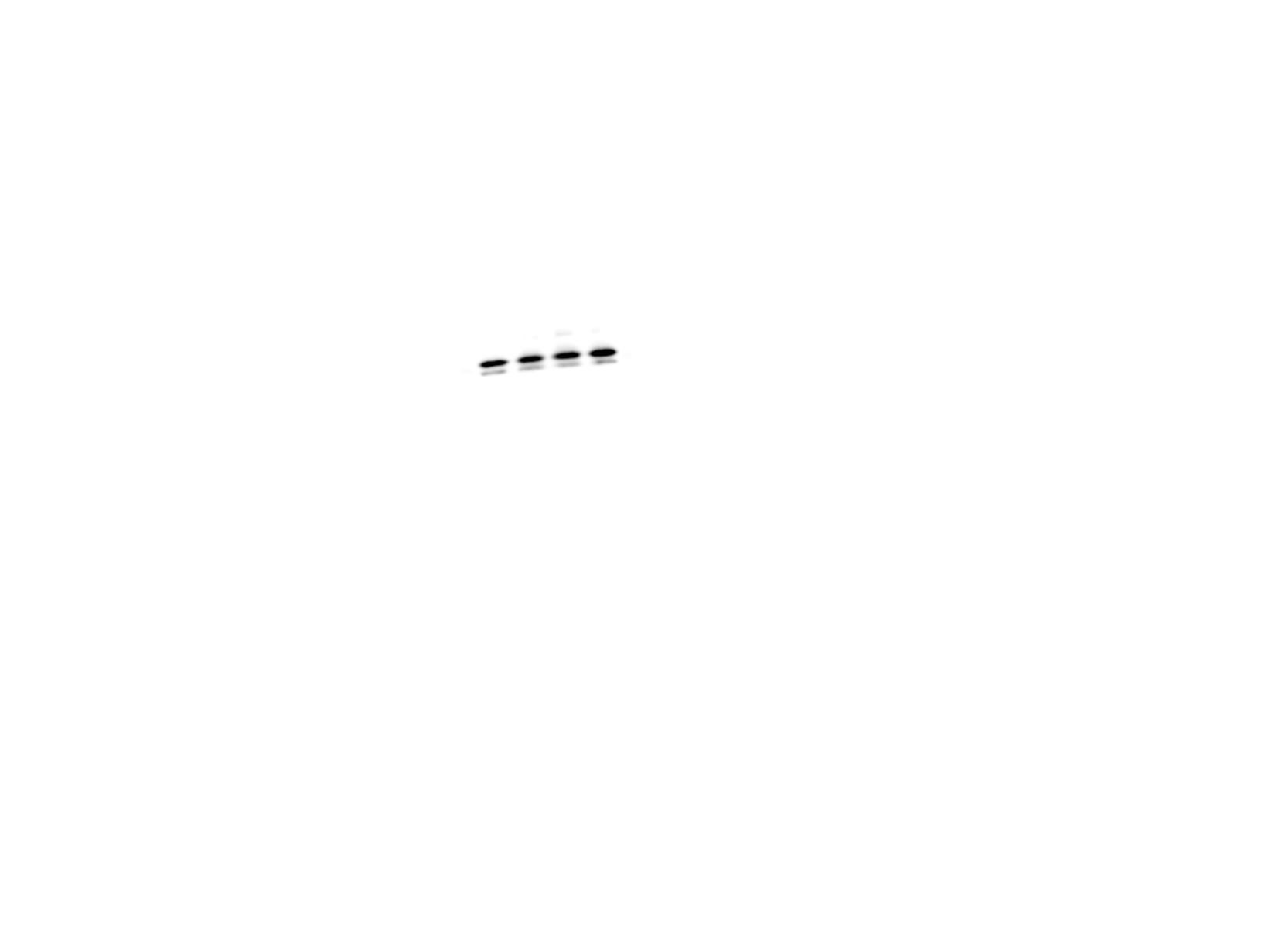

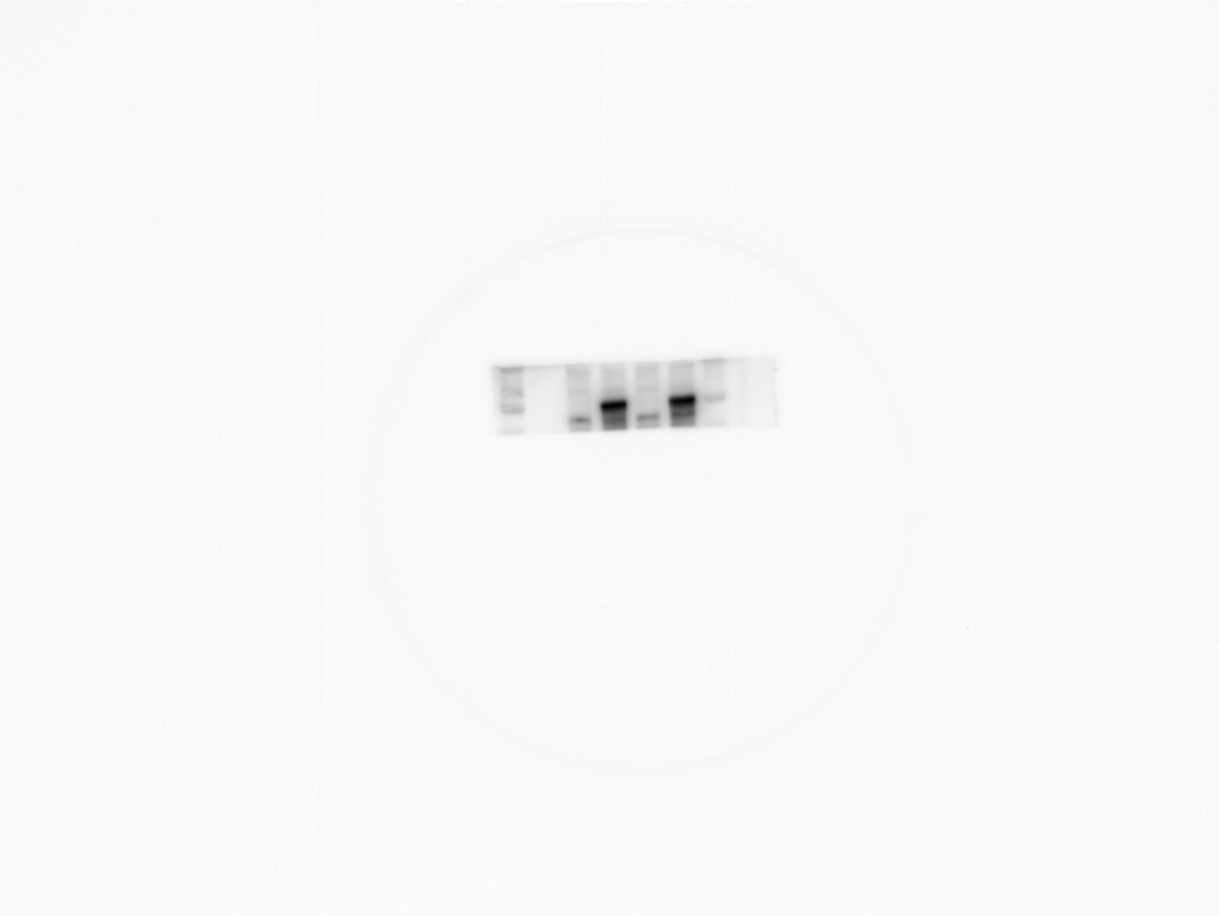


**Fig. 6 F: ALDH1A3 Fig. 6 F: SETD7 Fig. 6 F: ACTIN**


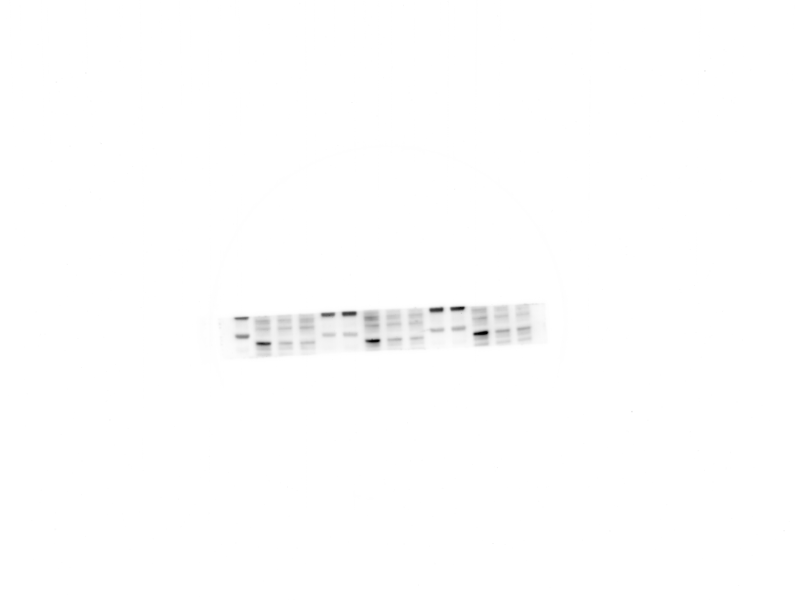

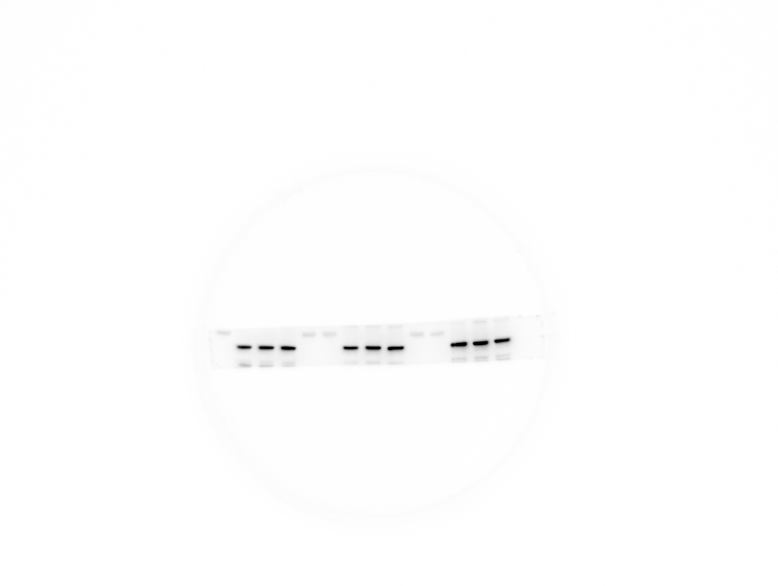

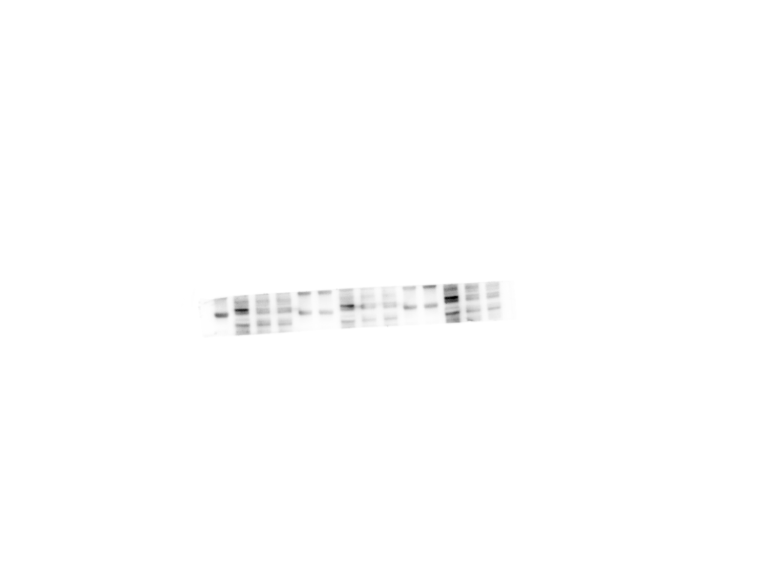


**Fig. 6 G: ALDH1A3 Fig. 6 G: SETD7 Fig. 6 G: ACTIN**


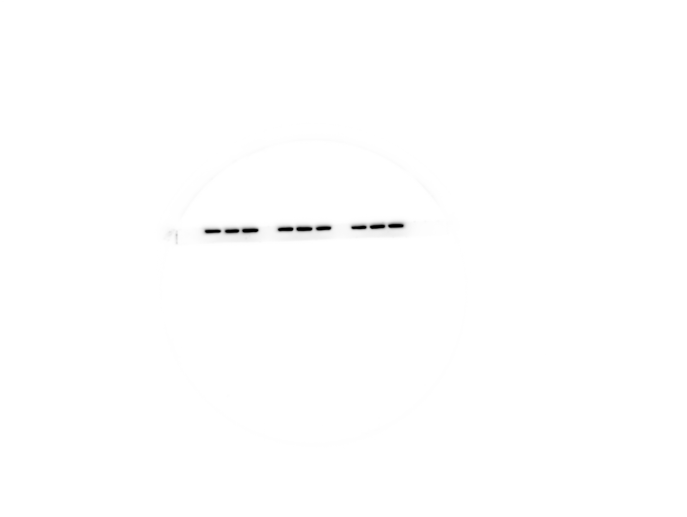

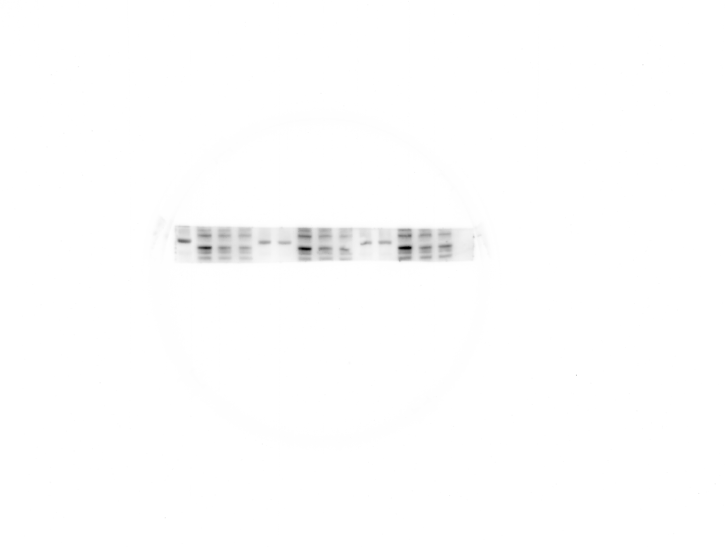
**
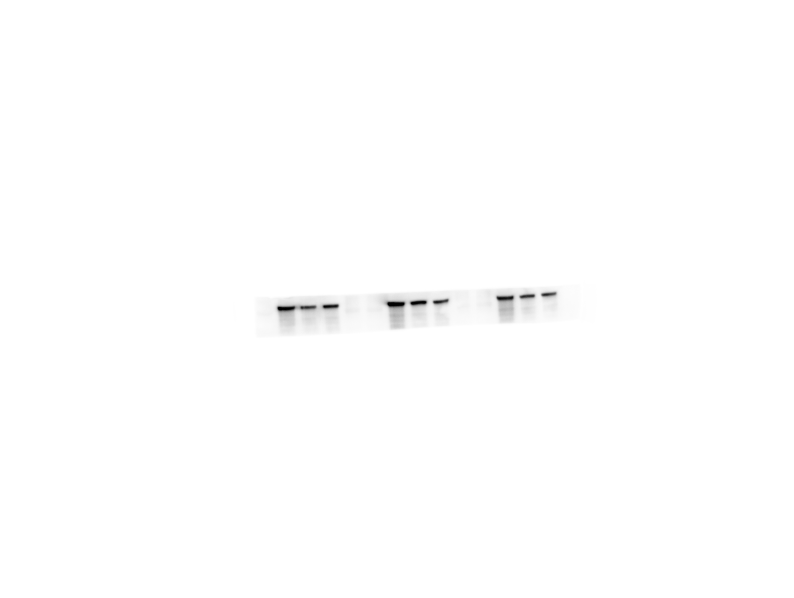
**

**Fig. 6 H: ALDH1A3 Fig. 6 H: SETD7 Fig. 6 H: ACTIN**

**
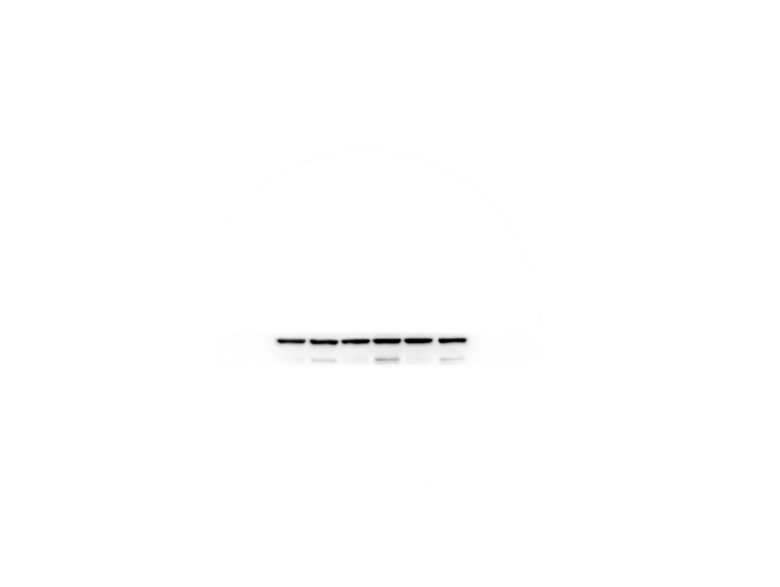
**
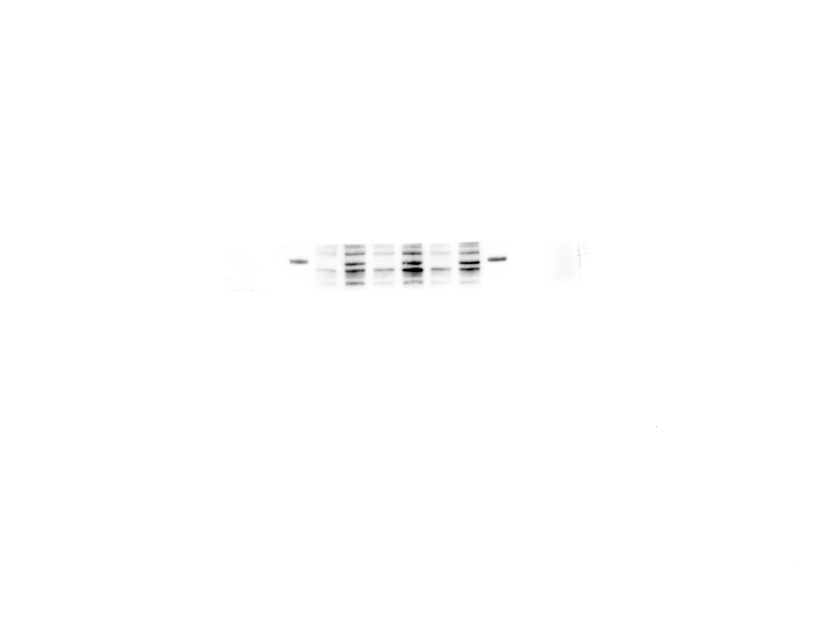

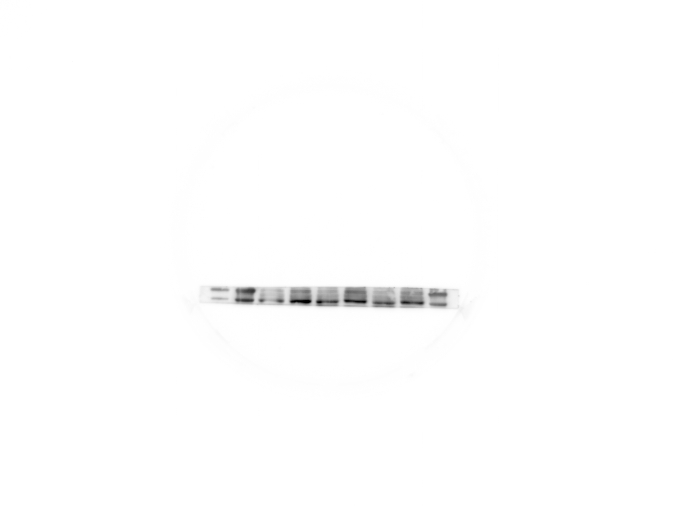


**
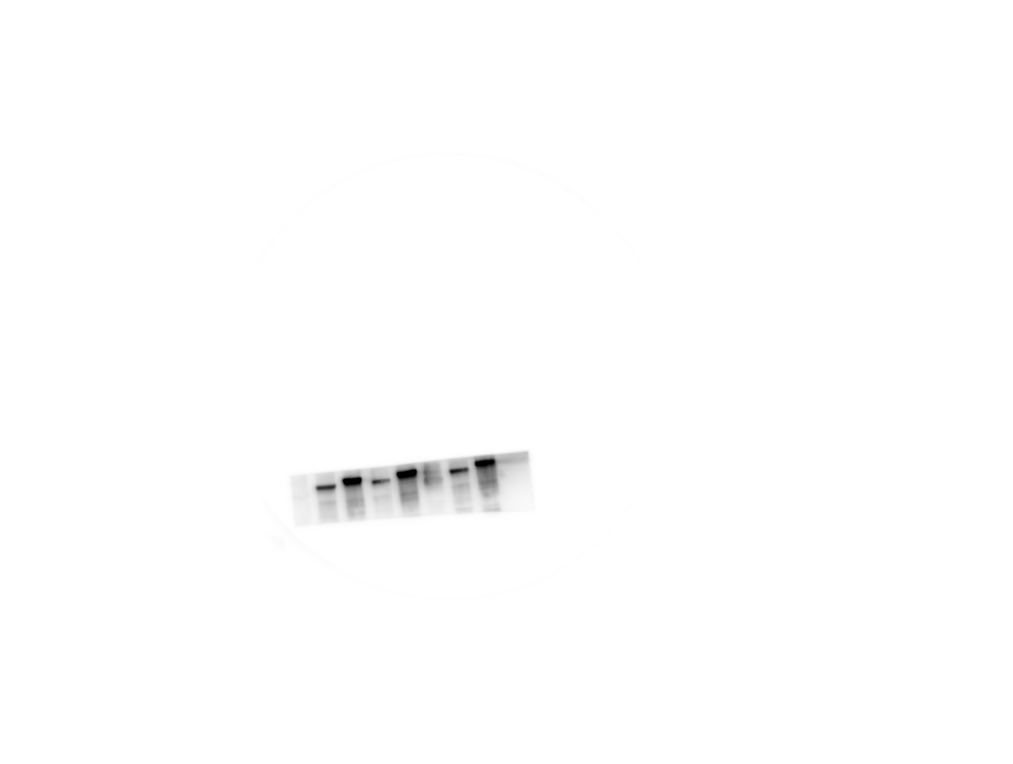

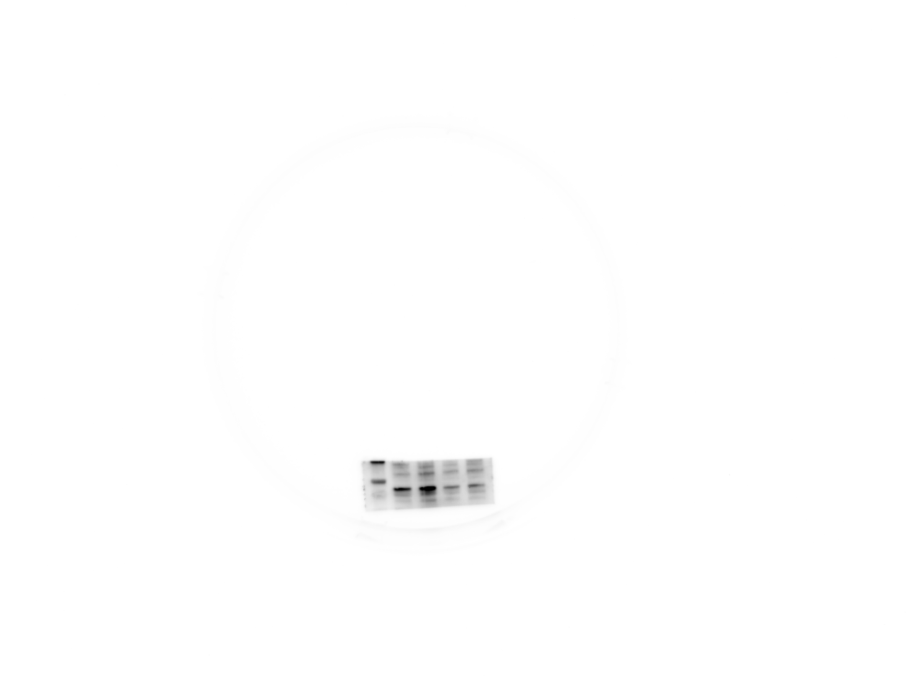

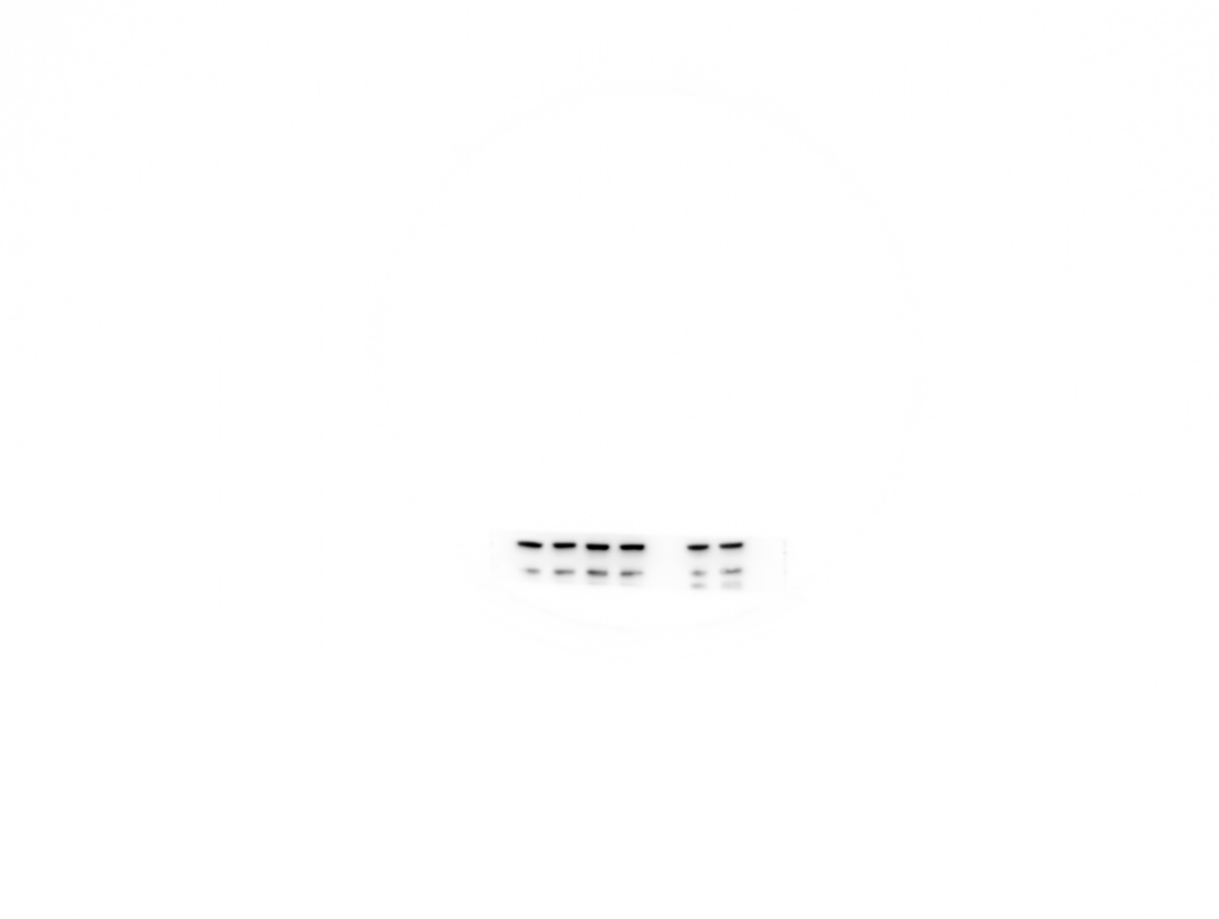
Fig. 7 C: ALDH1A3 Fig. 7 C: SETD7 Fig. 7 C: ACTIN**


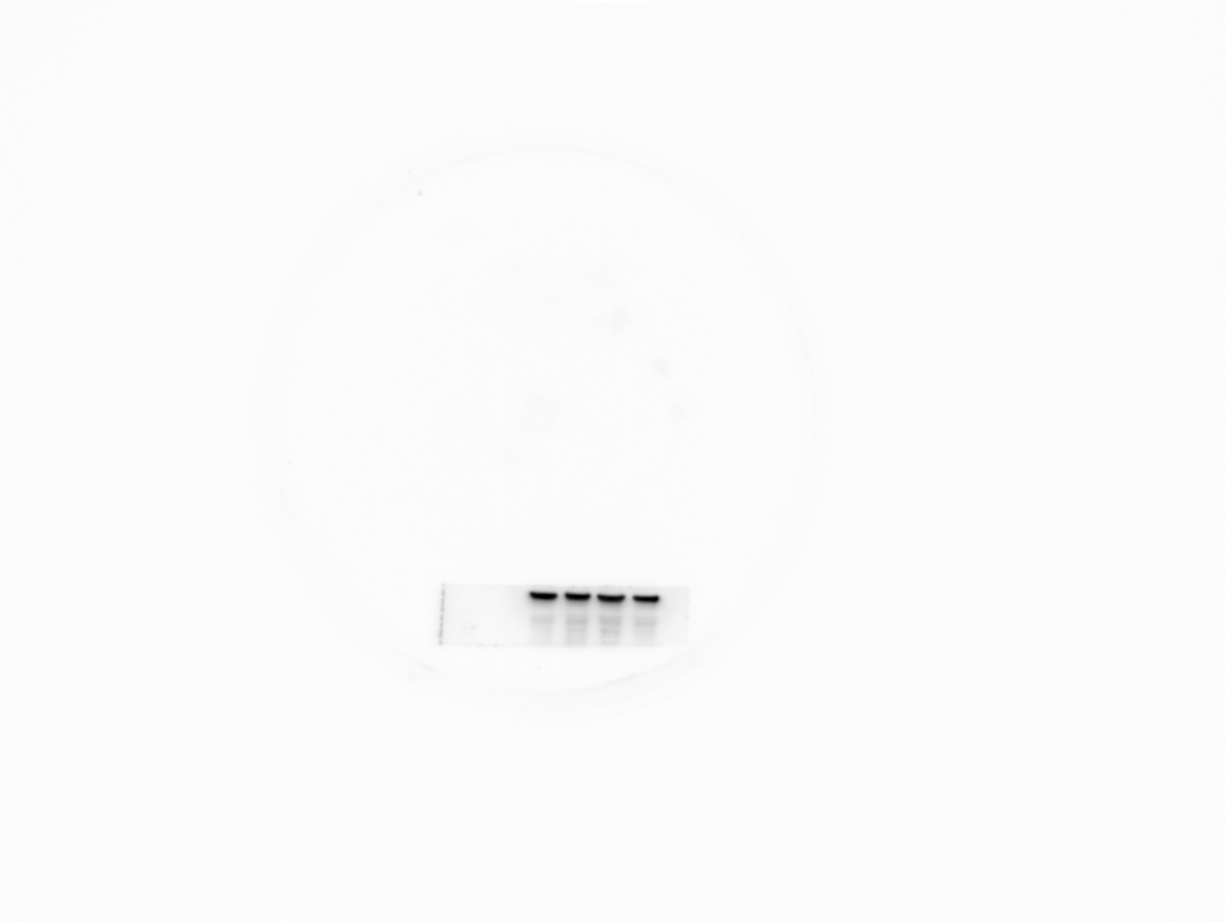

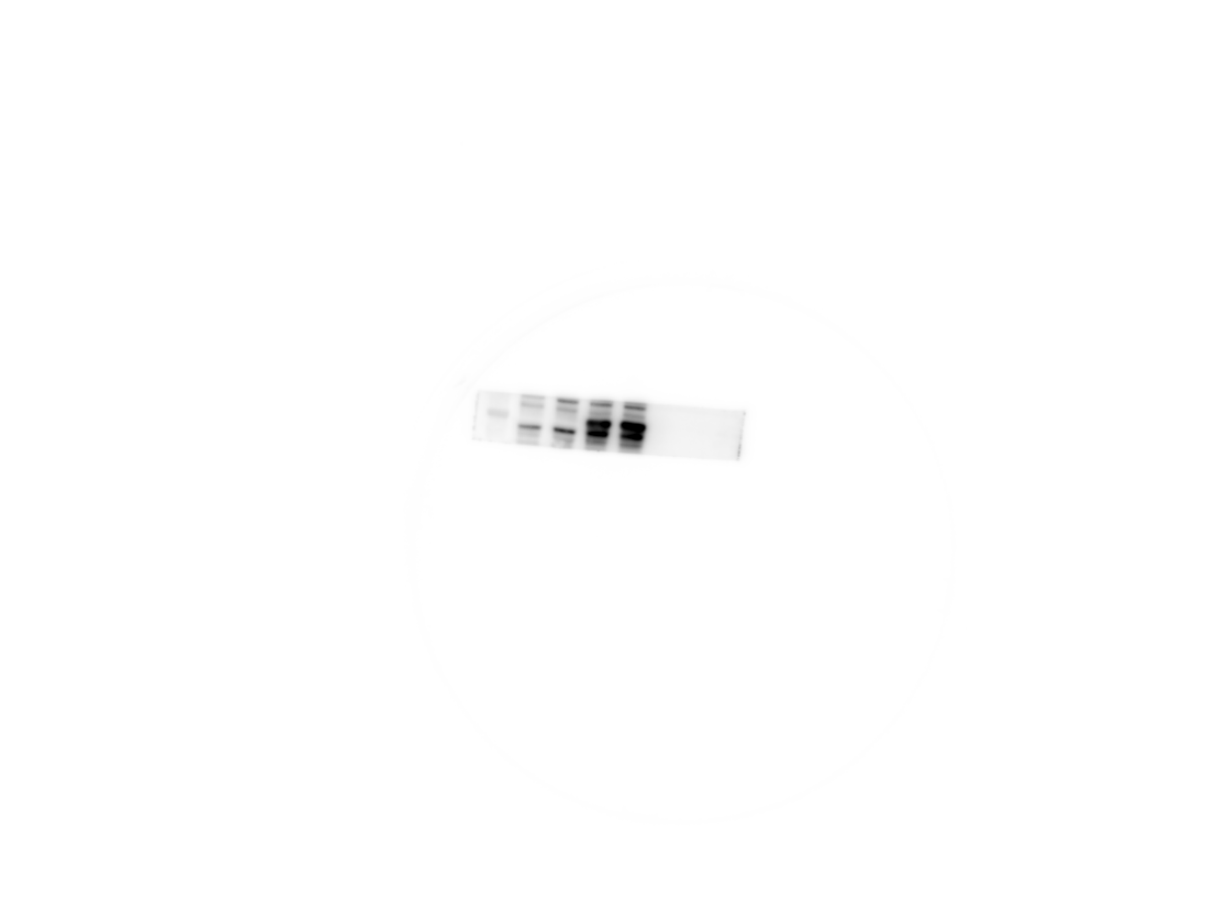

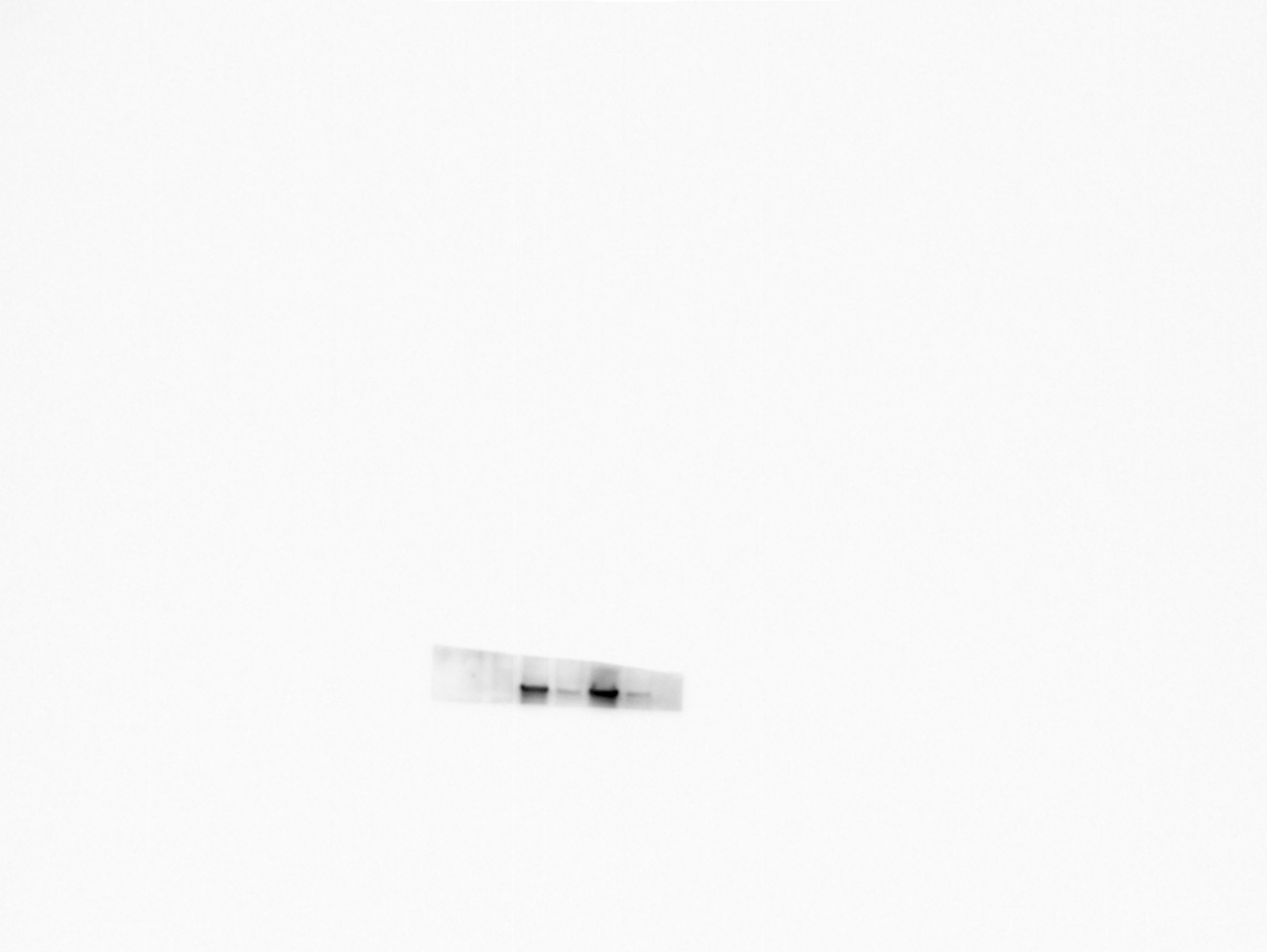
**Fig. 7 D: ALDH1A3 Fig. 7 D: SETD7 Fig. 7 D: ACTIN**

、

**Fig. 9 G: ALDH1A3(KYSE30) Fig. 9 G: SETD7(KYSE30) Fig. 9 G: ACTIN(KYSE30)**


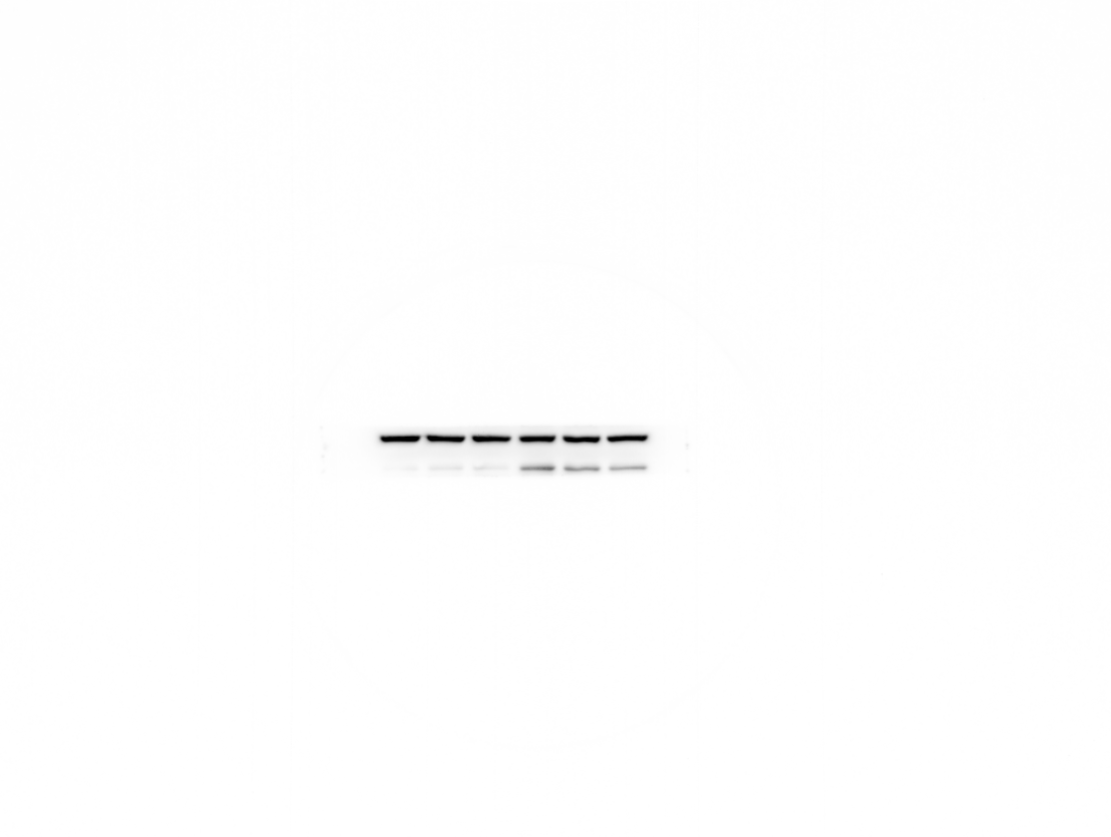

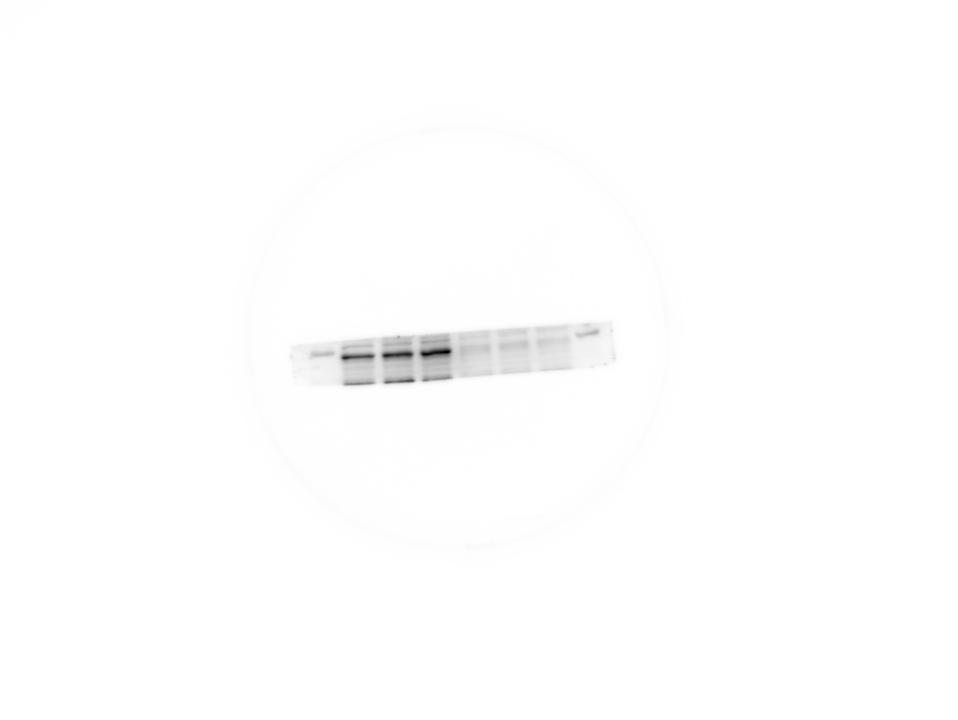

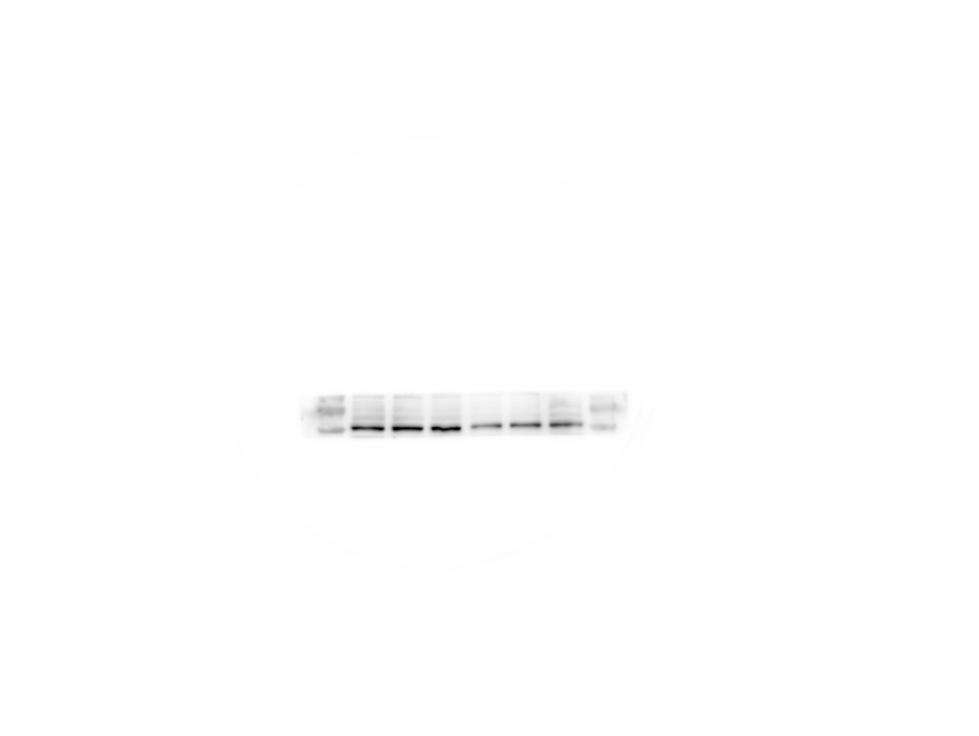


**Fig. 9 G: H3K4me1(KYSE30) Fig. 9 G: H3(KYSE30)**

**
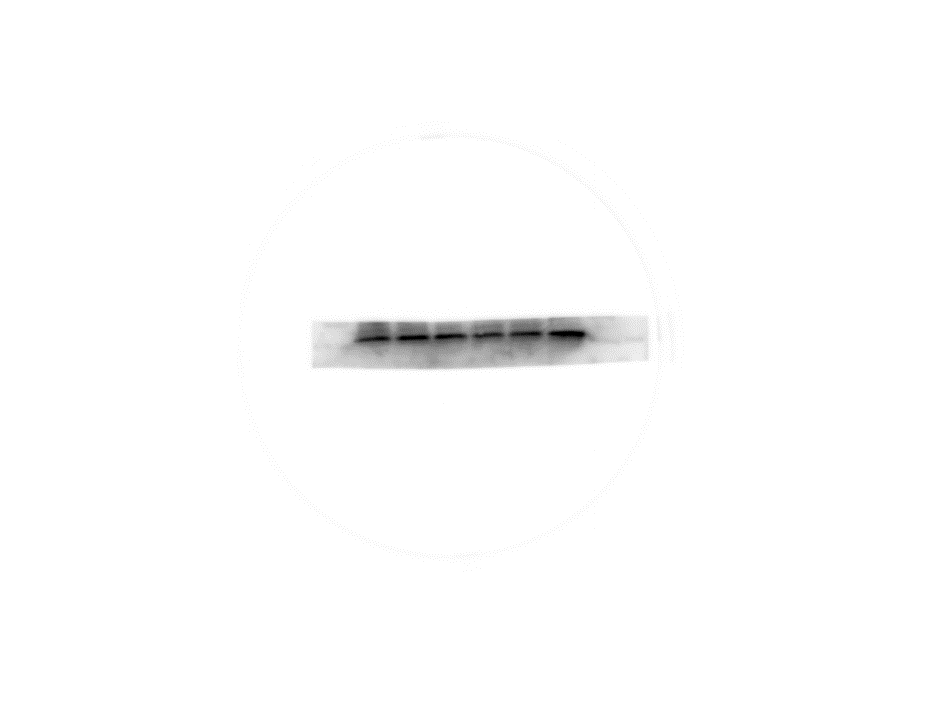
**
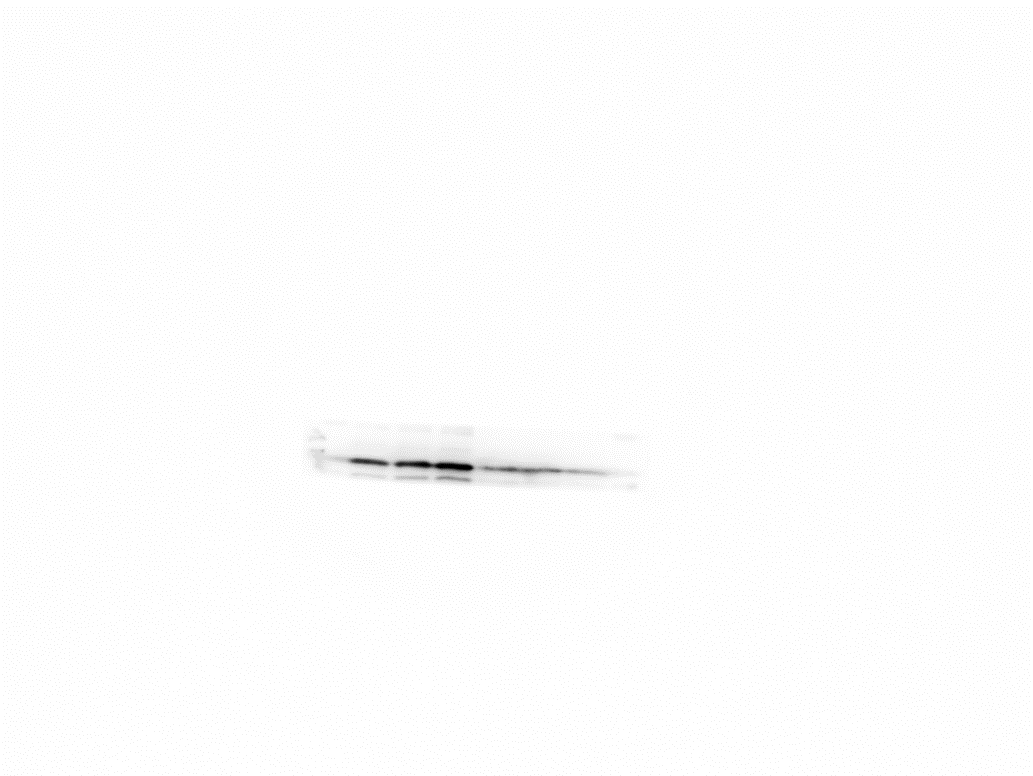


**Fig. 9 G: ALDH1A3(KYSE150) Fig. 9 G: SETD7(KYSE150) Fig. 9 G: ACTIN(KYSE150)**


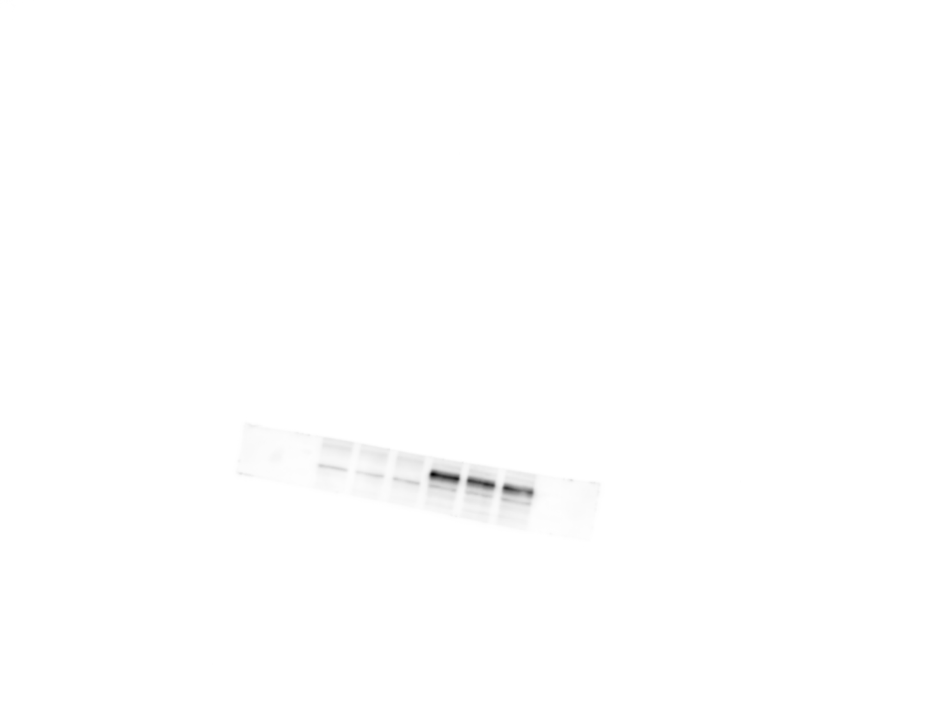
**
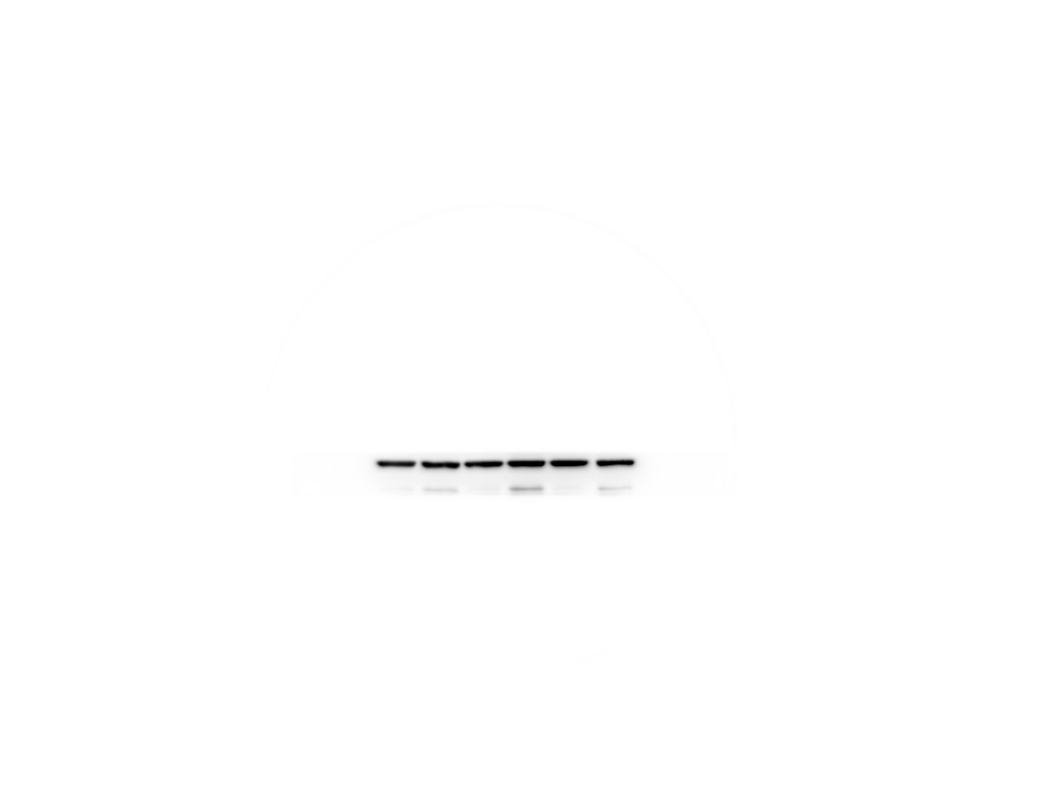
**
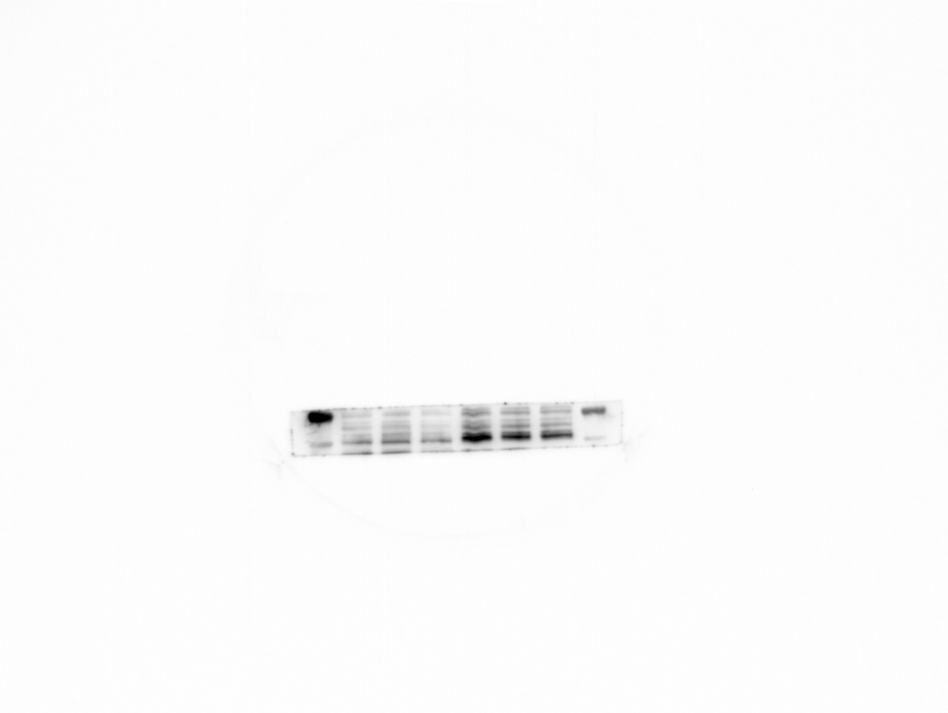


**Fig. 9 G: H3K4me1(KYSE150) Fig. 9 G: H3(KYSE150)**


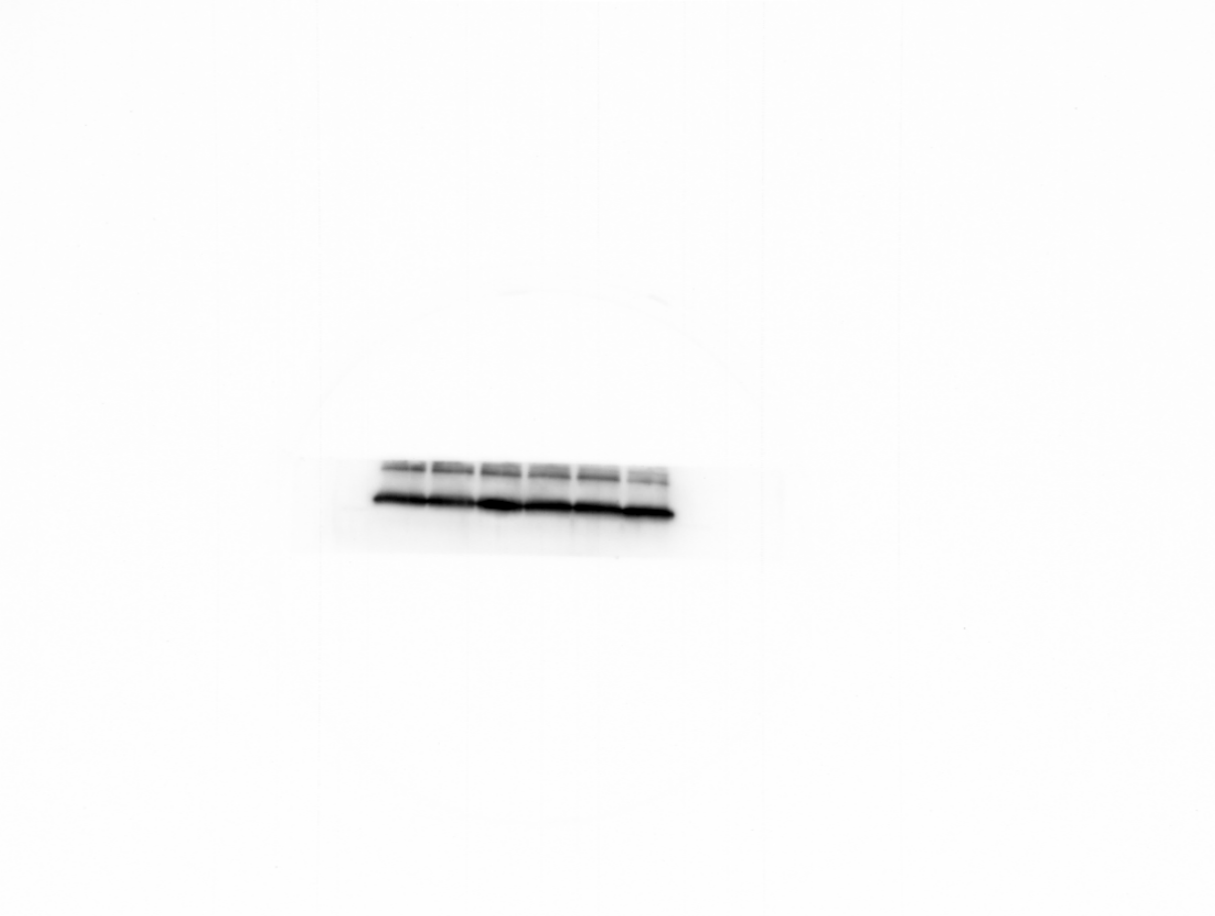

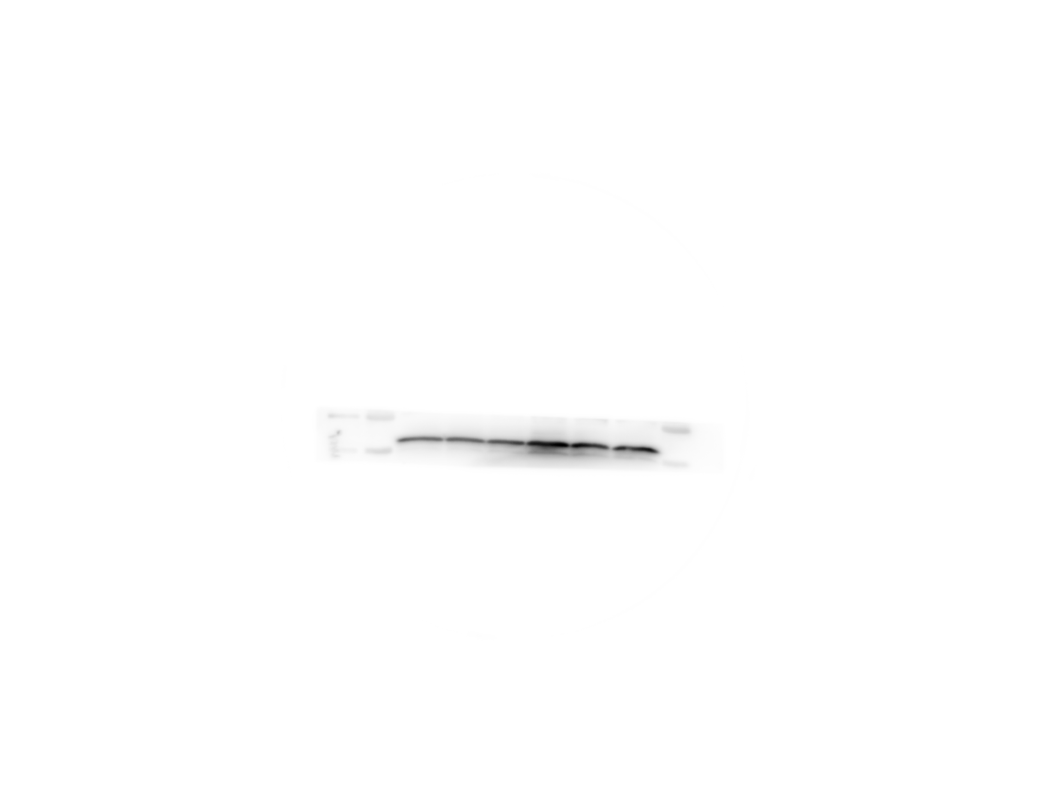


**Fig. S2 B: ALDH1A3 Fig. S2 B: SETD7 Fig. S2 B: ACTIN**


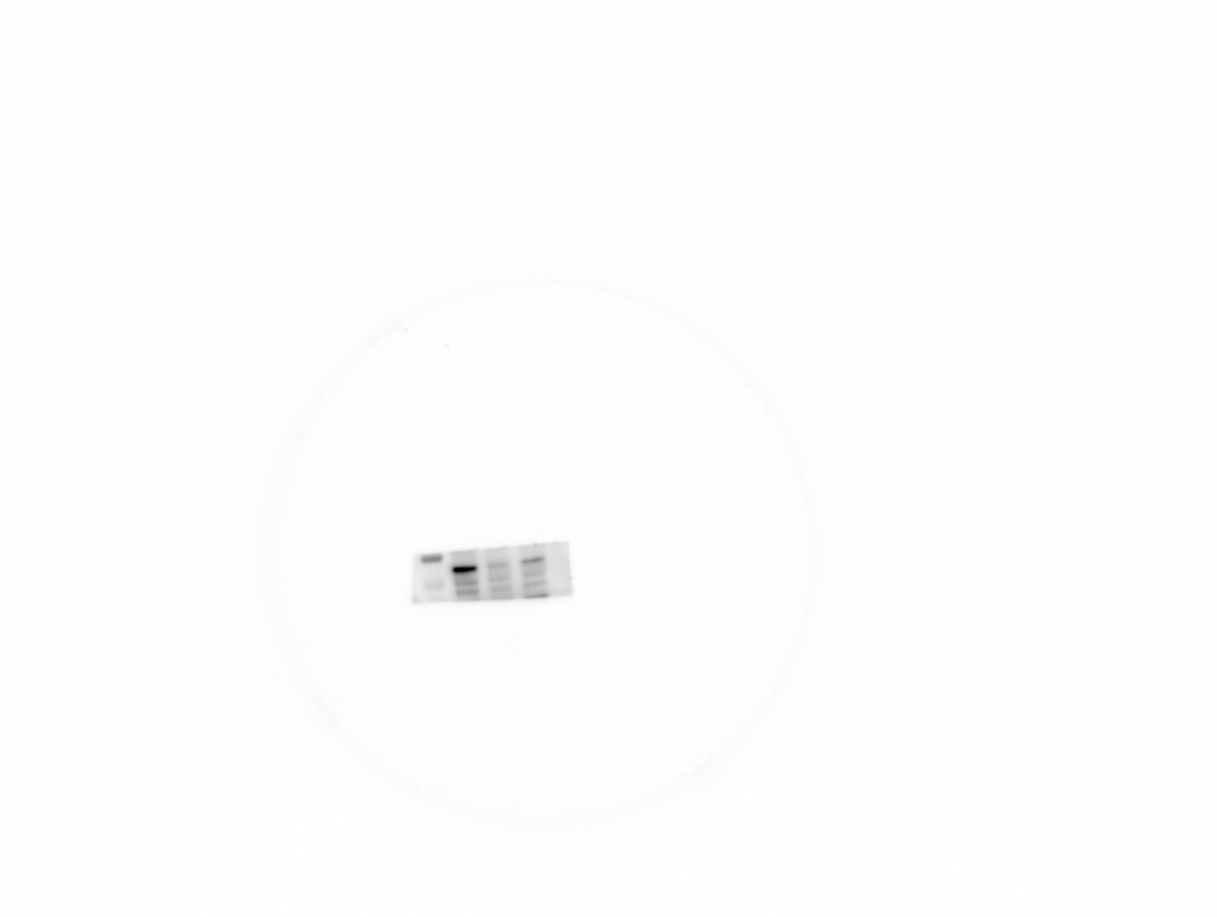

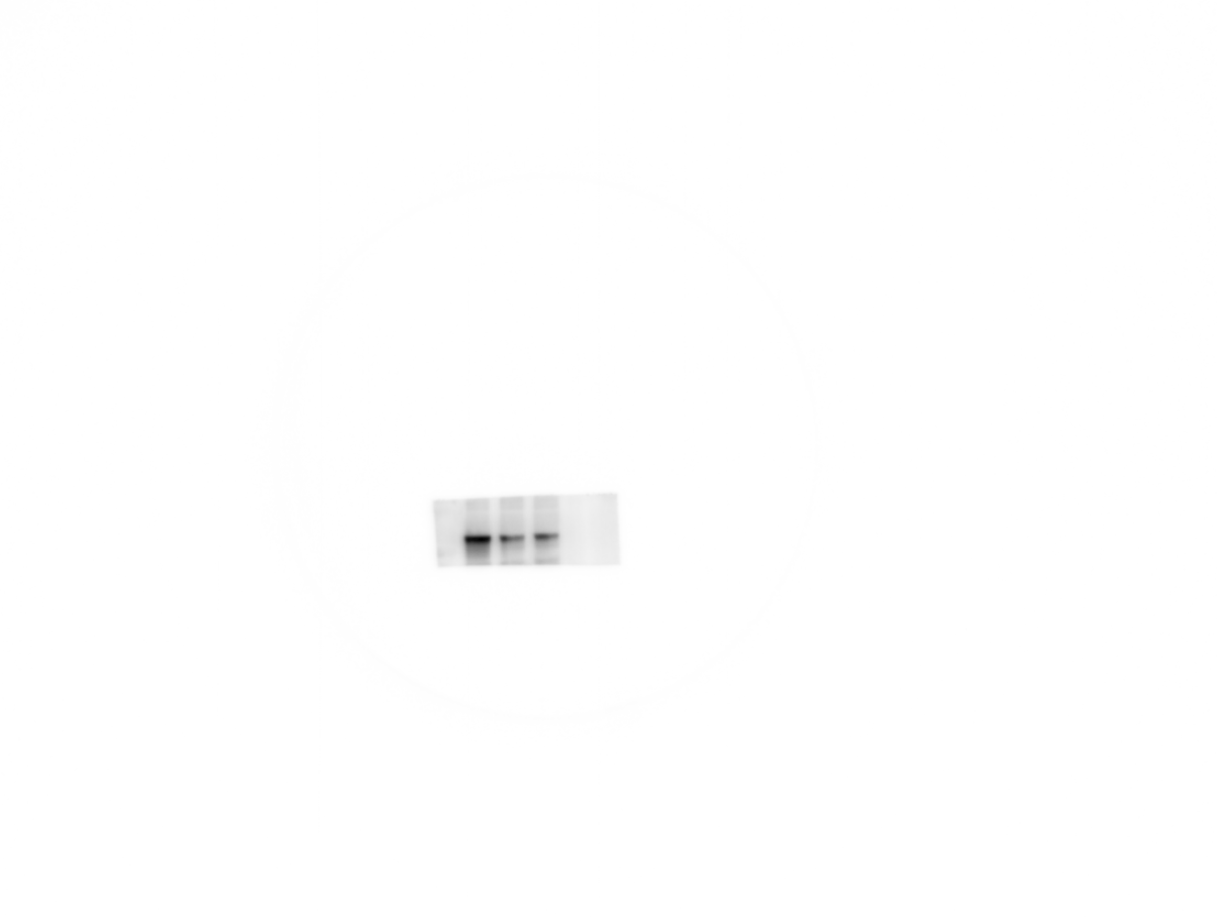

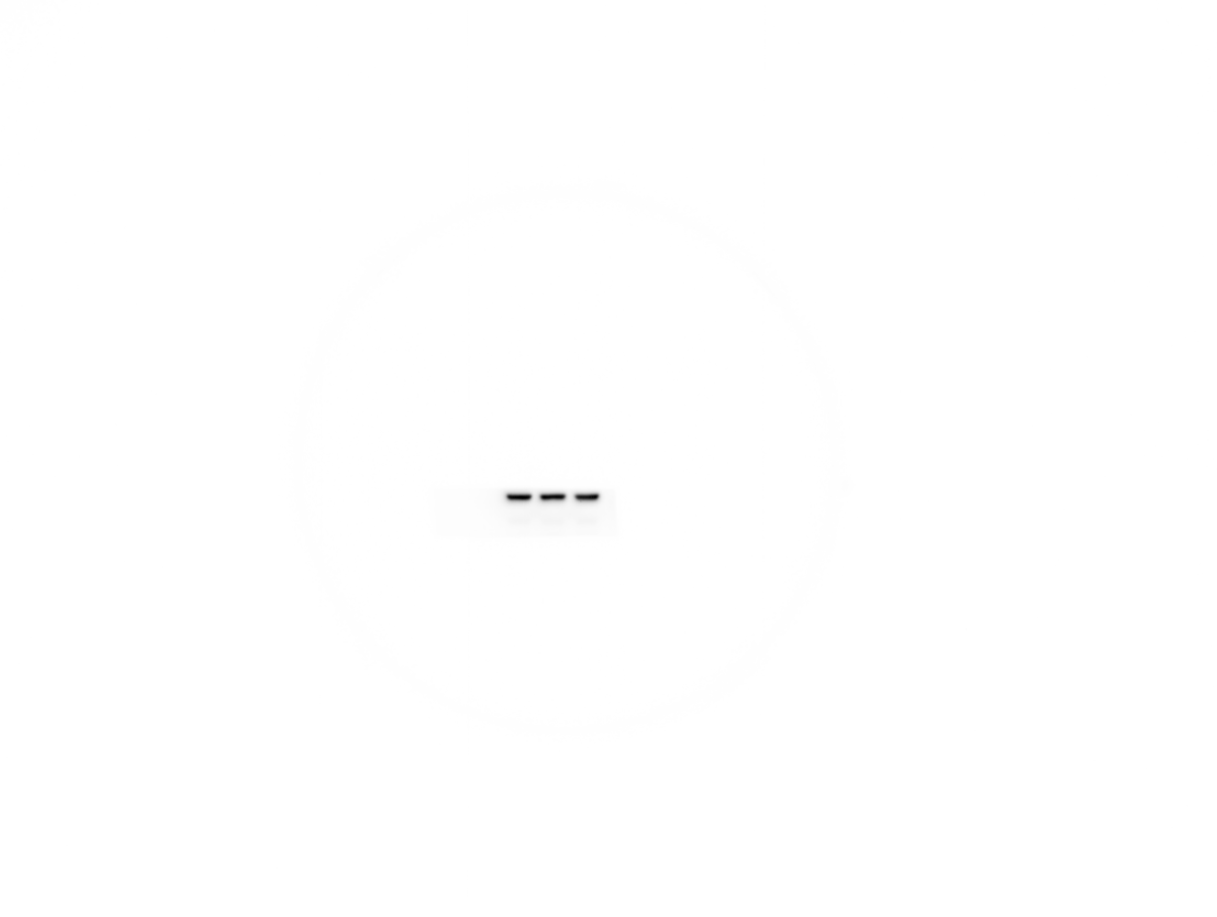


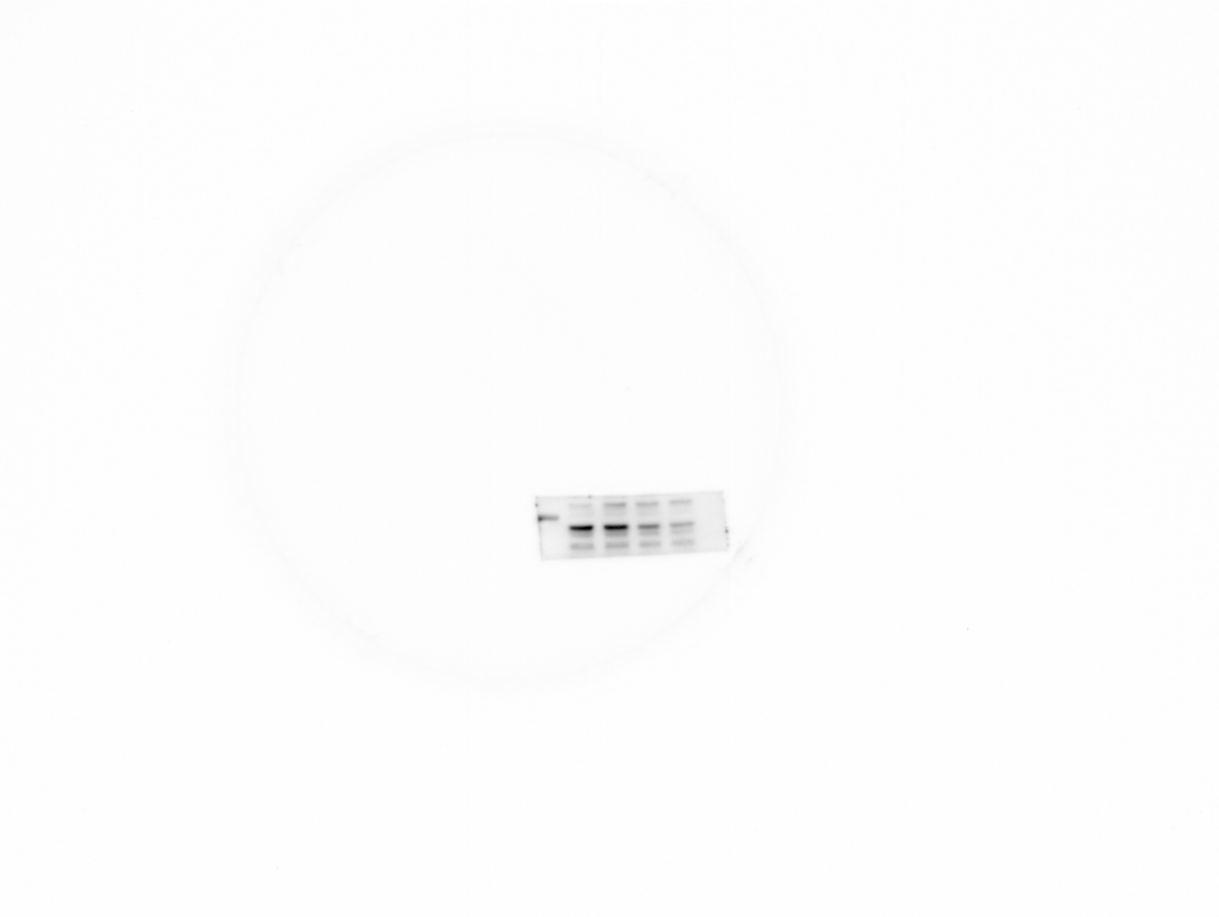

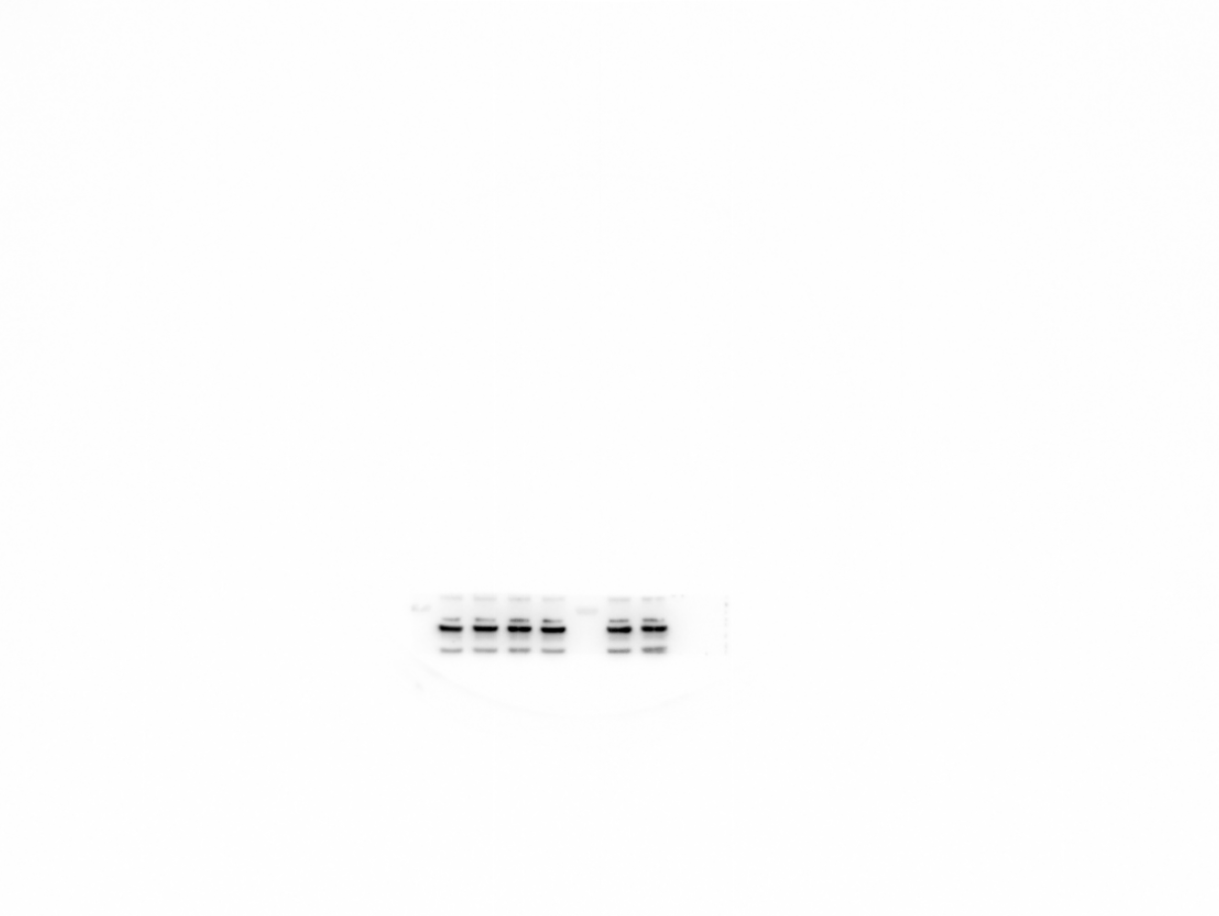

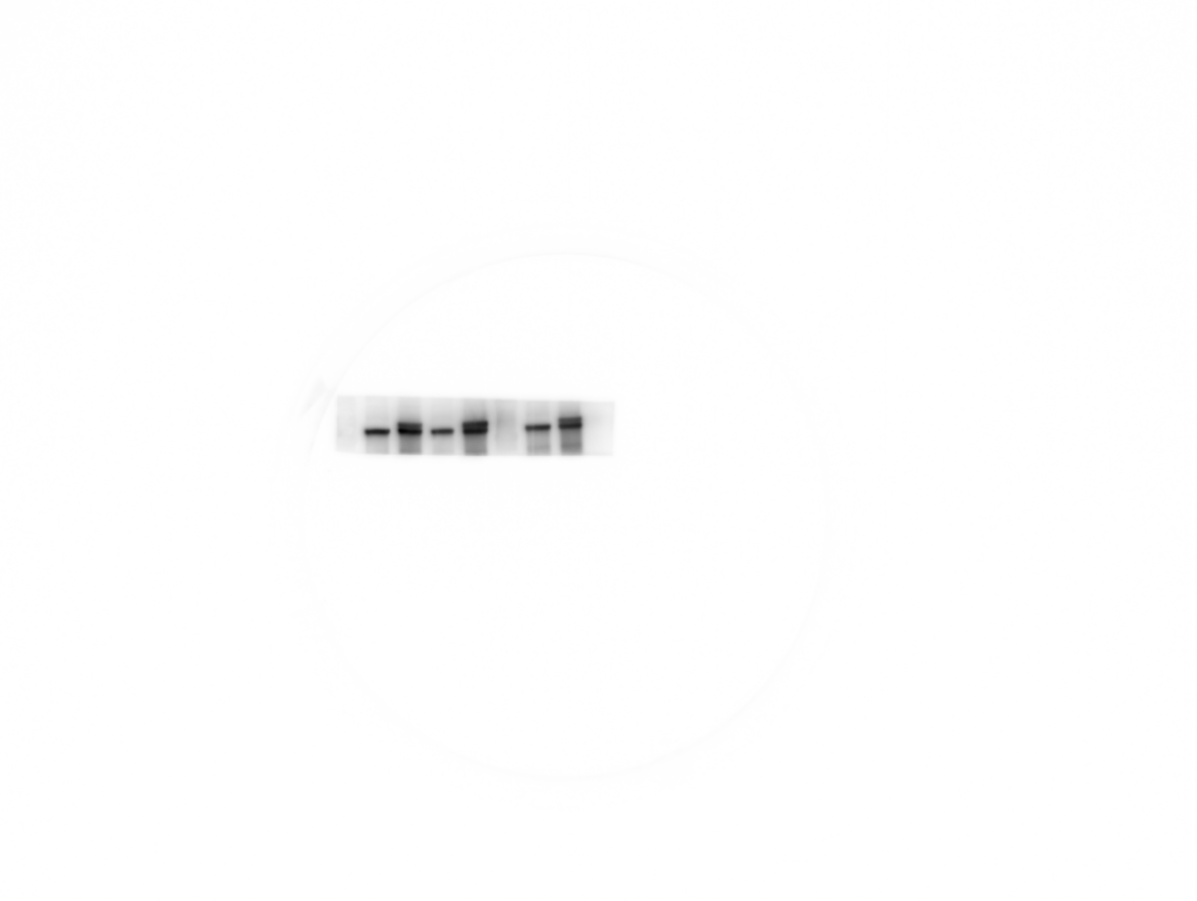
**Fig. S5 B: ALDH1A3 Fig. S5 B: SETD7 Fig. S5 B: ACTIN**
