# Supplementary material for: SETD7-mediated H3K4me1 activates ALDH1A3 to drive ferroptosis resistance in esophageal squamous cell carcinoma
Source: Cell Death Dis. 2025 Nov 7;16(1):810. doi: 10.1038/s41419-025-08133-7 (PMC12595051; doi:10.1038/s41419-025-08133-7)
Supplement: Supplementary file 2 — Supplementary figures [file 41419_2025_8133_MOESM2_ESM.docx]

**Supplementary figures**


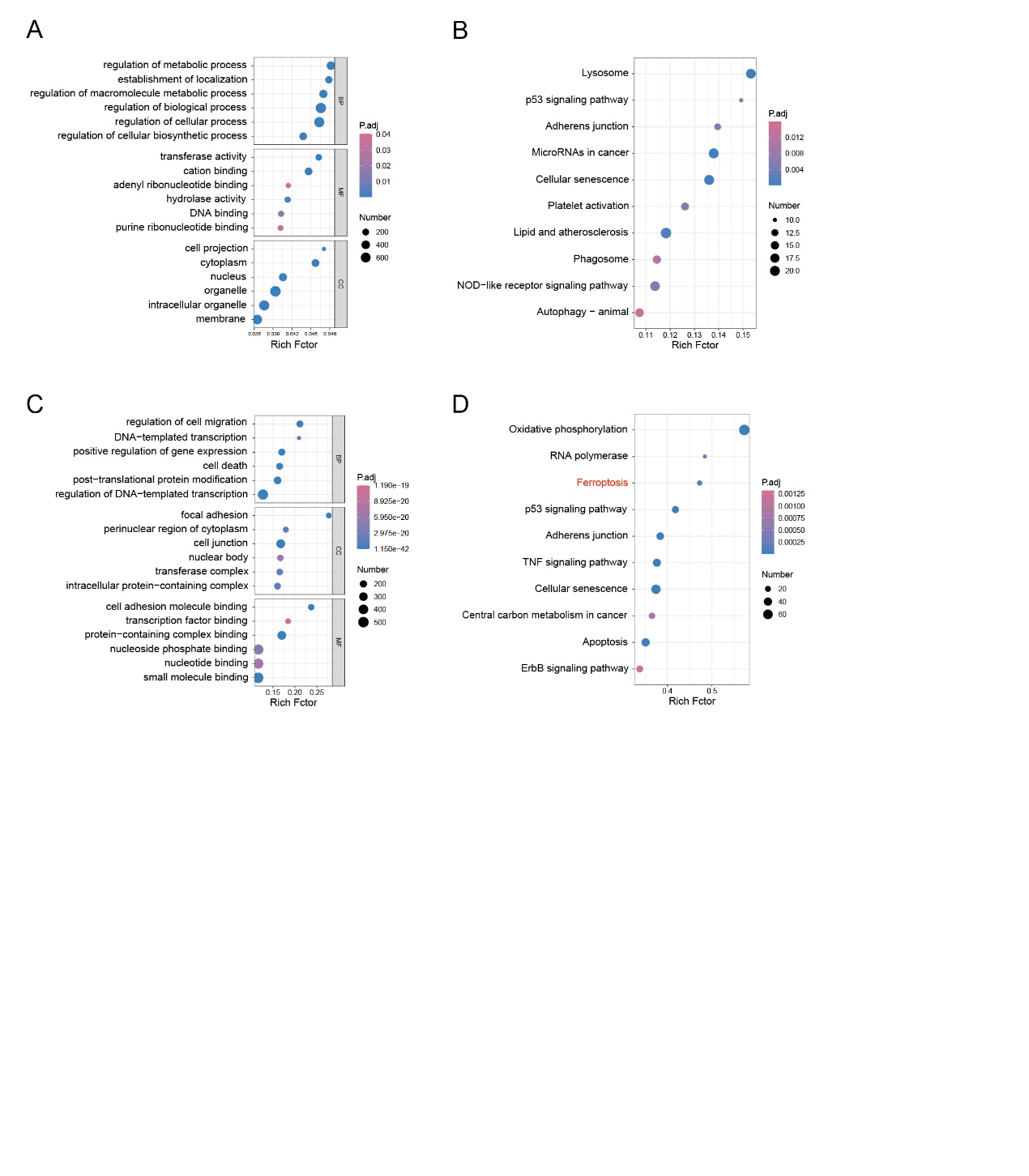
**Figure S1. GO and KEGG analyses of RNA-seq data.**

**A, B** GO (A) and KEGG (B) analyses of DEGs identified from RNA-seq data (sh-SETD7 vs. sh-NC). **C, D** GO (C) and KEGG (D) analyses of DEGs from RNA-seq data (SETD7 vs. EV).


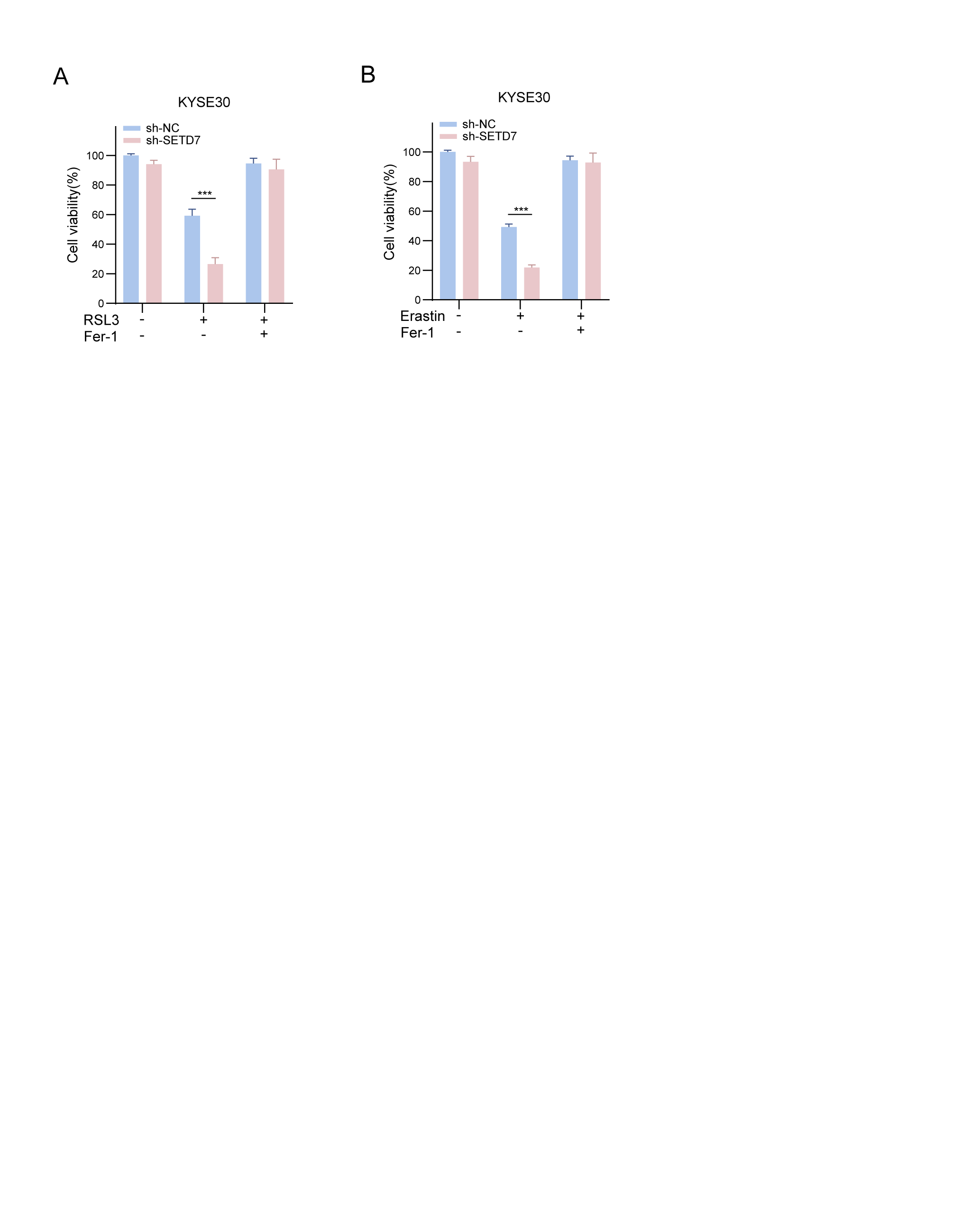
**Figure S2. SETD7 confers ferroptosis resistance in ESCC cells.**

**A, B** KYSE30 cell lines with SETD7 knockdown were treated with RSL3 (3 μM) or Erastin (10 μM) for 12 hours, and cell viability was assessed. Data are presented as means ± SD. **p* < 0.05, ***p* < 0.01, ****p* < 0.001, *****p* < 0.0001.


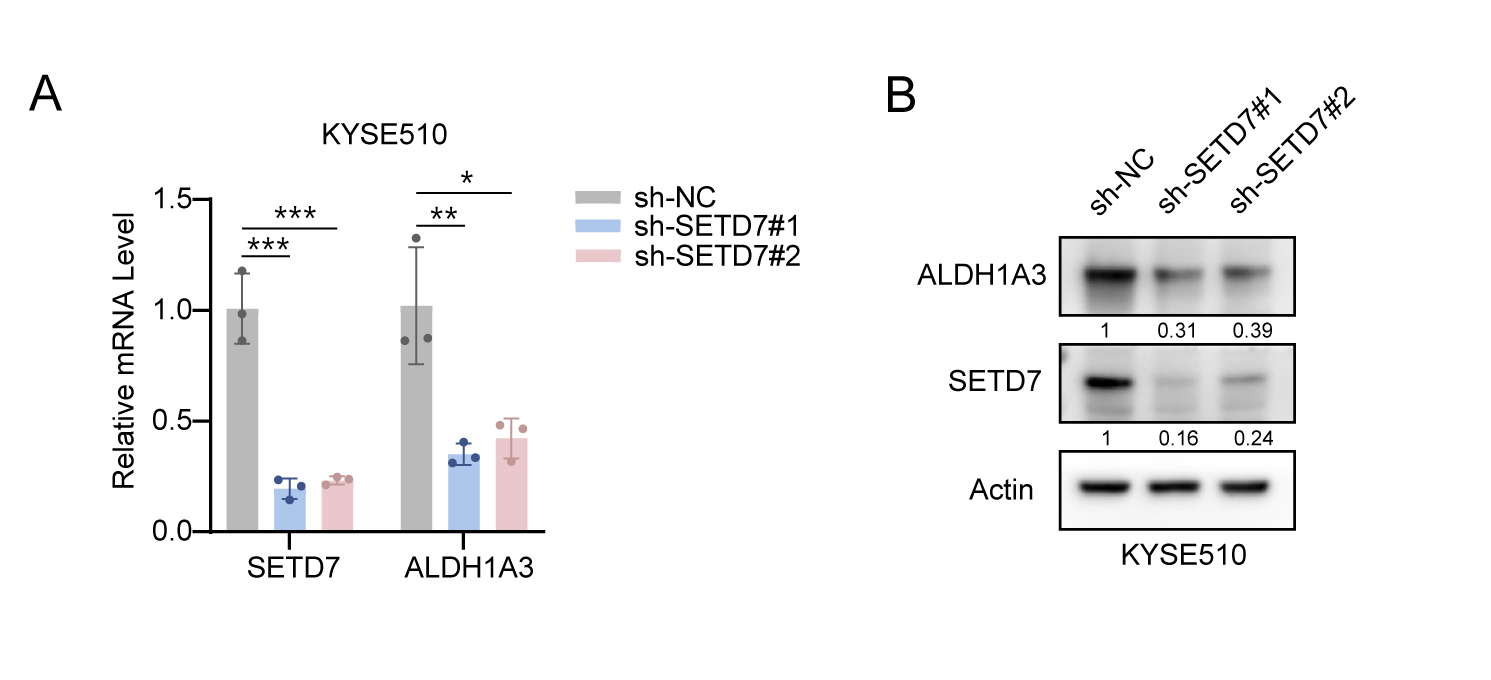


**Figure S3. SETD7 promotes ALDH1A3 transcription in ESCC cells.**

**A, B** RT-qPCR (A) and Western blotting (B) analyses were conducted to detect changes in ALDH1A3 mRNA and protein levels in ESCC cell lines with SETD7 knockdown. Data are presented as means ± SD. **p* < 0.05, ***p* < 0.01, ****p* < 0.001, *****p* < 0.0001.


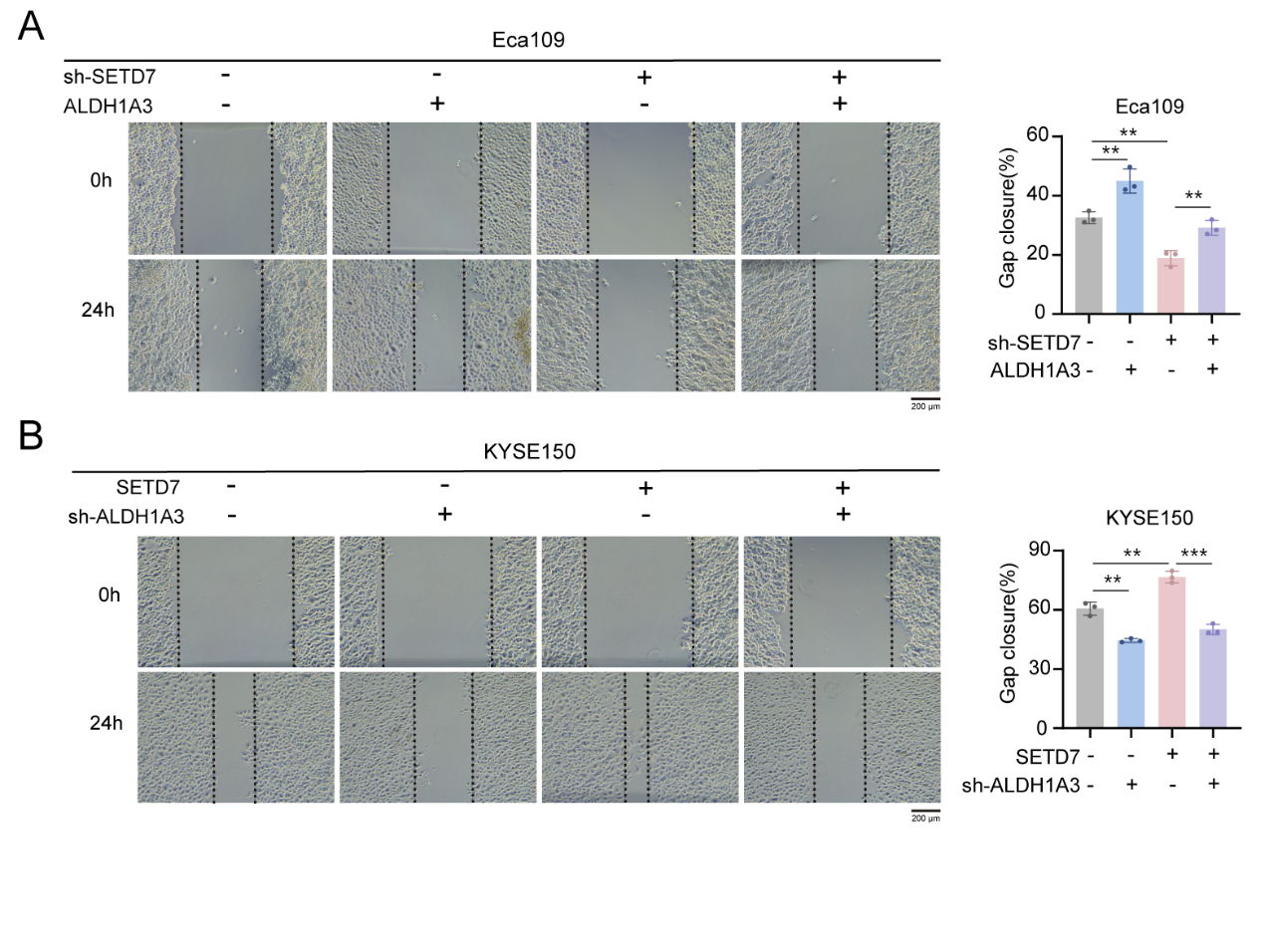


**Figure S4. SETD7 regulates the migration of ESCC cell through ALDH1A3.**

**A** Wound healing assay assessing the effect of ALDH1A3 overexpression on migration in SETD7-knockdown ESCC cells. Scale bar: 200 μm. **B** Wound healing assay evaluating the effect of ALDH1A3 knockdown on migration in SETD7-overexpressing ESCC cells. Scale bar: 200 μm. Data are presented as means ± SD. **p* < 0.05, ***p* < 0.01, ****p* < 0.001, *****p* < 0.0001.


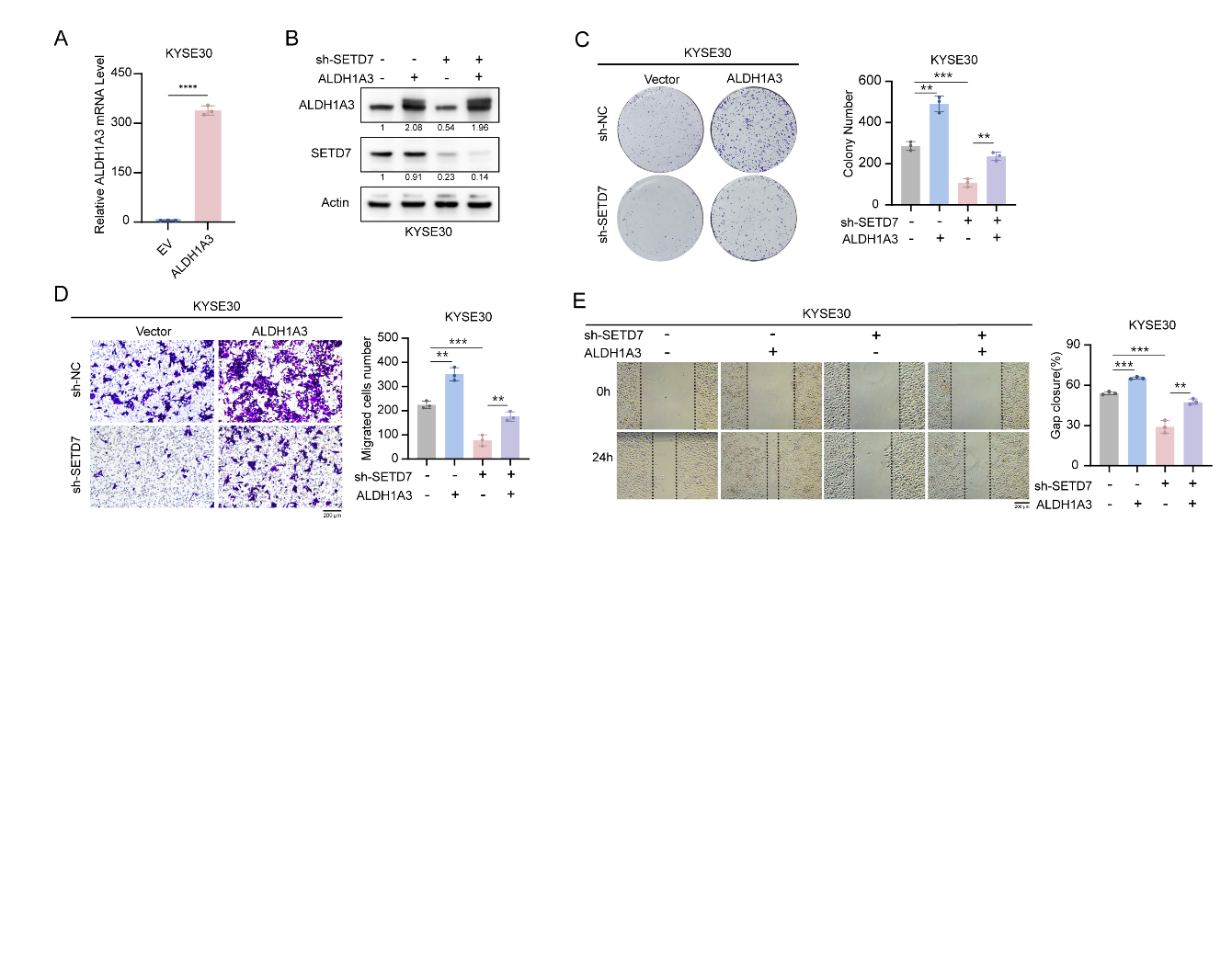


**Figure S5. SETD7 regulates ESCC cell proliferation and migration through ALDH1A3.**

**A, B** Validation of ALDH1A3 overexpression efficiency in KYSE30 cells by RT-qPCR (A) and Western blotting (B). **C** Colony formation assays assessing the effect of ALDH1A3 overexpression on proliferation in SETD7-knockdown ESCC cells. **D, E** Transwell and wound healing assays assessing the effect of ALDH1A3 overexpression on migration in SETD7-knockdown ESCC cells. Scale bar: 200 μm. Data are presented as means ± SD. **p* < 0.05, ***p* < 0.01, ****p* < 0.001, *****p* < 0.0001.
