# Supplementary material for: SETD7-mediated H3K4me1 activates ALDH1A3 to drive ferroptosis resistance in esophageal squamous cell carcinoma
Source: Cell Death Dis. 2025 Nov 7;16(1):810. doi: 10.1038/s41419-025-08133-7 (PMC12595051; doi:10.1038/s41419-025-08133-7)
Supplement: Supplementary file 3 — Supplementary tables [file 41419_2025_8133_MOESM3_ESM.docx]

**Supplementary tables**

| **Table S1. Sequences of shRNAs** | |  |
| --- | --- | --- |
| Target | Sequence (5'-3') | |
| shSETD7#1 | CATGGAGTGTGCTGGATATAT | |
| shSETD7#2 | GGTTCTGCACAGTCACCTACT | |
| shALDH1A3 | CCAAGAUAUUUAUCAACAA | |

| **Table S2. Primers used in qRT-PCR analyses** | |  |
| --- | --- | --- |
| Actin forward primer | CCTGGCACCCAGCACAAT | |
| Actin reverse primer | GGGCCGGACTCGTCATAC | |
| SETD7 forward primer | GGGCACGTATGTAGACGGAG | |
| SETD7 reverse primer | CAAGGCTTCCTCCATCTGGG | |
| ALDH1A3 forward primer | AGATACTTTGCAGGGTGGGC | |
| ALDH1A3 reverse primer | GGGGGAAGTTCCATGGAGTG | |

| **Table S3. Primers used in CUT&TAG analyses** | |  |
| --- | --- | --- |
| DNA Spike-in forward primer | GCCTTCTTCCCATTTCTGATCC | |
| DNA Spike-in reverse primer | CACGAATCAGCGGTAAAGGT | |
|  |  | |
| Primer1 forward primer | GTGCGCCGCAGACTAGG | |
| Primer1 reverse primer | GTTTTCCACGGCCCCGTTAG | |
|  |  | |
| Primer2 forward primer | GAGCGTTTCCCAAATCGCAA | |
| Primer2 reverse primer | TTGTCGCGCATTACTTCCCA | |
|  |  | |
| Primer3 forward primer | ACCATCAAGGACCCCCGATA | |
| Primer3 reverse primer | GAGGACCGTCAGGGAGTAGT | |
|  |  | |
| Primer4 forward primer | ACTCCCGCCATTTTTGACCT | |
| Primer4 reverse primer | AGGAGGGGCTTCTCTGACAT | |
|  |  | |
| Primer5 forward primer | GGGTGACCACCTGACTTCTG | |
| Primer5 reverse primer | TGTTTGCGGATGACCACTCA | |
